# Segmenting the Global Layers of Malignant Meningioma: A Population‐Based Study of Incidence, Risk Factors, and Temporal Trends

**DOI:** 10.1002/brb3.70430

**Published:** 2025-03-26

**Authors:** Junjie Huang, Lai Yim, Apurva Sawhney, Veeleah Lok, Lin Zhang, Xu Lin, Don Eliseo Lucero‐Prisno, Claire Chenwen Zhong, Wanghong Xu, Zhi‐Jie Zheng, Mellissa Withers, Martin C. S. Wong

**Affiliations:** ^1^ The Jockey Club School of Public Health and Primary Care, Faculty of Medicine Chinese University of Hong Kong Hong Kong SAR China; ^2^ Centre for Health Education and Health Promotion, Faculty of Medicine The Chinese University of Hong Kong Hong Kong SAR China; ^3^ Department of Global Public Health, Karolinska Institute Karolinska University Hospital Stockholm Sweden; ^4^ The School of Public Health and Preventive Medicine Monash University Victoria Australia; ^5^ School of Public Health The Chinese Academy of Medical Sciences and Peking Union Medical College Beijing China; ^6^ Department of Thoracic Surgery, The First Affiliated Hospital, School of Medicine Zhejiang University Hangzhou Zhejiang China; ^7^ Department of Global Health and Development London School of Hygiene and Tropical Medicine London UK; ^8^ School of Public Health Fudan University Shanghai China; ^9^ Department of Global Health, School of Public Health Peking University Beijing China; ^10^ Department of Population and Health Sciences Institute for Global Health University of Southern California Los Angeles USA

**Keywords:** incidence, malignant meningioma, risk factors, temporal trend

## Abstract

**Background:**

Malignant meningioma is a rare form of primary central nervous system cancer originating from the meninges membrane layers. Current data remain unmapped to cover trends for particular groups globally.

**Methods:**

This study examined the missing gap for its global burden, country‐specific incidence, and risk factor trends, stratified by sex and age. Several databases were retrieved for temporal trend analysis and interpretation: Cancer incidence rates from five continents (CI5 Plus), global cancer observatory (GLOBOCAN), global burden of disease (GBD), and the world bank. association between malignant meningioma's and various factors was determined using linear regression. Meningioma incidence trends were estimated using the average annual percentage change (AAPC) with join point regression, including the shift in cancerous meningioma incidence based on corresponding specific variables.

**Result:**

New malignant meningioma cases reported in 2020 were estimated to be 14,832 with aged‐standardized rates (ASR) of 1.3 per million population. Considerable variations exist among nations for malignant meningioma's, with the highest ASR found in Latvia (6.9 per million population), compared to a 345‐fold difference from the lowest ASR found in Fiji (0.02 per million population). Additionally, chronic disease presence such as smoking and hypertension was associated with higher malignant meningioma incidence. The analysis observed increasing rates of malignant meningioma in younger populations.

**Conclusions:**

Overall, this study contributes a global perspective on malignant meningioma incidence and emphasizes further investigation of specific groups that may have been overlooked. The increasing trend of malignant meningioma in younger populations warrants preventive, early diagnosis, and further research initiatives for evidence on risk management.

## Graphical abstract

This study revealed a wide variation of burden of malignant meningioma in global perspective, and emphasized further investigation of specific group which may have been overlooked. In addition, the study highlighted the role of lifestyle risk factor, such as smoking and hypertension, emphasized the need of health promotion and community intervention for specific group.

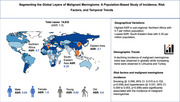

**Abbreviations**
AAPC: Average Annual Percentage Change
ASR: Age‐standardized rates
CI: Confidence Intervals
CI5: Five Continents
CI5 Plus: Five Continents Plus
CNS: Central Nervous System
GBD: Global Burden of Disease
GDP: Gross Domestic Product
GLOBOCAN: Global Cancer Observatory
HDI: Human Development Index
SD: Standard Deviation
WHO: World Health Organization
β: Beta coefficients

## 1 Introduction

Meningioma's are benign tumors of the primary central nervous system (CNS), with a rare probability of developing higher grades of malignancy (Ogasawara et al. 2021). World health organization (WHO) grading system for tumors assigns Grades 1 to 3 for meningioma's based on divergence from other cancer types, with the majority of meningioma cases remaining Grade 1 (Ogasawara et al. 2021). Recent literature has reported shifts in CNS trends globally; however, the scarcity of representative population data and the lack of differentiated data for individual CNS cancers limit the epidemiological scope for malignant meningioma in particular (Naslund et al. 2023). While the incidence of malignant meningioma accounted for a small proportion of all meningiomas, (Cao et al. 2022), malignant meningiomas could lead to severe complications and impact patient outcomes significantly, particularly concerning given the challenges in diagnosis and treatment. Understanding their epidemiology could help in developing better preventive strategies, despite lower incidence. Associated with its malignant nature, it has become imperative to enhance the diagnostics, monitoring, and management of malignant meningioma by evaluating global trends.

Ionizing radiation exposure is a well‐known environmental risk factor for meningioma, along with obesity, excessive alcohol consumption, and radiotherapy (Wiemels et al. 2010, Huang et al. 2023, Krampla et al. 2004, Schneider et al. 2005). In addition, given the wide‐ranging impact of socioeconomic factors on health outcomes, their potential relationship with meningioma incidence needs to be thoroughly explored. Previous studies have suggested that higher human development index (HDI) and gross domestic product (GDP) per capita may be associated with better health care, education, lifestyle, and early detection of cancer, which may indirectly affect incidence rates (Mrema et al. 2022, Bray et al. 2018, Benson et al. 2008, Mousavi et al. 2024). Endogenous and exogenous hormonal fluctuations in females, oral contraceptive pills, and hormone replacement therapy have been identified as significant risk factors (Wiemels et al. 2010). Individuals with a history of breast cancer have a 10‐times‐higher incidence of meningioma's (Degeneffe et al. 2023). Other factors associated with meningioma include a higher prevalence among females compared to men, older age, and certain ethnic populations (Ogasawara et al. 2021, Naslund et al. 2023, Achey et al. 2018). However, there is still a need to explore additional variables that may be associated with malignant meningioma's, including chronic diseases. While some literature has examined the epidemiology of meningioma, previous studies have often focused on specific geographical locations (Achey et al. 2018), age groups (Achey et al. 2018), sexes (Benson et al. 2008, Lee et al. 2006, Schneider et al. 2005) or used relatively old data (Benson et al. 2008, Klaeboe et al. 2005).

This study, hence, addresses research gaps by using recent, high‐quality data from international and national cancer registries to comprehensively examine the global burden and incidence of malignant meningioma. The analysis includes stratification by region, country, sex, age group, and country‐level risk factor analysis.

## 2 Methods

The 10‐year malignant meningioma incidence data were obtained from the CI5 Plus database, which includes annual incidences from over 100 cancer registries across various populations. This comprehensive database provides both recent and historical data, enabling a robust evaluation of temporal trends and interpretations (Cancer Incidence in Five Continents plus: IARC CancerBase No. 9 2020). Malignant meningioma incidence data were estimated from the Global Cancer Observatory (GLOBOCAN) database, which encompasses 185 countries, 26 types of cancer, and age stratification, using proportion estimations from the Cancer Incidence in Five Continents (CI5) (Ferlay et al. 2020). Selected data allowed estimation of incidence‐to‐mortality ratios, trends, and collective approximation for neighboring countries (Sung et al. 2021). This study followed the classification of UN Regions from Cancer Today of the WHO, which Cancer Today was part of the GLOBOCAN project (International Agency for Research on Cancer—Cancer Today 2024, International Agency for Research on Cancer—Cancer Today 2024).

Risk factor data were sourced from the GBD database (Institute for Health Metrics and Evaluation (IHME) 2019), which includes country‐specific information on smoking, alcohol consumption, unhealthy eating, physical inactivity, obesity, hypertension, diabetes, and lipid disorders. Finally, the HDI and GDP per capita data were collected from the WHO and the world bank for subsequent statistical analysis. (UNDP 2018; Bank 2020) Therefore, the association between malignant meningioma incidence and potential risk factors (HDI, GDP per capita, lifestyle, and metabolic factors) was examined using linear regression analysis. In this study, different databases were used for different purposes: GLOBOCAN was used to assess the latest global burden; CI5 was used to examine cancer incidence and historical trends over a 10‐year period; and GBD was used for risk factor analysis. This separation ensures the integrity of each dataset while enabling us to draw nuanced conclusions about the burden, trends, and risk factors associated with malignant meningioma. The analysis was stratified by country, age, and sex. Beta coefficients (β) with their corresponding 95% confidence intervals (95% CI) were calculated to provide standardized estimates of effect size. These coefficients represent the change in the incidence of meningioma (ASR) for every unit increase in the predictor variable (risk factor). A predetermined level of statistical significance (*p* < 0.005) was used for the analysis.

The Joinpoint regression program (Ver. 5.0.2) developed by the SEER Program of the National Cancer Institute of the United States, was used for trend analysis of meningioma incidence. (Jointpoint regression program) The Average Annual Percentage Change (AAPC) was calculated to assess the recent 10‐year incidence trends at various country and regional levels (Kim et al. 2000). Recent 10‐year incidence data follow standard practice in cancer epidemiology research (Huang et al. 2023). For analysis, incidence data were logarithmically transformed, processed, and presented with associated standard errors. A positive AAPC indicates a rising temporal trend in meningioma incidence, while a negative AAPC suggests a declining trend. The 95% confidence interval (CI) provides accuracy for these estimations, and an interval overlapping with 0 suggests a steady trend without significant increases or decreases. Meningioma incidence alterations were analyzed according to age groups (all population: 0–85+, young population: 15–49, older population: 50–74), sex (male, female), and continents (Asia, Oceania, America, Europe, Africa).

All data tables were compiled and are referenced in the supplementary section.

## 3 Results

### 3.1 Malignant Meningioma Incidence in 2020

**FIGURE 1:**
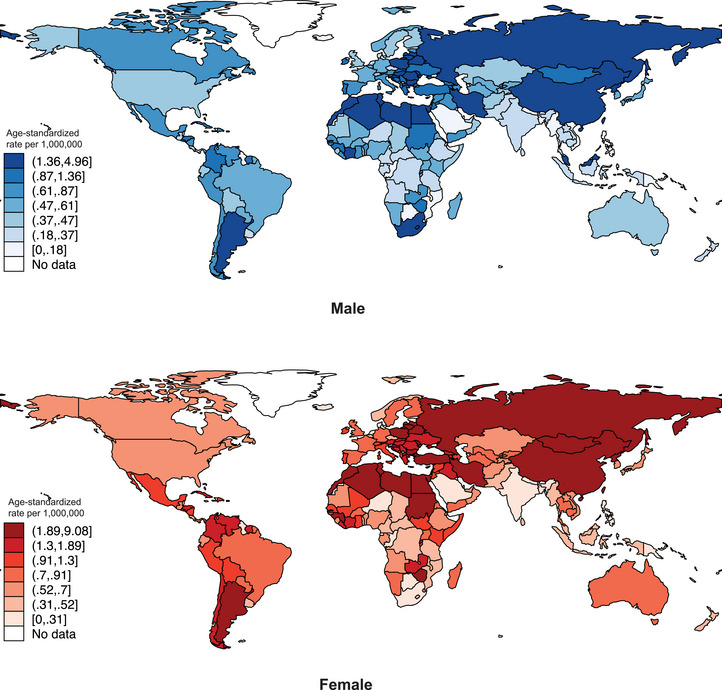
Global incidence of malignant meningioma by sex, all age, in 2020.

**FIGURE 2:**
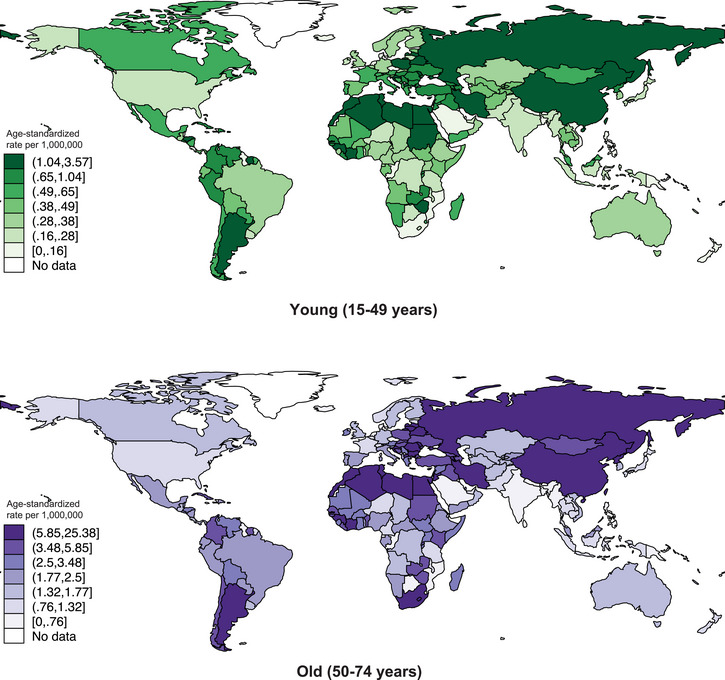
Global incidence of malignant meningioma by age, all sex, in 2020.

In 2020, newly reported malignant meningioma cases totaled to 14,832 globally with an ASR of 1·3 per 1 million population (Figure 1, Figure 2, Supplementary Table S1a, Supplementary Table S1b). Considering subregional variations, the highest ASR was found in Northern Africa (4.7), followed by Eastern Asia (2.7), Central and Eastern Europe (1.8), and South America (1.3). Conversely, the lowest ASR was found in Southeastern Asia (0.35), followed by South‐Central Asia (0.37), Sub‐Saharan Africa (0.64), Northern Europe (0.65) as well as Western Europe (0.68). Country‐level variations in ASR were highest in Latvia (6.9), followed by Egypt (5.8), Algeria (4.8), Morocco (4.8), and China (4.6). The lowest country‐level ASR was found in Fiji (0.02), followed by (in ascending order): Guyana (0.04), Saudi Arabia (0.07), Solomon Islands (0.08), and Haiti (0.10). Countries with the highest and lowest ASR variations (Latvia and Fiji, respectively) were separated by a 345‐fold difference.

### 3.2 Malignant Meningioma Incidence by Subgroup in 2020

On a global scale, the ASR of male incidence was 0.93 per million population in 2020 (5,044 new cases reported), lower than that of females (ASR = 1.6, per million population, 9,788 new cases reported) (Figure 1, Figure 2, Supplementary Table S1). Such differences were largely consistent among sub regions. Sub regionally, the highest incidence of males was observed in Northern Africa (3.2 vs. 6.0 in females), followed by Eastern Asia (1.8 vs. 3.6), Central and Eastern Europe (1.5 vs. 2.0), and Southern Europe (0.97 vs. 1.1). The lowest incidence of males was found in Southeastern Asia (0.21, vs. 0.45), South‐Central Asia (0.31 vs. 0.41), Central America and Caribbean (0.40 vs 0.99), and Sub‐Saharan Africa (0.46 vs. 0.75).

Country‐specific analysis showed the highest incidence of males was found in North Macedonia (5.0 vs. 2.5 in females), followed by Latvia (4.0 vs. 9.1), Egypt (3.9 vs. 7.5), Morocco (3.6 vs. 5.3), and Libya (3.5 vs. 4.6). Lowest incidence of males was found in Fiji (0.02 vs. 0.03 in females), followed by the Philippines (0.04 vs. 0.19), Solomon Islands (0.06 vs. 0.08), Guatemala (0.06 vs. 0.65), and Lesotho (0.06 vs. 0.33).

Older age groups presented higher numbers of newly reported cases, with 9,491 new cases in the 50–74 years group and 3,083 new cases in the 15–45 years age group (Figure 2). The ASR in 2020 was higher among the older population (4.6 per 1 million population) compared to the younger population (0.62 per 1 million population).

Corresponding ASR values between sub regions show Northern Africa to have the highest ASR among the young population (2.9) compared to the old population (17.0). This was followed by Eastern Asia (1.2 vs. 9.1), Central and Eastern Europe (0.98 vs. 5.3), and South America (0.75 vs. 4.3). On the other hand, lowest ASR among the younger population was found in South‐Central Asia (0.23 vs. 1.0 in the older population), followed by Southeastern Asia (0.24 vs. 1.0), Northern Europe (0.35 vs. 1.7), and Western Europe (0.39 vs. 1.5).

Among individual countries, older population in Latvia had the highest ASR (25.4 vs. 3.1 in the younger age group), followed by Egypt (19.7 vs. 3.6), Morocco (18.7 vs. 2.6), Libya (18.3 vs. 2.7), and Algeria (16.7 vs. 3.2). Conversely, the Solomon Islands had the lowest ASR among the older population (0·12 vs 0·08 in the younger age group), followed by Vanuatu (0.25 vs. 0.18), Guyana (0.25 vs. 0.0), Papua New Guinea (0.32 vs. 0.07), and Kuwait (0.34 vs. 0.06).

### 3.3 Associated Risk Factors for Malignant Meningioma

**FIGURE 3:**
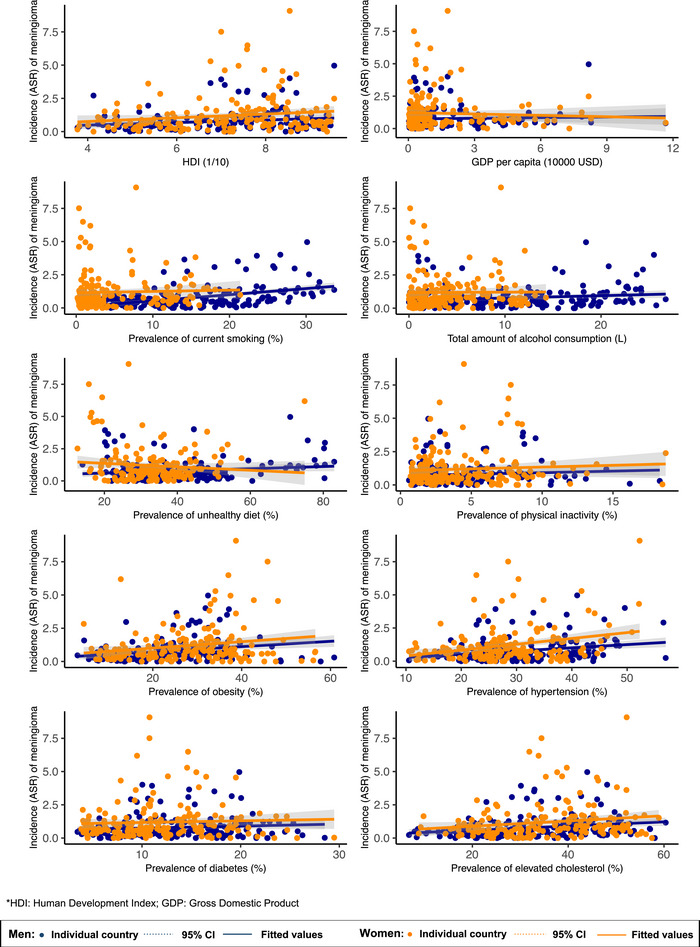
Association between risk factors and malignant meningioma by sex.

**FIGURE 4:**
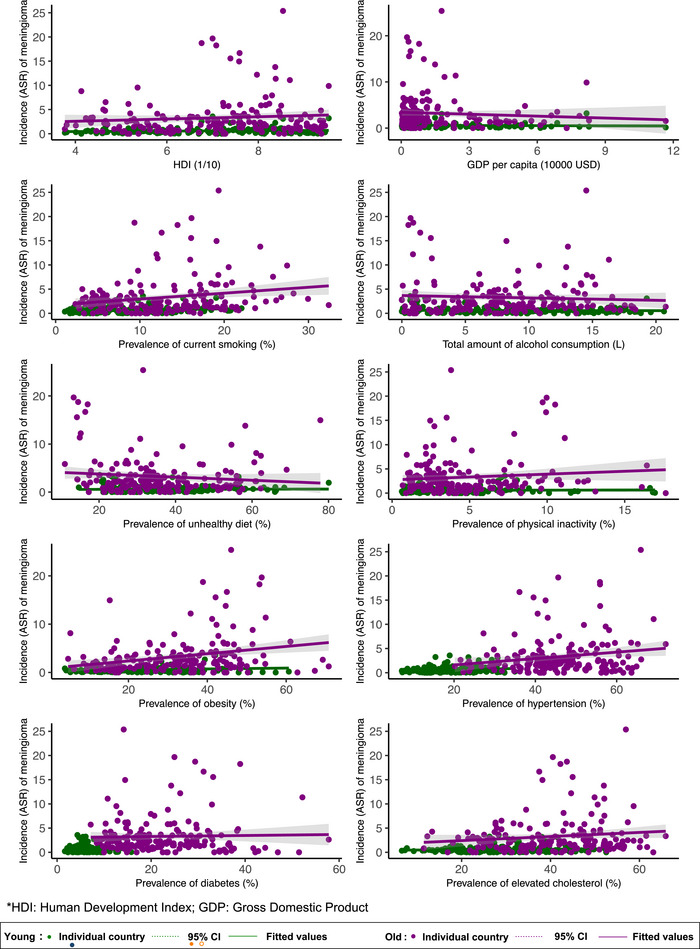
Association between risk factors and malignant meningioma by age.

Overall, higher malignant meningioma incidence was associated with higher HDI (*β* = 0.125, 95% CI [0.018, 0.232], *p* = 0.023), a higher prevalence of smoking (*β* = 0.058, 95% CI [0.030, 0.086], *p* < 0.001), obesity (*β* = 0.227, 95% CI [0.008, 0.037], *p* = 0.003), hypertension (*β* = 0.036, 95% CI [0.017, 0.054], *p* < 0.001), and lipid disorder (*β* = 0.019, 95% CI [0.005, 0.032], *p* = 0.008) (Figure 3, Figure 4, Supplementary Table S2).

In males, higher malignant meningioma incidence was associated with higher HDI (*β* = 0.095, 95% CI [0.014, 0.177], *p* = 0.022), a higher prevalence of smoking (*β* = 0.046, 95% CI [0.032, 0.061], *p* < 0.001), alcohol drinking (*β* = 0.018, 95% CI [0.001, 0.035], *p* = 0.043), obesity (*β* = 0.020, 95% CI [0.009, 0.031], *p* = 0.001), hypertension (*β* = 0.024, 95% CI [0.011, 0.038], *p* < 0.001), and lipid disorder (*β* = 0.016, 95% CI [0.006, 0.026], *p* = 0.002) (Supplementary Table S2).

For females, higher incidence of malignant meningioma was associated with higher HDI (*β* = 0.139, 95% CI [0.006, 0.272], *p* = 0.040), a higher prevalence of obesity (*β* = 0.024, 95% CI [0.006, 0.042], *p* = 0.008), hypertension (*β* = 0.044, 95% CI [0.021, 0.067], *p* < 0.001), and lipid disorder (*β* = 0.020, 95% CI [0.002, 0.037], *p* = 0.028) (Figure 3).

By age, among the younger population, a higher malignant meningioma incidence was associated with a higher prevalence of smoking (*β* = 0.029, 95% CI [0.011, 0·046], *p* = 0.002), obesity (*β* = 0.009, 95% CI [0.005, 0.017], *p* = 0.037), and hypertension (*β* = 0.018, 95% CI [0.002, 0.033], *p* = 0.025) (Figure 4).

For the older population, a higher prevalence of smoking (*β* = 0.122, 95% CI [0.033, 0.211], *p* = 0.007), obesity (*β* = 0.075, 95% CI [0.034, 0.117], *p* < 0.001), and hypertension (*β* = 0.066, 95% CI [0.012, 0.128], *p* = 0.017) were the risk factors for a higher incidence of malignant meningioma (Supplementary Table S2a).

In Multivariable analysis on risk factors and malignant meningioma incidence, distinct risk factors have been observed in different groups (Supplementary Table S2b). Overall, smoking (*β*: 0.059, 95% CI: 0.015 to 0.102, *p* = 0.008) and hypertension (*β*: 0.031, 95% CI: 0.009 to 0.053, *p* = 0.006) were significantly associated with the incidence of malignant meningioma. However, only smoking (*β*: 0.044, 95% CI: 0.024 to 0.063, *p* < 0.001) was associated with the incidence of malignant meningioma. As for female, other three risk factors, HDI (*β*: 0.234, 95% CI: 0.014 to 0.454, *p* = 0.038), GDP per capita (*β*: ‐0.157, 95% CI: ‐0.309 to ‐0.004, *p* = 0.044) and hypertension (*β*: 0.050, 95% CI: 0.024 to 0.075, *p* < 0.001) have shown significant association with incidence of malignant meningioma. Notable, both younger population and older population shown significant association for smoking and alcohol drinking to the incidence of malignant meningioma. In addition, obesity (*β*: 0.065, 95% CI: 0.005 to 0.125, *p* = 0.034) also been the risk factor for older populations.

### 3.4 Malignant Meningioma Incidence Trend Analysis by Sub‐group, Age, and Sex

Malignant meningioma incidence shows a declining trend overall (Supplementary Table S3). Six countries were identified with significant decreasing trends (mostly European countries), while two countries presented an ascending trend. Iceland reported the most significant decrease (AAPC: ‐18.37, 95% CI [‐31.26, ‐3.05], *p* = 0.021), followed by Germany (AAPC: ‐13.14, 95% CI [‐21.53, ‐3.84], *p* = 0.013), Austria (AAPC: ‐12.96, 95% CI [‐21.11, ‐3.98], *p* = 0.012), Croatia (AAPC: ‐11.34, 95% CI [‐17.54, ‐4.67], *p* = 0.005), the United States (AAPC: ‐5.63, 95% CI [‐10.18, ‐0.85], *p* = 0.027), and China (AAPC: ‐3.37, 95% CI [‐6.37, ‐0.28], *p* = 0.036). In contrast, a significant increase was found in Lithuania (AAPC: 14.16, 95% CI [8.19, 20.47], *p* < 0.001) and Turkey (AAPC: 5.66, 95% CI [0.41, 11.18], *p* = 0.038). The other 33 countries were observed with no significant change during this period.

**FIGURE 5:**
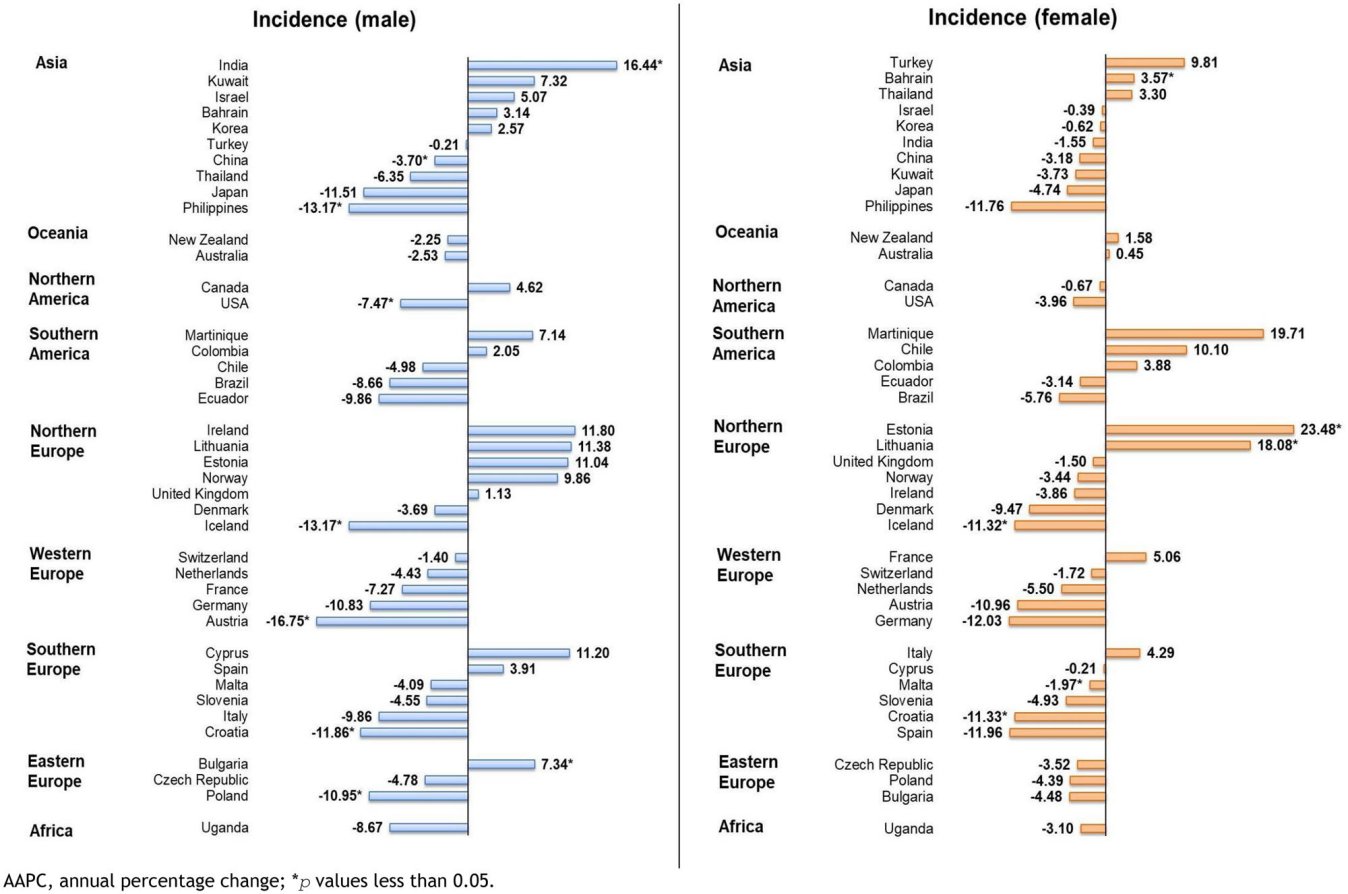
AAPC of malignant meningioma incidence by sex group, all ages.

**FIGURE 6:**
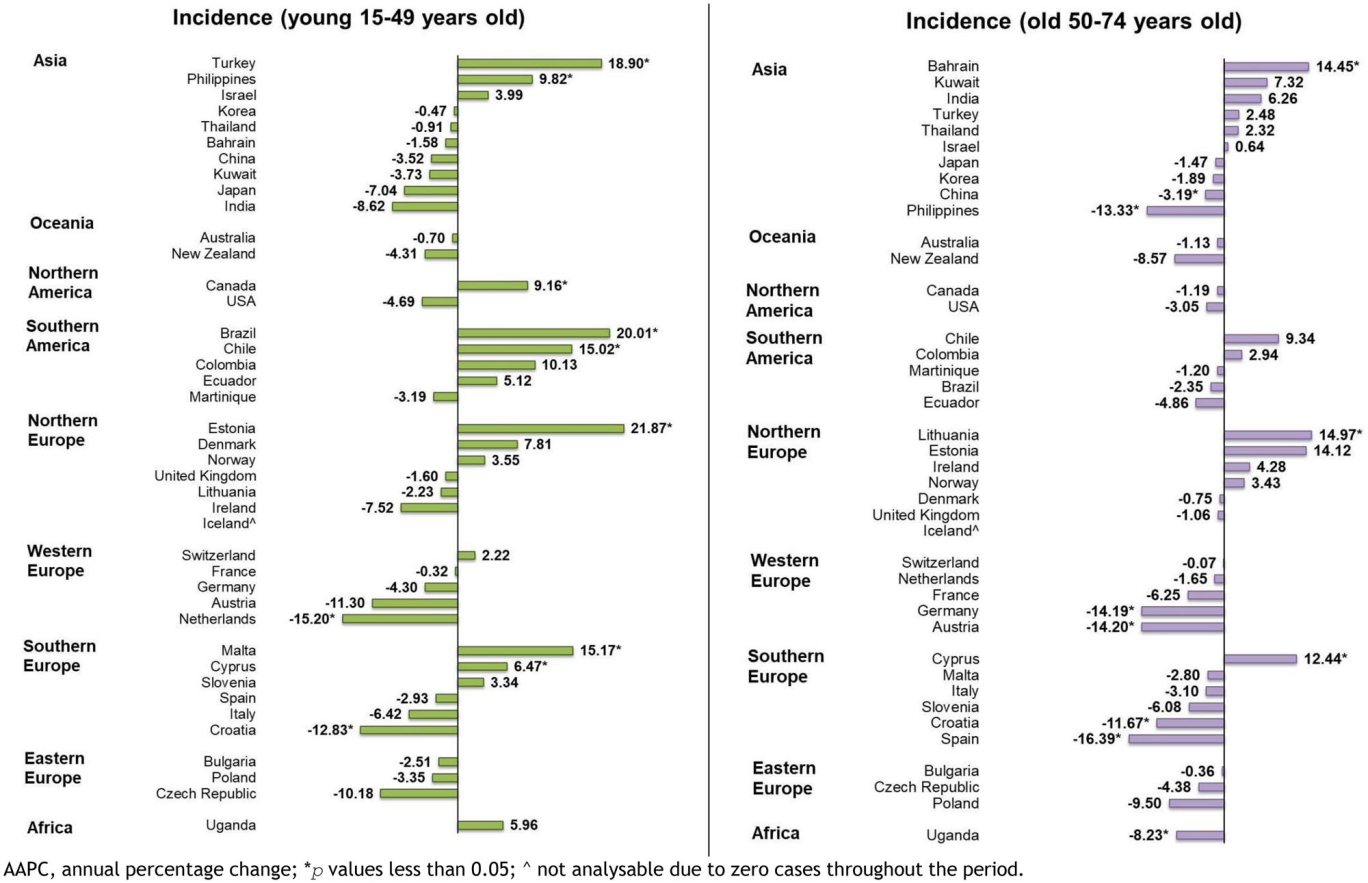
AAPC of malignant meningioma incidence by age group, both sexes.

Discrepancies were noted between the two sexes for incidence (Figure 5, Figure 6). Remarkably, decreasing rates of male malignant meningioma incidence was found among seven countries, while two countries showed on the contrary had an upward trend. Rest of the 32 countries showed no significant change over time. The most significant drop was found in Austria (AAPC: ‐16.75, 95% CI [‐23.16 to ‐9.79], *p* = 0.001), followed by Iceland (AAPC: ‐13.17, 95% CI [‐24.36 to ‐0.34], *p* = 0.045), the Philippines (AAPC: ‐13.17, 95% CI [‐23.00 to ‐2.09], *p* = 0.021), Croatia (AAPC: ‐11.86, 95% CI [‐20.50 to ‐2.28], *p* = 0.022), Poland (AAPC: ‐10.95, 95% CI [‐20.16 to ‐0.69], *p* = 0.040), the United States (AAPC: ‐7.47, 95% CI [‐13.36 to ‐1.17], *p* = 0.026), and China (AAPC: ‐3.70, 95% CI [‐7.25 to ‐0.02], *p* = 0.049). Meanwhile, India (AAPC: 16.44, 95% CI [4.56 to 29.66], *p* = 0.011) and Bulgaria (AAPC: 7.34, 95% CI [2.50 to 12.41], *p* = 0.008) were the only two countries observed with significant upward trends.

In contrast, a mixed trend was found among females, with three countries showing increasing trends and three countries showing decreasing trends. The other 35 countries did not report significant changes during this period. Estonia (AAPC: 23.48, 95% CI [10.99 to 37.37], *p* = 0.002), Lithuania (AAPC: 18.08, 95% CI [6.66 to 30.73], *p* = 0.005), and Bahrain (AAPC: 7.34, 95% CI [1.69 to 5.49], *p* < 0.001) were observed with significant increasing incidence trends. On the contrary, Croatia (AAPC: ‐11.33, 95% CI [‐16.98 to ‐5.31], *p* = 0.003), Iceland (AAPC: ‐11.32, 95% CI [‐21.26 to ‐0.13], *p* = 0.048), and Malta (AAPC: ‐1.97, 95% CI [‐2.85 to ‐1.09], *p* < 0.001) were observed with significant decreasing trends in malignant meningioma.

For the younger population, increasing incidence trends of malignant meningioma were observed in eight countries (Supplementary Figure S1, Supplementary Figure S2). The most remarkable rising trend was found in Estonia (AAPC: 21.87, 95% CI [14.41 to 29.8], *p* < 0.001), followed by Brazil (AAPC: 20.01, 95% CI [13.09 to 27.36], *p* < 0.001), Turkey (AAPC: 18.90, 95% CI [3.66 to 36.38], *p* = 0.002), Malta (AAPC: 15.17, 95% CI [0.34 to 32.20], *p* = 0.045), Chile (AAPC: 15.02, 95% CI [11.53 to 18.62], *p* < 0.001), the Philippines (AAPC: 9.82, 95% CI [2.03 to 18.20], *p* = 0.019), Canada (AAPC: 9.16, 95% CI [2.42 to 16.34], *p* = 0.013), and Cyprus (AAPC: 6.47, 95% CI [1.21 to 11.99], *p* = 0.015). Meanwhile, significant decreasing trends were reported in the Netherlands (AAPC: ‐15.20, 95% CI [‐26.34 to ‐2.39], *p* = 0.027) and Croatia (AAPC: ‐12.83, 95% CI [‐22.23 to ‐2.30], *p* = 0.024). The other 30 countries presented no significant change during this period.

For the older population, significant decreasing incidence trends could be observed in seven countries. Spain (AAPC: ‐16.39, 95% CI [‐22.75, ‐9.51], *p*<0.001) was found to have the most significant dropping trend, followed by Austria (AAPC: ‐14.20, 95% CI [‐22.71, ‐4.74], *p* = 0.010), Germany (AAPC: ‐14.19, 95% CI [‐21.93, ‐5.69], *p* = 0·006), the Philippines (AAPC: ‐13.33, 95% CI [‐19.65, ‐6.52], *p* = 0.002), Croatia (AAPC: ‐11.67, 95% CI [‐21.56, ‐0·54], *p* = 0.040), Uganda (AAPC: ‐8.23, 95% CI [‐11.23, ‐5.13], p < 0.001), and China (AAPC: ‐3.19, 95% CI [‐6.28, ‐0.00], *p* = 0.005). On the other hand, three countries, Lithuania (AAPC: 14.97, 95% CI [7.76, 22.66], *p* = 0.001), Bahrain (AAPC: 14.45, 95% CI [2.56, 27.72], *p* = 0.022), and Cyprus (AAPC: 12.44, 95% CI [8.69, 16.32], p < 0.001), were found to have significant increasing incidence trends of malignant meningioma.

## 4 Discussion

This study extensively investigated and revealed important insights into malignant meningioma burden, risk factors, and temporal trends by subgroups. A considerable national disparity exists in the burden of malignant meningioma, with the ASR for Latvia being 345 times higher than that of Fiji. Globally, malignant meningioma incidence is higher among women and older populations, compared to males and younger populations. Increased malignant meningioma incidence is positively associated with a higher prevalence of smoking, hypertension. Although malignant meningioma incidence has declined overall among men and older ages, the escalating trend among younger populations is concerning. These findings reveal the complex dynamics of malignant meningioma involving significant sex and age disparities, highlighting the need for further evidence‐based, targeted interventions.

North Africa had the highest malignant meningioma disease burden overall, consistent across both sexes, interpreting ASR requires comprehensive contextual evaluation of the region or population, but the reasons for this trend remain uncertain due to the lack of epidemiological studies in this geographic region. Our results evaluated the relationship between high HDI and increased incidence. The contradicted results may be caused by multiple risk factors have contributed to the increase in incidence of malignant meningioma, for instance, over 80% of smokers live in low‐ and middle‐income countries, which also partially explains why North Africa countries had higher incidence despite having lower HDI. Furthermore, our results revealed HDI was the risk factor for malignant cancer only in females but not other subgroups. Among the same group of high ASR countries, South America ranks much lower than East Asia or Central and Western Europe interestingly, it may suggest that South America is undergoing a demographic shift (Bedoya et al. 2006).

Health system improvements such as public health resources and diagnostic capacity may also be attributed to the high ASR observed in East Asia and Central and Western Europe (Ahmed et al. 2019). Diagnosing malignant meningioma and CNS cancers requires specialized expertise and expensive imaging modalities. Paradoxically, high‐income countries are able to afford technological advancements have higher ASR estimates owing to improved accessibility. East Asia was among the top ASR regions, foreseen by the considerable rise in the aging population (United Nations Population Division 2019), as studies in China relay a positive association between aging and meningioma incidence (Zhao et al. 2018).

Due to variations in exposure and lifestyle risks related to meningioma, it is challenging to establish definitive links and explanations for trends observed across nations. Insufficient data (Institute for Health Metrics and Evaluation (IHME) 2019), generalizations grouping all types of CNS neoplasms (Institute for Health Metrics and Evaluation (IHME) 2019), and discrepancies in identification methods and criteria by individual physicians/specialists further complicate the issue. (Naslund et al. 2023) Recent works question whether tumor control should remain the primary outcome measure for patients, emphasizing the importance of considering neurological and cognitive function, quality of life, and associated health‐related parameters to reduce over diagnosis and conserve resources, particularly in the case of incidental benign meningioma's (Naslund et al. 2023, Degeneffe et al. 2023).

Our findings contradict most literature reporting a general increase in CNS cancers (Huang et al. 2023, Ilic and Ilic 2023), as we observe a distinct pattern for females in meningioma incidence only seen in a few studies so far (Krampla et al. 2004). This emphasizes the importance of conducting specific research on meningiomas, enhancing screening efforts, and implementing customized health policies. Epidemiological and etiological studies consistently indicate that women, who produce higher levels of hormones than men, have a higher likelihood of developing meningiomas, particularly in older age groups. (Ogasawara et al. 2021, Naslund et al. 2023, Krampla et al. 2004, Lee et al. 2006, Degeneffe et al. 2023). A previous study also found associations between hypertension and meningioma have only been demonstrated in women over 60 years of age (Schneider et al. 2005). In addition, studies suggest that some genes may play a role in the incidence of meningioma's and affecting the incidence in a specific group, such as Cowden syndrome, and neurofibromatosis type 2 (NF2) (Whittle et al. 2004, Yakubov et al. 2016). NF2 may contribute to a higher incidence of meningioma's, with NF2 inactivation present in 40% to 60% of sporadic meningioma cases (Kerr et al. 2018). Higher prevalence and recurrence in older age groups (Smith et al. 2011, Dullea et al. 2023). And increased risk of meningioma's in females was found (Smith et al. 2011). These evidence are similar to those of this study and further validate the credibility of this study's findings and emphasize the role of genes in meningioma incidence, especially in certain specific groups. Future studies could further analyze the potential relationship between the prevalence of different genetic and meningioma incidence.

Positive associations of malignant meningioma incidence were observed with smoking addiction, hypertension higher HDI in females, and obesity in the older population. Regions with higher HDI values may experience increased exposure to genetic mutations or damage, such as free radiation, electromagnetic waves, nitrogenous sulphide‐containing dyes, pesticides, organ solvents, and certain viruses. (Boone et al. 2014) Improved medical conditions and disease detection in high HDI areas could also expound the results, with a study further suggesting long‐term or delayed radiation side effects on neural tissue could lead to the development of new central nervous system tumors (Ogasawara et al. 2021, Boone et al. 2014).

Exposure to N‐nitroso compounds found in tobacco is a major link to brain tumor development, but depicts uncertain associations with meningioma incidence. (Fan et al. 2013) We found a significant association between meningioma's and higher smoking prevalence in men (*β* = 0.046, 95% CI 0.032 to 0.061, p < 0.001), consistent with a meta‐analysis (Fan et al. 2013). Literature confirms our findings on obesity associated with increased meningioma risk (Shao et al. 2014), while associations amid smoking addiction, hypertension, lipid disorders, and meningioma's remain unclear. However, our results also countered previous works by reporting a positive association between meningioma incidence and higher rates of obesity, hypertension, and lipid disorders, in both males and females (Naslund et al. 2023, Huang et al. 2023, Schneider et al. 2005, Shao et al. 2014). There is an increased risk of meningioma in women with breast cancer and a higher risk of breast cancer in women diagnosed with malignant meningioma's (Lee et al. 2006). This suggests a complex relationship between these two types of cancer in women.

### 4.1 Strengths and Limitations

Malignant meningioma presents distinct disease burdens, risk factors, and epidemiological trends that set it apart from other types of meningioma. This study aims to address this knowledge gap by leveraging high‐quality data from cancer registries spanning 185 countries, facilitating a comprehensive analysis of the global incidence, associated risk factors, and temporal trends specific to malignant meningioma. By utilizing large‐scale and up‐to‐date data, the study provides detailed estimates that advance our understanding of the unique epidemiology of malignant meningioma. This targeted approach enables the identification of specific risk factors and trends, offering valuable insights that can guide the development of more effective prevention and treatment strategies tailored to malignant meningioma, ultimately contributing to improved public health outcomes. In addition, this study serves as an exploratory analysis, focusing on country‐level factors, aiming to identify potential associations that could provide insights into the disease's epidemiology by country‐level factors.

We acknowledge certain limitations which may impact data interpretations. Differences in study design are attributable to these discrepancies, as our study employed an ecological design in assessing risk factors at a population level, while previous studies may have utilized data from a single region. Firstly, the databases utilized may have inherent limitations regarding comprehensiveness and quality. Particularly frequent for underdeveloped and lower‐ to middle‐income countries (LMICs), data collection and reporting may be limited, hindered by socioeconomic and medical factors, for example, in Latvia and Fiji, due to data unavailability, their disease trends cannot be analyzed and observed. Secondly, residual confounding factors might affect the accuracy of the associations observed with risk factors. Linear regression analysis, typically employed in studies, may not fully capture potential nonlinear associations between risk factors and malignant meningioma's. Lastly, comparisons between countries or regions may not be entirely accurate due to cancer registries or the country implementing potential changes in data collection methods, inclusion criteria, and overall structure of cancer registries over time. It is crucial to compare country‐ and site‐specific results over the same period of time to ensure consistency and reliable analysis.

Overall, this study revealed a wide variation in the burden of malignant meningioma by age, sex, and geographical region. Female malignant meningioma incidence rates presented a mixed trend, suggesting the need for future studies to analyze the associated risk factors in this population. Notably, there is a significant increase in malignant meningioma incidence among the younger population, likely due to lifestyle factors and improved disease detection. Higher stress levels among young people may contribute to unhealthy coping mechanisms, such as lack of exercise and poor dietary choices leading to weight gain, which is a known risk factor for meningioma. It is important for relevant government departments and NGOs to prioritize weight management programs and educational resources to address these concerns.

## Author Contributions

**Junjie Huang**: conceptualization, supervision, data curation, formal analysis, writing–original draft. **Lai Yim**: writing–original draft. **Apurva Sawhney**: writing–original draft. **Veeleah Lok**: writing–review and editing. **Lin Zhang**: writing–review and editing. **Xu Lin**: writing–review and editing. **Don Eliseo Lucero‐Prisno III**: writing–review and editing. **Claire Chenwen Zhong**: writing–review and editing. **Wanghong Xu**: writing–review and editing. **Zhi‐Jie Zheng**: writing–review and editing. **Mellissa Withers**: writing–review and editing. **Martin CS Wong**: writing–review and editing, conceptualization, supervision.

## Ethics Statement

This study was approved by the Survey and Behavioural Research Ethics Committee, The Chinese University of Hong Kong (No. SBRE‐20‐332). We informed consent was obtained from participants (or their parent/legal guardian/next of kin) to participate in the study.

## Conflicts of Interest

The authors declare no conflicts of interest.

### Peer Review

The peer review history for this article is available at https://publons.com/publon/10.1002/brb3.70430.

## Supporting information



Supplementary Table S1a. Global incidence of malignant meningioma by sex.
Supplementary Table S1b. Global incidence of malignant meningioma by age.
Supplementary Table S2a. Associations of risk factors with malignant meningioma incidence.
Supplementary Table S2b. Multivariable analysis on risk factors and malignant meningioma incidence.
Supplementary Table S3. Trend analysis of malignant meningioma incidence.
Supplementary Figure S1. Trend analysis of malignant meningioma.
Supplementary Figure S2. The graphs of the join point regression outputs.

## Data Availability

The data used for the analyses are available upon reasonable request from the corresponding authors. The data that support the findings of this study are not publicly available due to their containing information that is private to the study participants but are available from MCSW and CCZ.
