# Supplementary material for: Segmenting the Global Layers of Malignant Meningioma: A Population‐Based Study of Incidence, Risk Factors, and Temporal Trends
Source: Brain Behav. 2025 Mar 26;15(3):e70430. doi: 10.1002/brb3.70430 (PMC11937912; doi:10.1002/brb3.70430)
Supplement: Supplementary file 1 — Supplementary Table S1a. Global incidence of malignant meningioma by sex. Supplementary Table S1b. Global incidence of malignant meningioma by age. Supplementary Table S2a. Associations of risk factors with malignant meningioma incidence. Supplementary Table S2b. Multivariable analysis on risk factors and malignant meningioma incidence. Supplementary Table S3. Trend analysis of malignant meningioma incidence. Supplementary Figure S1. Trend analysis of malignant meningioma. Supplementary Figure S2. The graphs of the join point regression outputs. [file BRB3-15-e70430-s001.docx]

**Supplementary Material**

| **Table S1a.**  Global incidence of malignant meningioma by sex …………………………………………. | 2 |
| --- | --- |
| **Table S1b.** Global incidence of malignant meningioma by age ………………………………………… | 7 |
| **Table S2a.**    Associations of risk factors with malignant meningioma incidence ………………………... | 13 |
| **Table S2b.**    Multivariable analysis on risk factors and malignant meningioma incidence ……………... | 17 |
| **Table S3.**    Trend analysis of malignant meningioma incidence ………………………………………... | 19 |
| **Figure S1.**   Trend analysis of malignant meningioma …………………………………………………... | 29 |
| **Figure S2.**   The graphs of the join point regression output ……………………………………………... | 38 |

**Table S1a. Global incidence of malignant meningioma by sex**

| Region | Both sexes | | | Males | | | Females | | |
| --- | --- | --- | --- | --- | --- | --- | --- | --- | --- |
|  | **New cases** | **ASR** | | **New cases** | **ASR** | | **New cases** | **ASR** | |
| World | 14,832 | | 1·3 | 5,044 | | 0·93 | 9,788 | | 1·6 |
| Asia | 10,703 | | 1·5 | 3,539 | | 0·97 | 7,164 | | 1·9 |
| Eastern Asia | 9,277 | | 2·7 | 2,990 | | 1·8 | 6,287 | | 3·6 |
| China | 9,025 | | 4·6 | 2,891 | | 3·0 | 6,134 | | 6·2 |
| Japan | 129 | | 0·63 | 56 | | 0·59 | 73 | | 0·68 |
| Korea, Democratic Republic of | 74 | | 2·2 | 23 | | 1·6 | 51 | | 2·8 |
| Korea, Republic of | 44 | | 0·63 | 18 | | 0·53 | 26 | | 0·70 |
| Mongolia | 5 | | 1·6 | 2 | | 1·2 | 3 | | 2·1 |
| South-Eastern Asia | 252 | | 0·35 | 85 | | 0·21 | 167 | | 0·45 |
| Brunei Darussalam | - | | 0·51 | - | | 0·00 | 0 | | 0·92 |
| Cambodia | 9 | | 0·51 | 2 | | 0·27 | 7 | | 0·80 |
| Indonesia | 83 | | 0·31 | 28 | | 0·20 | 55 | | 0·38 |
| Lao People's Democratic Republic | 3 | | 0·41 | 1 | | 0·22 | 2 | | 0·60 |
| Malaysia | 30 | | 0·94 | 23 | | 1·4 | 7 | | 0·47 |
| Myanmar | 16 | | 0·29 | 4 | | 0·17 | 12 | | 0·42 |
| Philippines | 12 | | 0·11 | 2 | | 0·04 | 10 | | 0·19 |
| Singapore | 3 | | 0·41 | 1 | | 0·27 | 2 | | 0·49 |
| Thailand | 55 | | 0·58 | 16 | | 0·36 | 39 | | 0·77 |
| Timor-Leste | - | | 0·20 | 0 | | 0·17 | 0 | | 0·17 |
| Viet Nam | 41 | | 0·40 | 8 | | 0·16 | 33 | | 0·58 |
| South-Central Asia | 857 | | 0·37 | 357 | | 0·31 | 500 | | 0·41 |
| Afghanistan | 15 | | 0·53 | 6 | | 0·44 | 9 | | 0·64 |
| Bangladesh | 19 | | 0·13 | 8 | | 0·10 | 11 | | 0·15 |
| Bhutan | - | | 0·24 | 0 | | 0·20 | 0 | | 0·20 |
| India | 384 | | 0·29 | 173 | | 0·25 | 211 | | 0·31 |
| Iran, Islamic Republic of | 315 | | 3·7 | 120 | | 2·8 | 195 | | 4·6 |
| Kazakhstan | 11 | | 0·54 | 4 | | 0·47 | 7 | | 0·64 |
| Kyrgyzstan | 3 | | 0·60 | 1 | | 0·49 | 2 | | 0·75 |
| Maldives | - | | 0·22 | 0 | | 0·27 | - | | 0·00 |
| Nepal | 4 | | 0·15 | 2 | | 0·15 | 2 | | 0·14 |
| Pakistan | 71 | | 0·38 | 29 | | 0·31 | 42 | | 0·45 |
| Sri Lanka | 7 | | 0·26 | 3 | | 0·22 | 4 | | 0·32 |
| Tajikistan | 4 | | 0·54 | 2 | | 0·47 | 2 | | 0·60 |
| Turkmenistan | 3 | | 0·57 | 1 | | 0·48 | 2 | | 0·71 |
| Uzbekistan | 21 | | 0·62 | 8 | | 0·50 | 13 | | 0·77 |
| Western Asia | 317 | | 1·1 | 107 | | 0·78 | 210 | | 1·4 |
| Armenia | 6 | | 1·6 | 2 | | 1·4 | 4 | | 1·8 |
| Azerbaijan | 12 | | 1·0 | 4 | | 0·78 | 8 | | 1·3 |
| Bahrain | 3 | | 2·8 | 1 | | 1·8 | 2 | | 4·6 |
| Gaza Strip and West Bank | 3 | | 0·80 | 1 | | 0·62 | 2 | | 0·94 |
| Georgia | 8 | | 1·4 | 3 | | 1·1 | 5 | | 1·7 |
| Iraq | 36 | | 1·2 | 12 | | 0·86 | 24 | | 1·6 |
| Israel | 6 | | 0·62 | 2 | | 0·48 | 4 | | 0·76 |
| Jordan | 11 | | 1·3 | 6 | | 1·3 | 5 | | 1·2 |
| Kuwait | 1 | | 0·19 | 1 | | 0·26 | 0 | | 0·08 |
| Lebanon | 5 | | 0·82 | 2 | | 0·64 | 3 | | 0·94 |
| Oman | 2 | | 0·56 | 1 | | 0·44 | 1 | | 0·57 |
| Qatar | - | | 0·41 | - | | 0·00 | 0 | | 0·74 |
| Saudi Arabia | 3 | | 0·07 | 2 | | 0·09 | 1 | | 0·04 |
| Syrian Arab Republic | 15 | | 1·0 | 6 | | 0·79 | 9 | | 1·2 |
| Turkey | 187 | | 1·9 | 56 | | 1·3 | 131 | | 2·5 |
| United Arab Emirates | 2 | | 0·46 | 1 | | 0·30 | 1 | | 0·69 |
| Yemen | 17 | | 0·82 | 7 | | 0·69 | 10 | | 0·91 |
| Oceania | 24 | | 0·45 | 9 | | 0·35 | 15 | | 0·55 |
| Australia | 21 | | 0·60 | 8 | | 0·47 | 13 | | 0·71 |
| Fiji | - | | 0·02 | 0 | | 0·02 | 0 | | 0·03 |
| France, New Caledonia | - | | 0·49 | 0 | | 0·46 | 0 | | 0·47 |
| French Polynesia | - | | 0·15 | 0 | | 0·20 | - | | 0·00 |
| Guam | - | | 0·00 | - | | 0·00 | - | | 0·00 |
| New Zealand | 3 | | 0·36 | 1 | | 0·24 | 2 | | 0·48 |
| Papua New Guinea | - | | 0·12 | 0 | | 0·10 | 0 | | 0·12 |
| Samoa | - | | 0·25 | 0 | | 0·33 | - | | 0·00 |
| Solomon Islands | - | | 0·08 | 0 | | 0·06 | 0 | | 0·08 |
| Vanuatu | - | | 0·17 | 0 | | 0·13 | 0 | | 0·20 |
| Northern America | 277 | | 0·54 | 121 | | 0·49 | 156 | | 0·61 |
| Canada | 37 | | 0·70 | 19 | | 0·72 | 18 | | 0·68 |
| United States of America | 240 | | 0·53 | 102 | | 0·46 | 138 | | 0·60 |
| Latin America and the Caribbean | 682 | | 1·2 | 237 | | 0·64 | 445 | | 1·5 |
| Central America & Caribbean | 160 | | 0·82 | 58 | | 0·40 | 102 | | 0·99 |
| Bahamas | - | | 0·47 | 0 | | 0·20 | 0 | | 0·52 |
| Barbados | - | | 0·35 | 0 | | 0·31 | 0 | | 0·56 |
| Belize | - | | 0·31 | 0 | | 0·37 | 0 | | 0·24 |
| Costa Rica | 2 | | 0·40 | 1 | | 1·5 | 1 | | 0·41 |
| Cuba | 3 | | 1·7 | 1 | | 0·68 | 2 | | 1·9 |
| Dominican Republic | 10 | | 0·88 | 4 | | 0·95 | 6 | | 1·1 |
| El Salvador | 10 | | 1·3 | 3 | | 0·51 | 7 | | 1·7 |
| France, Guadeloupe | - | | 0·85 | 0 | | 0·35 | 0 | | 1·3 |
| France, Martinique | - | | 0·13 | 0 | | 0·35 | - | | 0·00 |
| Guatemala | 7 | | 0·47 | 2 | | 0·06 | 5 | | 0·65 |
| Haiti | 1 | | 0·10 | 0 | | 1·0 | 1 | | 0·17 |
| Honduras | 11 | | 1·2 | 4 | | 0·00 | 7 | | 1·4 |
| Jamaica | - | | 0·00 | - | | 0·62 | - | | 0·00 |
| Mexico | 102 | | 0·79 | 38 | | 0·66 | 64 | | 0·95 |
| Nicaragua | 5 | | 0·79 | 2 | | 0·37 | 3 | | 0·95 |
| Panama | 3 | | 0·54 | 1 | | 1·1 | 2 | | 0·73 |
| Puerto Rico | 6 | | 1·4 | 2 | | 0·00 | 4 | | 1·6 |
| Saint Lucia | - | | 0·00 | - | | 0·51 | - | | 0·00 |
| Trinidad and Tobago | - | | 0·60 | 0 | | 0·64 | 0 | | 0·60 |
| South America | 522 | | 1·3 | 179 | | 0·93 | 343 | | 1·7 |
| Argentina | 143 | | 2·6 | 42 | | 1·7 | 101 | | 3·4 |
| Bolivia, Plurinational State of | 8 | | 0·73 | 2 | | 0·43 | 6 | | 1·0 |
| Brazil | 192 | | 0·75 | 72 | | 0·61 | 120 | | 0·87 |
| Chile | 29 | | 1·2 | 7 | | 0·66 | 22 | | 1·63 |
| Colombia | 69 | | 1·3 | 27 | | 1·0 | 42 | | 1·4 |
| Ecuador | 11 | | 0·58 | 4 | | 0·46 | 7 | | 0·70 |
| French Guiana | - | | 0·45 | - | | 0·00 | 0 | | 1·4 |
| Guyana | - | | 0·04 | - | | 0·00 | 0 | | 0·10 |
| Paraguay | 5 | | 0·77 | 2 | | 0·56 | 3 | | 0·90 |
| Peru | 31 | | 0·89 | 12 | | 0·66 | 19 | | 1·0 |
| Suriname | 1 | | 1·6 | 0 | | 1·2 | 1 | | 1·8 |
| Uruguay | 2 | | 0·41 | 1 | | 0·28 | 1 | | 0·48 |
| Venezuela, Bolivarian Republic of | 31 | | 1·0 | 10 | | 0·70 | 21 | | 1·3 |
| Europe | 1,592 | | 1·2 | 558 | | 0·98 | 1,034 | | 1·3 |
| Northern Europe | 135 | | 0·68 | 49 | | 0·52 | 86 | | 0·84 |
| Denmark | 2 | | 0·20 | 1 | | 0·19 | 1 | | 0·20 |
| Estonia | 1 | | 0·42 | 1 | | 0·67 | 0 | | 0·25 |
| Finland | 6 | | 0·59 | 2 | | 0·46 | 4 | | 0·74 |
| Iceland | - | | 0·00 | - | | 0·00 | - | | 0·00 |
| Ireland | 8 | | 1·1 | 3 | | 0·91 | 5 | | 1·2 |
| Latvia | 21 | | 6·9 | 5 | | 4·0 | 16 | | 9·1 |
| Lithuania | 18 | | 3·9 | 6 | | 2·9 | 12 | | 4·3 |
| Norway | 3 | | 0·44 | 2 | | 0·54 | 1 | | 0·33 |
| Sweden | 10 | | 0·63 | 4 | | 0·47 | 6 | | 0·77 |
| United Kingdom | 66 | | 0·61 | 25 | | 0·47 | 41 | | 0·76 |
| Western Europe | 211 | | 0·68 | 80 | | 0·55 | 131 | | 0·81 |
| Austria | 17 | | 1·2 | 5 | | 0·70 | 12 | | 1·7 |
| Belgium | 9 | | 0·54 | 5 | | 0·56 | 4 | | 0·49 |
| France | 72 | | 0·67 | 26 | | 0·53 | 46 | | 0·79 |
| Germany | 95 | | 0·68 | 37 | | 0·56 | 58 | | 0·79 |
| Luxembourg | - | | 0·46 | 0 | | 0·41 | 0 | | 0·45 |
| Switzerland | 10 | | 0·72 | 3 | | 0·44 | 7 | | 0·96 |
| The Netherlands | 8 | | 0·28 | 4 | | 0·27 | 4 | | 0·29 |
| Southern Europe | 290 | | 1·1 | 123 | | 0·97 | 167 | | 1·1 |
| Albania | 4 | | 1·0 | 2 | | 1·0 | 2 | | 0·93 |
| Bosnia and Herzegovina | 8 | | 1·4 | 3 | | 1·2 | 5 | | 1·5 |
| Croatia | 23 | | 1·4 | 10 | | 1·2 | 13 | | 1·6 |
| Cyprus | 9 | | 0·51 | 4 | | 0·46 | 5 | | 0·54 |
| Greece | 26 | | 1·3 | 10 | | 1·1 | 16 | | 1·4 |
| Italy | 97 | | 0·97 | 36 | | 0·82 | 61 | | 1·0 |
| Malta | 1 | | 0·89 | 0 | | 0·28 | 1 | | 1·6 |
| Montenegro | - | | 0·95 | 0 | | 0·87 | 0 | | 0·98 |
| North Macedonia | 12 | | 3·7 | 8 | | 5·0 | 4 | | 2·5 |
| Portugal | 20 | | 1·1 | 9 | | 1·1 | 11 | | 1·0 |
| Serbia | 18 | | 1·3 | 7 | | 1·2 | 11 | | 1·5 |
| Slovenia | 3 | | 0·86 | 2 | | 1·2 | 1 | | 0·58 |
| Spain | 69 | | 0·87 | 32 | | 0·86 | 37 | | 0·87 |
| Central and Eastern Europe | 956 | | 1·8 | 306 | | 1·5 | 650 | | 2·0 |
| Belarus | 26 | | 2·1 | 8 | | 1·5 | 18 | | 2·7 |
| Bulgaria | 30 | | 2·5 | 14 | | 2·5 | 16 | | 2·5 |
| Czechia | 4 | | 0·25 | 2 | | 0·21 | 2 | | 0·32 |
| Hungary | 25 | | 1·7 | 10 | | 1·5 | 15 | | 1·8 |
| Poland | 113 | | 1·9 | 39 | | 1·4 | 74 | | 2·2 |
| Republic of Moldova | 10 | | 1·9 | 4 | | 1·6 | 6 | | 2·2 |
| Romania | 60 | | 1·8 | 24 | | 1·6 | 36 | | 1·9 |
| Russian Federation | 598 | | 2·9 | 169 | | 1·9 | 429 | | 3·6 |
| Slovakia | 12 | | 1·4 | 5 | | 1·3 | 7 | | 1·4 |
| Ukraine | 78 | | 1·4 | 31 | | 1·2 | 47 | | 1·5 |
| Africa | 1,554 | | 1·6 | 580 | | 1·2 | 974 | | 2·0 |
| Northern Africa | 1,038 | | 4·7 | 354 | | 3·2 | 684 | | 6·0 |
| Algeria | 206 | | 4·8 | 67 | | 3·1 | 139 | | 6·5 |
| Egypt | 520 | | 5·8 | 172 | | 3·9 | 348 | | 7·5 |
| Libya | 26 | | 4·6 | 11 | | 3·5 | 15 | | 4·6 |
| Morocco | 176 | | 4·8 | 68 | | 3·6 | 108 | | 5·3 |
| Sudan | 56 | | 1·8 | 16 | | 1·2 | 40 | | 2·4 |
| Tunisia | 54 | | 4·1 | 20 | | 3·0 | 34 | | 5·0 |
| Sub-Saharan Africa | 516 | | 0·64 | 226 | | 0·46 | 290 | | 0·75 |
| Angola | 11 | | 0·50 | 4 | | 0·37 | 7 | | 0·63 |
| Benin | 4 | | 0·75 | 2 | | 0·80 | 2 | | 0·57 |
| Botswana | - | | 0·19 | 0 | | 0·14 | 0 | | 0·24 |
| Burkina Faso | 9 | | 0·64 | 3 | | 0·49 | 6 | | 0·82 |
| Burundi | 6 | | 0·80 | 2 | | 0·57 | 4 | | 1·1 |
| Cabo Verde | 1 | | 1·9 | 0 | | 1·1 | 1 | | 2·5 |
| Cameroon | 5 | | 0·29 | 2 | | 0·23 | 3 | | 0·34 |
| Central African Republic | 1 | | 0·42 | 0 | | 0·37 | 1 | | 0·44 |
| Chad | 5 | | 0·46 | 2 | | 0·38 | 3 | | 0·50 |
| Comoros | - | | 0·00 | - | | 0·00 | - | | 0·00 |
| Congo, Democratic Republic of | 22 | | 0·36 | 8 | | 0·28 | 14 | | 0·43 |
| Congo, Republic of | - | | 0·12 | 0 | | 0·08 | 0 | | 0·16 |
| Côte d'Ivoire | 25 | | 3·7 | 10 | | 3·1 | 15 | | 3·8 |
| Djibouti | - | | 0·23 | - | | 0·00 | 0 | | 0·62 |
| Equatorial Guinea | - | | 0·22 | 0 | | 0·15 | 0 | | 0·28 |
| Eritrea | 2 | | 0·69 | 1 | | 0·53 | 1 | | 0·75 |
| Eswatini | - | | 0·33 | 0 | | 0·12 | 0 | | 0·59 |
| Ethiopia | 46 | | 0·52 | 16 | | 0·34 | 30 | | 0·68 |
| France, La Réunion | 2 | | 1·7 | 1 | | 1·3 | 1 | | 2·0 |
| Gabon | 1 | | 0·45 | 0 | | 0·27 | 1 | | 0·68 |
| Ghana | 21 | | 0·91 | 8 | | 0·68 | 13 | | 1·1 |
| Guinea | 14 | | 1·7 | 4 | | 1·1 | 10 | | 2·1 |
| Guinea-Bissau | 2 | | 1·1 | 1 | | 0·87 | 1 | | 1·3 |
| Kenya | 33 | | 0·88 | 9 | | 0·51 | 24 | | 1·3 |
| Lesotho | - | | 0·17 | 0 | | 0·06 | 0 | | 0·33 |
| Liberia | 4 | | 1·1 | 1 | | 0·80 | 3 | | 1·5 |
| Madagascar | 15 | | 0·69 | 5 | | 0·53 | 10 | | 0·89 |
| Malawi | 4 | | 0·29 | 1 | | 0·18 | 3 | | 0·42 |
| Mali | 11 | | 0·80 | 4 | | 0·57 | 7 | | 1·0 |
| Mauritania | 2 | | 0·59 | 1 | | 0·46 | 1 | | 0·68 |
| Mauritius | 2 | | 1·5 | 1 | | 1·2 | 1 | | 1·6 |
| Mozambique | 6 | | 0·18 | 1 | | 0·07 | 5 | | 0·33 |
| Namibia | 2 | | 0·75 | 1 | | 0·57 | 1 | | 0·82 |
| Niger | 4 | | 0·27 | 3 | | 0·29 | 1 | | 0·16 |
| Nigeria | 93 | | 0·59 | 38 | | 0·49 | 55 | | 0·67 |
| Rwanda | 10 | | 1·1 | 3 | | 0·68 | 7 | | 1·6 |
| Sao Tome and Principe | - | | 0·00 | - | | 0·00 | - | | 0·00 |
| Senegal | 11 | | 0·91 | 4 | | 0·68 | 7 | | 1·2 |
| Sierra Leone | 3 | | 0·59 | 1 | | 0·46 | 2 | | 0·63 |
| Somalia | 8 | | 0·69 | 3 | | 0·46 | 5 | | 0·96 |
| South Africa | 64 | | 0·64 | 64 | | 2·7 | - | | 0·00 |
| South Sudan | 6 | | 0·69 | 2 | | 0·49 | 4 | | 0·96 |
| Tanzania, United Republic of | 10 | | 0·30 | 4 | | 0·24 | 6 | | 0·33 |
| The Republic of the Gambia | - | | 0·49 | 0 | | 0·42 | 0 | | 0·50 |
| Togo | 2 | | 0·46 | 1 | | 0·42 | 1 | | 0·42 |
| Uganda | 15 | | 0·65 | 6 | | 0·60 | 9 | | 0·73 |
| Zambia | 13 | | 1·1 | 4 | | 0·83 | 9 | | 1·4 |
| Zimbabwe | 21 | | 2·0 | 5 | | 0·96 | 16 | | 2·8 |

*ASR: per 1,000,000 persons

**Table S 1b. Global incidence of malignant meningioma by age**

| Region | Young | | Old | |
| --- | --- | --- | --- | --- |
|  | **New cases** | **ASR** | **New cases** | **ASR** |
| World | 3,083 | 0·62 | 9,491 | 4·6 |
| Asia | 2,037 | 0·59 | 7,122 | 5·2 |
| Eastern Asia | 1,584 | 1·2 | 6,384 | 9·1 |
| China | 1,550 | 1·9 | 6,244 | 14·9 |
| Japan | 13 | 0·23 | 60 | 1·3 |
| Korea, Democratic Republic of | 12 | 0·83 | 50 | 8·1 |
| Korea, Republic of | 8 | 0·29 | 27 | 1·6 |
| Mongolia | 1 | 0·65 | 3 | 5·7 |
| South-Eastern Asia | 81 | 0·24 | 141 | 1·0 |
| Brunei Darussalam | 0 | 0·89 | - | 0·00 |
| Cambodia | 3 | 0·39 | 3 | 1·3 |
| Indonesia | 32 | 0·21 | 52 | 1·0 |
| Lao People's Democratic Republic | 1 | 0·32 | 1 | 1·0 |
| Malaysia | 11 | 0·63 | 14 | 2·3 |
| Myanmar | 5 | 0·17 | 7 | 0·76 |
| Philippines | 3 | 0·06 | 6 | 0·37 |
| Singapore | 1 | 0·29 | 2 | 1·0 |
| Thailand | 16 | 0·45 | 27 | 1·3 |
| Timor-Leste | 0 | 0·05 | 0 | 0·67 |
| Viet Nam | 9 | 0·16 | 29 | 1·4 |
| South-Central Asia | 281 | 0·23 | 427 | 1·0 |
| Afghanistan | 7 | 0·43 | 5 | 1·4 |
| Bangladesh | 7 | 0·08 | 9 | 0·39 |
| Bhutan | 0 | 0·05 | 0 | 0·76 |
| India | 135 | 0·18 | 183 | 0·76 |
| Iran, Islamic Republic of | 82 | 1·7 | 177 | 12·2 |
| Kazakhstan | 3 | 0·35 | 6 | 1·7 |
| Kyrgyzstan | 1 | 0·39 | 2 | 1·9 |
| Maldives | 0 | 0·17 | 0 | 0·81 |
| Nepal | 1 | 0·09 | 2 | 0·54 |
| Pakistan | 30 | 0·28 | 28 | 1·1 |
| Sri Lanka | 2 | 0·22 | 3 | 0·56 |
| Tajikistan | 2 | 0·41 | 2 | 1·4 |
| Turkmenistan | 2 | 0·49 | 1 | 1·5 |
| Uzbekistan | 9 | 0·48 | 9 | 1·8 |
| Western Asia | 91 | 0·53 | 170 | 3·8 |
| Armenia | 1 | 0·88 | 5 | 6·0 |
| Azerbaijan | 4 | 0·70 | 8 | 3·7 |
| Bahrain | 1 | 1·0 | 2 | 11·4 |
| Gaza Strip and West Bank | 1 | 0·47 | 1 | 2·6 |
| Georgia | 2 | 0·80 | 6 | 4·9 |
| Iraq | 13 | 0·70 | 17 | 4·4 |
| Israel | 1 | 0·15 | 4 | 2·1 |
| Jordan | 4 | 0·77 | 8 | 6·4 |
| Kuwait | 0 | 0·06 | 0 | 0·34 |
| Lebanon | 2 | 0·51 | 3 | 2·7 |
| Oman | 1 | 0·37 | 1 | 1·8 |
| Qatar | - | 0·00 | 1 | 2·6 |
| Saudi Arabia | 2 | 0·07 | - | 0·00 |
| Syrian Arab Republic | 6 | 0·61 | 8 | 3·5 |
| Turkey | 45 | 0·97 | 99 | 5·9 |
| United Arab Emirates | 1 | 0·17 | 1 | 1·3 |
| Yemen | 7 | 0·53 | 6 | 2·2 |
| Oceania | 4 | 0·25 | 12 | 1·3 |
| Australia | 4 | 0·35 | 11 | 1·6 |
| Fiji | 0 | 0·05 | - | 0·00 |
| France, New Caledonia | 0 | 0·18 | 0 | 1·7 |
| French Polynesia | 0 | 0·06 | 0 | 0·66 |
| Guam | - | 0·00 | - | 0·00 |
| New Zealand | 0 | 0·09 | 1 | 0·99 |
| Papua New Guinea | 0 | 0·07 | 0 | 0·32 |
| Samoa | 0 | 0·11 | 0 | 0·84 |
| Solomon Islands | 0 | 0·08 | 0 | 0·12 |
| Vanuatu | 0 | 0·18 | 0 | 0·25 |
| Northern America | 50 | 0·28 | 148 | 1·3 |
| Canada | 9 | 0·50 | 19 | 1·5 |
| United States of America | 41 | 0·26 | 129 | 1·3 |
| Latin America and the Caribbean | 193 | 0·69 | 359 | 3·7 |
| Central America & Caribbean | 56 | 0·58 | 70 | 2·2 |
| Bahamas | 0 | 0·18 | 0 | 2·3 |
| Barbados | - | 0·00 | 0 | 2·0 |
| Belize | - | 0·00 | 0 | 2·0 |
| Costa Rica | 0 | 0·16 | 2 | 1·5 |
| Cuba | 1 | 1·1 | 2 | 6·2 |
| Dominican Republic | 4 | 0·72 | 4 | 2·4 |
| El Salvador | 3 | 1·0 | 4 | 3·4 |
| France, Guadeloupe | 0 | 0·43 | 0 | 2·1 |
| France, Martinique | 0 | 0·39 | - | 0·00 |
| Guatemala | 3 | 0·34 | 3 | 1·6 |
| Haiti | 0 | 0·06 | 1 | 0·37 |
| Honduras | 6 | 1·2 | 4 | 3·3 |
| Jamaica | - | 0·00 | - | 0·00 |
| Mexico | 35 | 0·5 | 44 | 1·9 |
| Nicaragua | 2 | 0·50 | 2 | 2·3 |
| Panama | 1 | 0·43 | 1 | 1·5 |
| Puerto Rico | 1 | 1·1 | 3 | 3·5 |
| Saint Lucia | - | 0·00 | - | 0·00 |
| Trinidad and Tobago | 0 | 0·47 | 0 | 0·93 |
| South America | 137 | 0·75 | 289 | 4·3 |
| Argentina | 33 | 1·5 | 76 | 7·9 |
| Bolivia, Plurinational State of | 2 | 0·41 | 5 | 2·6 |
| Brazil | 44 | 0·37 | 110 | 2·4 |
| Chile | 7 | 0·63 | 20 | 4·2 |
| Colombia | 22 | 0·81 | 38 | 3·6 |
| Ecuador | 4 | 0·41 | 7 | 2·3 |
| French Guiana | 0 | 2·0 | - | 0·00 |
| Guyana | - | 0·00 | 0 | 0·25 |
| Paraguay | 2 | 0·58 | 2 | 2·3 |
| Peru | 13 | 0·72 | 12 | 1·9 |
| Suriname | 0 | 1·1 | 1 | 4·8 |
| Uruguay | 0 | 0·23 | 1 | 1·4 |
| Venezuela, Bolivarian Republic of | 10 | 0·68 | 17 | 3·0 |
| Europe | 279 | 0·67 | 939 | 3·2 |
| Northern Europe | 18 | 0·35 | 68 | 1·7 |
| Denmark | 0 | 0·03 | 1 | 0·62 |
| Estonia | 0 | 0·40 | 0 | 1·1 |
| Finland | 1 | 0·37 | 3 | 1·4 |
| Iceland | - | 0·00 | - | 0·00 |
| Ireland | 1 | 0·28 | 4 | 2·6 |
| Latvia | 3 | 3·1 | 15 | 25·4 |
| Lithuania | 0 | 0·22 | 10 | 11·1 |
| Norway | 1 | 0·33 | 2 | 1·0 |
| Sweden | 1 | 0·31 | 5 | 1·7 |
| United Kingdom | 11 | 0·33 | 28 | 1·4 |
| Western Europe | 38 | 0·39 | 88 | 1·5 |
| Austria | 3 | 0·60 | 10 | 3·4 |
| Belgium | 2 | 0·37 | 5 | 1·3 |
| France | 18 | 0·58 | 27 | 1·3 |
| Germany | 12 | 0·33 | 39 | 1·4 |
| Luxembourg | 0 | 0·14 | 0 | 1·5 |
| Switzerland | 2 | 0·52 | 3 | 1·1 |
| The Netherlands | 1 | 0·16 | 4 | 0·74 |
| Southern Europe | 50 | 0·59 | 135 | 2·5 |
| Albania | 1 | 0·65 | 2 | 2·3 |
| Bosnia and Herzegovina | 1 | 0·54 | 5 | 4·1 |
| Croatia | 5 | 0·95 | 12 | 3·5 |
| Cyprus | 2 | 0·27 | 4 | 1·1 |
| Greece | 3 | 0·59 | 10 | 3·0 |
| Italy | 16 | 0·59 | 39 | 1·9 |
| Malta | 0 | 0·41 | 0 | 2·4 |
| Montenegro | 0 | 0·54 | 0 | 1·7 |
| North Macedonia | 4 | 3·2 | 6 | 9·9 |
| Portugal | 3 | 0·59 | 9 | 2·5 |
| Serbia | 3 | 0·69 | 10 | 3·4 |
| Slovenia | 1 | 0·49 | 2 | 2·3 |
| Spain | 11 | 0·44 | 36 | 2·4 |
| Central and Eastern Europe | 173 | 0·98 | 648 | 5·3 |
| Belarus | 6 | 1·2 | 17 | 5·9 |
| Bulgaria | 4 | 1·0 | 18 | 7·6 |
| Czechia | 0 | 0·04 | 2 | 0·76 |
| Hungary | 5 | 0·93 | 15 | 4·7 |
| Poland | 23 | 1·1 | 72 | 5·8 |
| Republic of Moldova | 2 | 1·1 | 7 | 5·9 |
| Romania | 10 | 0·96 | 39 | 6·2 |
| Russian Federation | 98 | 1·3 | 423 | 9·5 |
| Slovakia | 1 | 0·29 | 7 | 4·0 |
| Ukraine | 24 | 1·0 | 48 | 3·7 |
| Africa | 520 | 0·89 | 911 | 6·5 |
| Northern Africa | 348 | 2·9 | 617 | 17·0 |
| Algeria | 75 | 3·2 | 117 | 16·7 |
| Egypt | 180 | 3·6 | 285 | 19·7 |
| Libya | 11 | 2·7 | 17 | 18·3 |
| Morocco | 48 | 2·6 | 133 | 18·7 |
| Sudan | 20 | 1·1 | 25 | 5·7 |
| Tunisia | 14 | 2·2 | 40 | 15·6 |
| Sub-Saharan Africa | 172 | 0·39 | 294 | 2·5 |
| Angola | 5 | 0·39 | 4 | 1·7 |
| Benin | 2 | 0·40 | 4 | 4·2 |
| Botswana | 0 | 0·20 | - | 0·00 |
| Burkina Faso | 4 | 0·42 | 5 | 3·3 |
| Burundi | 2 | 0·52 | 3 | 3·2 |
| Cabo Verde | 0 | 1·3 | 0 | 3·5 |
| Cameroon | 3 | 0·33 | 2 | 0·81 |
| Central African Republic | 1 | 0·31 | 1 | 1·9 |
| Chad | 2 | 0·33 | 2 | 1·7 |
| Comoros | - | 0·00 | - | 0·00 |
| Congo, Democratic Republic of | 8 | 0·25 | 10 | 1·4 |
| Congo, Republic of | 0 | 0·10 | 0 | 0·46 |
| Côte d'Ivoire | 4 | 2·3 | 20 | 13·8 |
| Djibouti | 0 | 0·22 | 0 | 1·0 |
| Equatorial Guinea | 0 | 0·31 | 0 | 0·60 |
| Eritrea | 1 | 0·49 | 1 | 2·3 |
| Eswatini | 0 | 0·50 | - | 0·00 |
| Ethiopia | 18 | 0·35 | 20 | 1·9 |
| France, La Réunion | 1 | 1·3 | 2 | 6·4 |
| Gabon | 0 | 0·39 | 0 | 1·9 |
| Ghana | 9 | 0·59 | 10 | 3·0 |
| Guinea | 7 | 1·3 | 7 | 6·5 |
| Guinea-Bissau | 1 | 0·86 | 1 | 5·0 |
| Kenya | 11 | 0·42 | 18 | 4·1 |
| Lesotho | 0 | 0·25 | - | 0·00 |
| Liberia | 2 | 0·81 | 2 | 4·8 |
| Madagascar | 7 | 0·53 | 7 | 2·7 |
| Malawi | 1 | 0·19 | 1 | 0·97 |
| Mali | 5 | 0·64 | 4 | 2·9 |
| Mauritania | 1 | 0·39 | 1 | 2·7 |
| Mauritius | 1 | 1·2 | 2 | 4·4 |
| Mozambique | 1 | 0·09 | 1 | 0·57 |
| Namibia | 1 | 0·64 | 1 | 2·5 |
| Niger | 2 | 0·21 | 2 | 0·97 |
| Nigeria | 31 | 0·34 | 38 | 2·1 |
| Rwanda | 5 | 0·86 | 5 | 4·3 |
| Sao Tome and Principe | - | 0·00 | - | 0·00 |
| Senegal | 5 | 0·70 | 5 | 3·7 |
| Sierra Leone | 1 | 0·34 | 2 | 2·7 |
| Somalia | 3 | 0·42 | 4 | 2·8 |
| South Africa | - | 0·00 | 77 | 8·8 |
| South Sudan | 2 | 0·40 | 3 | 3·0 |
| Tanzania, United Republic of | 5 | 0·17 | 6 | 1·3 |
| The Republic of the Gambia | 0 | 0·32 | 0 | 2·7 |
| Togo | 1 | 0·26 | 2 | 2·5 |
| Uganda | 6 | 0·38 | 9 | 3·1 |
| Zambia | 6 | 0·81 | 5 | 3·6 |
| Zimbabwe | 7 | 1·2 | 7 | 5·8 |

*ASR: per 1,000,000 persons

**Supplementary Table 2. Associations of risk factors with malignant meningioma incidence**

| **Outcome** | **Risk factor** | **Overall** | | | |
| --- | --- | --- | --- | --- | --- |
|  |  | ***β*** | ***95% CI*** | | ***P*** |
|  | HDI | 0·125 | 0·018 | 0·232 | 0·023* |
|  | GDP per capita | -0·011 | -0·094 | 0·071 | 0·758 |
|  | Smoking | 0·058 | 0·030 | 0·086 | <0·001* |
|  | Alcohol drinking | 0·016 | -0·016 | 0·048 | 0·324 |
| **All Sexes and ages** | Dietary | 0·001 | -0·014 | 0·016 | 0·897 |
|  | Physical inactivity | 0·291 | -0·024 | 0·082 | 0·280 |
|  | Obesity | 0·227 | 0·008 | 0·037 | 0·003* |
|  | Hypertension | 0·036 | 0·017 | 0·054 | <0·001* |
|  | Diabetes | 0·015 | -0·016 | 0·046 | 0·346 |
|  | Lipid | 0·019 | 0·005 | 0·032 | 0·008* |
|  |  |  |  |  |  |
|  | HDI | 0·095 | 0·014 | 0·177 | 0·022* |
|  | GDP per capita | 0·017 | -0·045 | 0·078 | 0·597 |
|  | Smoking | 0·046 | 0·032 | 0·061 | <0·001* |
|  | Alcohol drinking | 0·018 | 0·001 | 0·035 | 0·043* |
| **Male** | Dietary | 0·008 | -0·0002 | 0·017 | 0·056 |
|  | Physical inactivity | 0·023 | -0·017 | 0·063 | 0·264 |
|  | Obesity | 0·020 | 0·009 | 0·031 | 0·001* |
|  | Hypertension | 0·024 | 0·011 | 0·038 | <0·001* |
|  | Diabetes | 0·015 | -0·007 | 0·037 | 0·172 |
|  | Lipid | 0·016 | 0·006 | 0·026 | 0·002* |
|  |  |  |  |  |  |
|  | HDI | 0·139 | 0·006 | 0·272 | 0·040* |
|  | GDP per capita | -0·036 | -0·137 | 0·066 | 0·488 |
|  | Smoking | 0·010 | -0·030 | 0·050 | 0·633 |
|  | Alcohol drinking | 0·002 | -0·053 | 0·058 | 0·932 |
| **Female** | Dietary | -0·013 | -0·035 | 0·009 | 0·229 |
|  | Physical inactivity | 0·027 | -0·035 | 0·088 | 0·393 |
|  | Obesity | 0·024 | 0·006 | 0·042 | 0·008* |
|  | Hypertension | 0·044 | 0·021 | 0·067 | <0·001* |
|  | Diabetes | 0·012 | -0·027 | 0·052 | 0·533 |
|  | Lipid | 0·020 | 0·002 | 0·037 | 0·028* |
|  |  |  |  |  |  |
|  | HDI | 0·044 | -0·016 | 0·105 | 0·147 |
|  | GDP per capita | -0·012 | -0·058 | 0·034 | 0·618 |
|  | Smoking | 0·029 | 0·011 | 0·046 | 0·002* |
|  | Alcohol drinking | -0·003 | -0·020 | 0·014 | 0·710 |
| **Young** | Dietary | 0·001 | -0·007 | 0·009 | 0·814 |
|  | Physical inactivity | 0·005 | -0·026 | 0·035 | 0·767 |
|  | Obesity | 0·009 | 0·005 | 0·017 | 0·037* |
|  | Hypertension | 0·018 | 0·002 | 0·033 | 0·025* |
|  | Diabetes | -0·002 | -0·042 | 0·038 | 0·913 |
|  | Lipid | 0·006 | -0·004 | 0·015 | 0·195 |
|  |  |  |  |  |  |
|  | HDI | 0·233 | -0·153 | 0·619 | 0·236 |
|  | GDP per capita | -0·138 | -0·430 | 0·153 | 0·350 |
|  | Smoking | 0·122 | 0·033 | 0·211 | 0·007* |
|  | Alcohol drinking | -0·049 | -0·167 | 0·068 | 0·410 |
| **Old** | Dietary | -0·033 | -0·079 | 0·014 | 0·170 |
|  | Physical inactivity | 0·119 | -0·061 | 0·299 | 0·195 |
|  | Obesity | 0·075 | 0·034 | 0·117 | <0·001* |
|  | Hypertension | 0·066 | 0·012 | 0·128 | 0·017* |
|  | Diabetes | 0·011 | -0·050 | 0·071 | 0·730 |
|  | Lipid | 0·042 | -0·008 | 0·092 | 0·098 |

The analysis was conducted using univariable linear regression model at a country level.

*β*, beta coefficient. The beta coefficient can be interpreted as the change in incidence or mortality associated

with one percent increase of a certain risk factor.

CI, confidence interval; ASR, age-standardized rate; HDI, human development index;

GDP, gross domestic products. * *P* values less than 0·05.

**Table S2b.**    Multivariable analysis on risk factors and malignant meningioma incidence

| **Outcome** | **Risk factor** | **Overall** | | | |
| --- | --- | --- | --- | --- | --- |
|  |  | ***β*** | ***95% CI*** | | ***P*** |
|  | HDI | 0.116 | -0.065 | 0.297 | 0.208 |
|  | GDP per capita | -0.112 | -0.228 | 0.005 | 0.060 |
|  | Smoking | 0.059 | 0.015 | 0.102 | **0.008*** |
|  | Alcohol drinking | -0.039 | -0.086 | 0.008 | 0.100 |
| **All Sexes and ages** | Dietary | -0.006 | -0.024 | 0.011 | 0.480 |
|  | Physical inactivity | -0.017 | -0.088 | 0.055 | 0.645 |
|  | Obesity | 0.017 | -0.004 | 0.037 | 0.113 |
|  | Hypertension | 0.031 | 0.009 | 0.053 | **0.006*** |
|  | Diabetes | -0.018 | -0.053 | 0.018 | 0.324 |
|  | Lipid | -0.002 | -0.025 | 0.022 | 0.891 |
|  |  |  |  |  |  |
|  | HDI | 0.003 | -0.131 | 0.137 | 0.960 |
|  | GDP per capita | -0.020 | -0.105 | 0.066 | 0.652 |
|  | Smoking | 0.044 | 0.024 | 0.063 | **<0·001*** |
|  | Alcohol drinking | -0.013 | -0.038 | 0.011 | 0.288 |
| **Male** | Dietary | 0.000 | -0.010 | 0.011 | 0.935 |
|  | Physical inactivity | -0.007 | -0.057 | 0.043 | 0.784 |
|  | Obesity | 0.015 | 0.000 | 0.030 | 0.051 |
|  | Hypertension | 0.014 | -0.001 | 0.029 | 0.072 |
|  | Diabetes | -0.007 | -0.032 | 0.018 | 0.558 |
|  | Lipid | -0.003 | -0.020 | 0.014 | 0.709 |
|  |  |  |  |  |  |
|  | HDI | 0.234 | 0.014 | 0.454 | **0.038*** |
|  | GDP per capita | -0.157 | -0.309 | -0.004 | **0.044*** |
|  | Smoking | -0.015 | -0.080 | 0.050 | 0.655 |
|  | Alcohol drinking | -0.045 | -0.129 | 0.038 | 0.286 |
| **Female** | Dietary | -0.006 | -0.033 | 0.020 | 0.632 |
|  | Physical inactivity | 0.006 | -0.074 | 0.086 | 0.887 |
|  | Obesity | 0.018 | -0.007 | 0.042 | 0.157 |
|  | Hypertension | 0.050 | 0.024 | 0.075 | **<0·001*** |
|  | Diabetes | -0.032 | -0.078 | 0.015 | 0.182 |
|  | Lipid | 0.009 | -0.018 | 0.036 | 0.529 |
|  |  |  |  |  |  |
|  | HDI | 0.048 | -0.057 | 0.152 | 0.369 |
|  | GDP per capita | -0.030 | -0.098 | 0.038 | 0.382 |
|  | Smoking | 0.041 | 0.014 | 0.068 | **0.003*** |
|  | Alcohol drinking | -0.028 | -0.052 | -0.004 | **0.022*** |
| **Young** | Dietary | -0.002 | -0.012 | 0.009 | 0.736 |
|  | Physical inactivity | -0.025 | -0.067 | 0.016 | 0.230 |
|  | Obesity | 0.012 | 0.000 | 0.024 | 0.059 |
|  | Hypertension | 0.013 | -0.003 | 0.030 | 0.113 |
|  | Diabetes | -0.024 | -0.071 | 0.023 | 0.308 |
|  | Lipid | -0.005 | -0.018 | 0.009 | 0.505 |
|  |  |  |  |  |  |
|  | HDI | 0.368 | -0.277 | 1.013 | 0.261 |
|  | GDP per capita | -0.317 | -0.753 | 0.119 | 0.154 |
|  | Smoking | 0.147 | 0.022 | 0.271 | **0.022*** |
|  | Alcohol drinking | -0.168 | -0.331 | -0.005 | **0.044*** |
| **Old** | Dietary | -0.033 | -0.094 | 0.027 | 0.281 |
|  | Physical inactivity | -0.093 | -0.349 | 0.162 | 0.472 |
|  | Obesity | 0.065 | 0.005 | 0.125 | **0.034*** |
|  | Hypertension | 0.054 | -0.001 | 0.109 | 0.055 |
|  | Diabetes | -0.052 | -0.122 | 0.018 | 0.146 |
|  | Lipid | 0.000 | -0.077 | 0.076 | 0.998 |

**Table S3. Trend analysis of malignant meningioma incidence**

**a. Male**

| **Region** | **AAPC** | **Lower CI** | **Upper CI** | **p-value** | **Significant** |
| --- | --- | --- | --- | --- | --- |
| ***Asia*** |  |  |  |  |  |
| Bahrain | 3·14 | -4·59 | 11·51 | 0·436 |  |
| China | -3·70 | -7·25 | -0·02 | 0·049 | * |
| India | 16·44 | 4·56 | 29·66 | 0·011 | * |
| Israel | 5·07 | -9·50 | 21·99 | 0·467 |  |
| Japan | -11·51 | -36·55 | 23·42 | 0·471 |  |
| Korea | 2·57 | -5·37 | 11·18 | 0·488 |  |
| Kuwait | 7·32 | -6·51 | 23·18 | 0·316 |  |
| Philippines | -13·17 | -23·00 | -2·09 | 0·021 | * |
| Thailand | -6·35 | -16·01 | 4·42 | 0·202 |  |
| Turkey | -0·21 | -12·75 | 14·14 | 0·973 |  |
| ***Oceania*** |  |  |  |  |  |
| Australia | -2·53 | -9·53 | 5·02 | 0·451 |  |
| New Zealand | -2·25 | -15·46 | 13·02 | 0·727 |  |
| ***Northern America*** |  |  |  |  |  |
| Canada | 4·62 | -2·30 | 12·03 | 0·166 |  |
| USA | -7·47 | -13·36 | -1·17 | 0·026 | * |
| ***Southern America*** |  |  |  |  |  |
| Brazil | -8·66 | -18·10 | 1·87 | 0·092 |  |
| Chile | -4·98 | -13·83 | 4·78 | 0·306 |  |
| Colombia | 2·05 | -8·22 | 13·48 | 0·670 |  |
| Ecuador | -9·86 | -30·65 | 17·18 | 0·438 |  |
| Martinique | 7·14 | -4·83 | 20·61 | 0·254 |  |
| ***Northern Europe*** |  |  |  |  |  |
| Denmark | -3·69 | -14·39 | 8·34 | 0·482 |  |
| Estonia | 11·04 | -0·44 | 23·85 | 0·060 |  |
| Iceland | -13·17 | -24·36 | -0·34 | 0·045 | * |
| Ireland | 11·80 | -6·39 | 33·53 | 0·186 |  |
| Lithuania | 11·38 | -4·44 | 29·83 | 0·143 |  |
| Norway | 9·86 | -5·14 | 27·23 | 0·178 |  |
| United Kingdom | 1·13 | -5·69 | 8·44 | 0·720 |  |
| ***Western Europe*** |  |  |  |  |  |
| Austria | -16·75 | -23·16 | -9·79 | 0·001 | * |
| France | -7·27 | -14·74 | 0·86 | 0·072 |  |
| Germany | -10·83 | -23·90 | 4·48 | 0·134 |  |
| Netherlands | -4·43 | -12·28 | 4·14 | 0·258 |  |
| Switzerland | -1·40 | -15·40 | 14·91 | 0·837 |  |
| ***Southern Europe*** |  |  |  |  |  |
| Croatia | -11·86 | -20·50 | -2·28 | 0·022 | * |
| Cyprus | 11·20 | -6·63 | 32·43 | 0·234 |  |
| Italy | -9·86 | -21·66 | 3·71 | 0·126 |  |
| Malta | -4·09 | -15·38 | 8·71 | 0·513 |  |
| Slovenia | -4·55 | -19·02 | 12·50 | 0·532 |  |
| Spain | 3·91 | -4·35 | 12·88 | 0·316 |  |
| ***Eastern Europe*** |  |  |  |  |  |
| Bulgaria | 7·34 | 2·50 | 12·41 | 0·008 | * |
| Czech Republic | -4·78 | -12·20 | 3·27 | 0·202 |  |
| Poland | -10·95 | -20·16 | -0·69 | 0·040 | * |
| ***Africa*** |  |  |  |  |  |
| Uganda | -8·67 | -19·25 | 3·31 | 0·128 |  |

AAPC, annual percentage change; CI, confidence interval; * p values less than 0·05.

**b. Female**

| **Region** | **AAPC** | **Lower CI** | **Upper CI** | **p-value** | **Significant** |
| --- | --- | --- | --- | --- | --- |
| ***Asia*** |  |  |  |  |  |
| Bahrain | 3·57 | 1·69 | 5·49 | <0·001 | * |
| China | -3·18 | -6·66 | 0·42 | 0·075 |  |
| India | -1·55 | -14·93 | 13·94 | 0·811 |  |
| Israel | -0·39 | -13·98 | 15·36 | 0·953 |  |
| Japan | -4·74 | -13·75 | 5·20 | 0·292 |  |
| Korea | -0·62 | -9·69 | 9·35 | 0·884 |  |
| Kuwait | -3·73 | -12·70 | 6·16 | 0·446 |  |
| Philippines | -11·76 | -25·52 | 4·53 | 0·148 |  |
| Thailand | 3·30 | -13·68 | 23·62 | 0·687 |  |
| Turkey | 9·81 | -2·88 | 24·16 | 0·117 |  |
| ***Oceania*** |  |  |  |  |  |
| Australia | 0·45 | -3·95 | 5·06 | 0·821 |  |
| New Zealand | 1·58 | -17·14 | 24·53 | 0·863 |  |
| ***Northern America*** |  |  |  |  |  |
| Canada | -0·67 | -7·88 | 7·12 | 0·843 |  |
| USA | -3·96 | -9·80 | 2·25 | 0·175 |  |
| ***Southern America*** |  |  |  |  |  |
| Brazil | -5·76 | -16·83 | 6·78 | 0·352 |  |
| Chile | 10·10 | -6·77 | 30·02 | 0·219 |  |
| Colombia | 3·88 | -8·20 | 17·56 | 0·497 |  |
| Ecuador | -3·14 | -17·49 | 13·69 | 0·658 |  |
| Martinique | 19·71 | -1·60 | 45·64 | 0·072 |  |
| ***Northern Europe*** |  |  |  |  |  |
| Denmark | -9·47 | -20·93 | 3·65 | 0·128 |  |
| Estonia | 23·48 | 10·99 | 37·37 | 0·002 | * |
| Iceland | -11·32 | -21·26 | -0·13 | 0·048 | * |
| Ireland | -3·86 | -15·13 | 8·90 | 0·487 |  |
| Lithuania | 18·08 | 6·66 | 30·73 | 0·005 | * |
| Norway | -3·44 | -21·73 | 19·13 | 0·711 |  |
| United Kingdom | -1·50 | -7·14 | 4·50 | 0·573 |  |
| ***Western Europe*** |  |  |  |  |  |
| Austria | -10·96 | -21·03 | 0·39 | 0·056 |  |
| France | 5·06 | -7·65 | 19·52 | 0·403 |  |
| Germany | -12·03 | -31·06 | 12·25 | 0·260 |  |
| Netherlands | -5·50 | -13·34 | 3·04 | 0·170 |  |
| Switzerland | -1·72 | -21·90 | 23·66 | 0·866 |  |
| ***Southern Europe*** |  |  |  |  |  |
| Croatia | -11·33 | -16·98 | -5·31 | 0·003 | * |
| Cyprus | -0·21 | -12·84 | 14·26 | 0·973 |  |
| Italy | 4·29 | -14·56 | 27·30 | 0·640 |  |
| Malta | -1·97 | -2·85 | -1·09 | <0·001 | * |
| Slovenia | -4·93 | -20·02 | 13·02 | 0·520 |  |
| Spain | -11·96 | -27·18 | 6·43 | 0·188 |  |
| ***Eastern Europe*** |  |  |  |  |  |
| Bulgaria | -4·48 | -14·54 | 6·78 | 0·420 |  |
| Czech Republic | -3·52 | -8·76 | 2·02 | 0·177 |  |
| Poland | -4·39 | -20·72 | 15·31 | 0·596 |  |
| ***Africa*** |  |  |  |  |  |
| Uganda | -3·10 | -17·46 | 13·77 | 0·663 |  |

AAPC, annual percentage change; CI, confidence interval; * p values less than 0·05.

**c. Both**

| **Region** | **AAPC** | **Lower CI** | **Upper CI** | **p-value** | **Significant** |
| --- | --- | --- | --- | --- | --- |
| ***Asia*** |  |  |  |  |  |
| Bahrain | 3·96 | -21·85 | 38·29 | 0·790 |  |
| China | -3·37 | -6·37 | -0·28 | 0·036 | * |
| India | -0·93 | -16·74 | 17·88 | 0·904 |  |
| Israel | 3·92 | -8·96 | 18·62 | 0·522 |  |
| Japan | -5·15 | -13·29 | 3·75 | 0·211 |  |
| Korea | 1·12 | -5·65 | 8·39 | 0·720 |  |
| Kuwait | 4·16 | -7·75 | 17·61 | 0·511 |  |
| Philippines | -4·00 | -16·08 | 9·81 | 0·503 |  |
| Thailand | -3·06 | -22·31 | 20·97 | 0·755 |  |
| Turkey | 5·66 | 0·41 | 11·18 | 0·038 | * |
| ***Oceania*** |  |  |  |  |  |
| Australia | -0·52 | -4·74 | 3·89 | 0·790 |  |
| New Zealand | 5·05 | -9·82 | 22·36 | 0·478 |  |
| ***Northern America*** |  |  |  |  |  |
| Canada | 1·46 | -5·18 | 8·56 | 0·636 |  |
| USA | -5·63 | -10·18 | -0·85 | 0·027 | * |
| ***Southern America*** |  |  |  |  |  |
| Brazil | -0·76 | -15·37 | 16·38 | 0·915 |  |
| Chile | 7·93 | -10·67 | 30·40 | 0·379 |  |
| Colombia | 5·21 | -6·62 | 18·54 | 0·355 |  |
| Ecuador | -2·24 | -20·73 | 20·57 | 0·810 |  |
| Martinique | 15·05 | -8·57 | 44·76 | 0·232 |  |
| ***Northern Europe*** |  |  |  |  |  |
| Denmark | -4·86 | -14·67 | 6·08 | 0·322 |  |
| Estonia | 18·61 | -1·93 | 43·47 | 0·079 |  |
| Iceland | -18·37 | -31·26 | -3·05 | 0·021 | * |
| Ireland | 1·27 | -9·84 | 13·74 | 0·809 |  |
| Lithuania | 14·16 | 8·19 | 20·47 | <0·001 | * |
| Norway | 6·33 | -9·04 | 24·29 | 0·391 |  |
| United Kingdom | -0·39 | -5·75 | 5·28 | 0·876 |  |
| ***Western Europe*** |  |  |  |  |  |
| Austria | -12·96 | -21·11 | -3·98 | 0·012 | * |
| France | -1·81 | -7·59 | 4·34 | 0·508 |  |
| Germany | -13·14 | -21·53 | -3·84 | 0·013 | * |
| Netherlands | -4·76 | -10·66 | 1·53 | 0·116 |  |
| Switzerland | 3·60 | -18·06 | 30·99 | 0·737 |  |
| ***Southern Europe*** |  |  |  |  |  |
| Croatia | -11·34 | -17·54 | -4·67 | 0·005 | * |
| Cyprus | 3·94 | -12·03 | 22·80 | 0·608 |  |
| Italy | -0·75 | -12·46 | 12·52 | 0·893 |  |
| Malta | 0·48 | -8·20 | 9·98 | 0·918 |  |
| Slovenia | -3·20 | -14·62 | 9·76 | 0·567 |  |
| Spain | -6·07 | -17·81 | 7·34 | 0·358 |  |
| ***Eastern Europe*** |  |  |  |  |  |
| Bulgaria | -0·11 | -6·78 | 7·03 | 0·974 |  |
| Czech Republic | -4·58 | -9·62 | 0·75 | 0·082 |  |
| Poland | -10·56 | -22·16 | 2·77 | 0·101 |  |
| ***Africa*** |  |  |  |  |  |
| Uganda | -5·70 | -17·97 | 8·41 | 0·360 |  |

AAPC, annual percentage change; CI, confidence interval; * p values less than 0·05.

**d. Young**

| **Region** | **AAPC** | **Lower CI** | **Upper CI** | **p-value** | **Significant** |
| --- | --- | --- | --- | --- | --- |
| ***Asia*** |  |  |  |  |  |
| Bahrain | -1·58 | -9·66 | 7·22 | 0·680 |  |
| China | -3·52 | -8·49 | 1·71 | 0·156 |  |
| India | -8·62 | -18·14 | 2·01 | 0·096 |  |
| Israel | 3·99 | -2·46 | 10·88 | 0·197 |  |
| Japan | -7·04 | -20·38 | 8·54 | 0·309 |  |
| Korea | -0·47 | -14·09 | 15·31 | 0·943 |  |
| Kuwait | -3·73 | -12·70 | 6·16 | 0·446 |  |
| Philippines | 9·82 | 2·03 | 18·20 | 0·019 | * |
| Thailand | -0·91 | -15·91 | 16·77 | 0·901 |  |
| Turkey | 18·90 | 3·66 | 36·38 | 0·020 | * |
| ***Oceania*** |  |  |  |  |  |
| Australia | -0·70 | -8·79 | 8·10 | 0·853 |  |
| New Zealand | -4·31 | -14·62 | 7·25 | 0·449 |  |
| ***Northern America*** |  |  |  |  |  |
| Canada | 9·16 | 2·42 | 16·34 | 0·013 | * |
| USA | -4·69 | -14·00 | 5·63 | 0·313 |  |
| ***Southern America*** |  |  |  |  |  |
| Brazil | 20·01 | 13·09 | 27·36 | <0·001 | * |
| Chile | 15·02 | 11·53 | 18·62 | <0·001 | * |
| Colombia | 10·13 | -6·03 | 29·06 | 0·198 |  |
| Ecuador | 5·12 | -7·72 | 19·74 | 0·403 |  |
| Martinique | -3·19 | -10·18 | 4·34 | 0·396 |  |
| ***Northern Europe*** |  |  |  |  |  |
| Denmark | 7·81 | -1·37 | 17·85 | 0·098 |  |
| Estonia | 21·87 | 14·41 | 29·83 | <0·001 | * |
| Iceland | NA | NA | NA | NA | NA |
| Ireland | -7·52 | -19·17 | 5·81 | 0·217 |  |
| Lithuania | -2·23 | -16·67 | 14·70 | 0·782 |  |
| Norway | 3·55 | -6·10 | 14·20 | 0·434 |  |
| United Kingdom | -1·60 | -8·31 | 5·60 | 0·613 |  |
| ***Western Europe*** |  |  |  |  |  |
| Austria | -11·30 | -21·50 | 0·23 | 0·054 |  |
| France | -0·32 | -14·83 | 16·66 | 0·964 |  |
| Germany | -4·30 | -13·57 | 5·97 | 0·349 |  |
| Netherlands | -15·20 | -26·34 | -2·39 | 0·027 | * |
| Switzerland | 2·22 | -6·07 | 11·23 | 0·566 |  |
| ***Southern Europe*** |  |  |  |  |  |
| Croatia | -12·83 | -22·23 | -2·30 | 0·024 | * |
| Cyprus | 6·47 | 1·21 | 11·99 | 0·015 | * |
| Italy | -6·42 | -16·38 | 4·72 | 0·211 |  |
| Malta | 15·17 | 0·34 | 32·20 | 0·045 | * |
| Slovenia | 3·34 | -5·53 | 13·04 | 0·423 |  |
| Spain | -2·93 | -14·19 | 9·81 | 0·636 |  |
| ***Eastern Europe*** |  |  |  |  |  |
| Bulgaria | -2·51 | -8·72 | 4·12 | 0·399 |  |
| Czech Republic | -10·18 | -23·44 | 5·37 | 0·160 |  |
| Poland | -3·35 | -14·02 | 8·65 | 0·521 |  |
| ***Africa*** |  |  |  |  |  |
| Uganda | 5·96 | -6·15 | 19·63 | 0·303 |  |

AAPC, annual percentage change; CI, confidence interval; * p values less than 0·05.

NA, not available as it reported zero cases during the period and joinpoint regression could not be performed in such circumstances.

**e. Old**

| **Region** | **AAPC** | **Lower CI** | **Upper CI** | **p-value** | **Significant** |
| --- | --- | --- | --- | --- | --- |
| ***Asia*** |  |  |  |  |  |
| Bahrain | 14·45 | 2·56 | 27·72 | 0·022 | * |
| China | -3·19 | -6·28 | 0·00 | 0·050 | * |
| India | 6·26 | -9·06 | 24·16 | 0·394 |  |
| Israel | 0·64 | -11·72 | 14·73 | 0·913 |  |
| Japan | -1·47 | -11·42 | 9·61 | 0·757 |  |
| Korea | -1·89 | -7·71 | 4·31 | 0·494 |  |
| Kuwait | 7·32 | -6·51 | 23·18 | 0·316 |  |
| Philippines | -13·33 | -19·65 | -6·52 | 0·002 | * |
| Thailand | 2·32 | -8·00 | 13·80 | 0·632 |  |
| Turkey | 2·48 | -6·46 | 12·29 | 0·553 |  |
| ***Oceania*** |  |  |  |  |  |
| Australia | -1·13 | -4·89 | 2·77 | 0·516 |  |
| New Zealand | -8·57 | -20·56 | 5·22 | 0·180 |  |
| ***Northern America*** |  |  |  |  |  |
| Canada | -1·19 | -9·37 | 7·73 | 0·758 |  |
| USA | -3·05 | -8·47 | 2·69 | 0·250 |  |
| ***Southern America*** |  |  |  |  |  |
| Brazil | -2·35 | -14·69 | 11·78 | 0·696 |  |
| Chile | 9·34 | -17·65 | 45·16 | 0·537 |  |
| Colombia | 2·94 | -9·98 | 17·71 | 0·632 |  |
| Ecuador | -4·86 | -15·04 | 6·55 | 0·340 |  |
| Martinique | -1·20 | -9·08 | 7·36 | 0·776 |  |
| ***Northern Europe*** |  |  |  |  |  |
| Denmark | -0·75 | -13·53 | 13·92 | 0·903 |  |
| Estonia | 14·12 | -2·55 | 33·64 | 0·101 |  |
| Iceland | NA | NA | NA | NA | NA |
| Ireland | 4·28 | -8·62 | 18·98 | 0·485 |  |
| Lithuania | 14·97 | 7·76 | 22·66 | 0·001 | * |
| Norway | 3·43 | -11·78 | 21·25 | 0·638 |  |
| United Kingdom | -1·06 | -6·56 | 4·77 | 0·680 |  |
| ***Western Europe*** |  |  |  |  |  |
| Austria | -14·20 | -22·71 | -4·74 | 0·010 | * |
| France | -6·25 | -14·96 | 3·35 | 0·165 |  |
| Germany | -14·19 | -21·93 | -5·69 | 0·006 | * |
| Netherlands | -1·65 | -9·04 | 6·35 | 0·637 |  |
| Switzerland | -0·07 | -11·06 | 12·28 | 0·989 |  |
| ***Southern Europe*** |  |  |  |  |  |
| Croatia | -11·67 | -21·56 | -0·54 | 0·040 | * |
| Cyprus | 12·44 | 8·69 | 16·32 | <0·001 | * |
| Italy | -3·10 | -16·16 | 12·00 | 0·630 |  |
| Malta | -2·80 | -10·22 | 5·24 | 0·484 |  |
| Slovenia | -6·08 | -15·18 | 3·99 | 0·193 |  |
| Spain | -16·39 | -22·75 | -9·51 | <0·001 | * |
| ***Eastern Europe*** |  |  |  |  |  |
| Bulgaria | -0·36 | -11·54 | 12·23 | 0·952 |  |
| Czech Republic | -4·38 | -10·85 | 2·56 | 0·179 |  |
| Poland | -9·50 | -19·32 | 1·51 | 0·080 |  |
| ***Africa*** |  |  |  |  |  |
| Uganda | -8·23 | -11·23 | -5·13 | <0·001 | * |

AAPC, annual percentage change; CI, confidence interval; * p values less than 0·05.

NA, not available as it reported zero cases during the period and joinpoint regression could not be performed in such circumstances.

**Figure S1. Trend analysis of malignant meningioma**

| **Asia** | | |
| --- | --- | --- |
|  |  |  |
|  |  |  |
|  |  |  |
|  |  |  |

| **Oceania** | | |
| --- | --- | --- |
|  |  |  |
| **Northern America** | | |
|  |  |  |

| **Southern America** | | |
| --- | --- | --- |
|  |  |  |
|  |  |  |

| **Northern Europe** | | |
| --- | --- | --- |
|  |  |  |
|  |  |  |
|  |  |  |
| **Western Europe** | | |
|  |  |  |
|  |  |  |

| **Southern Europe** | | |
| --- | --- | --- |
|  |  |  |
|  |  |  |

| **Eastern Europe** | | |
| --- | --- | --- |
|  |  |  |
| **Africa** | | |
|  |  |  |

**Figure S2. The graphs of the joinpoint regression output**

**a.) Male**

| **Asia** | |
| --- | --- |
| 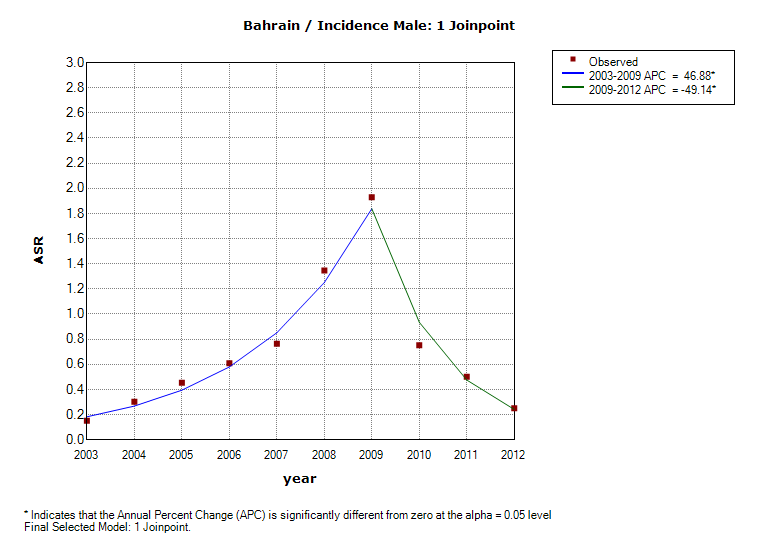 | 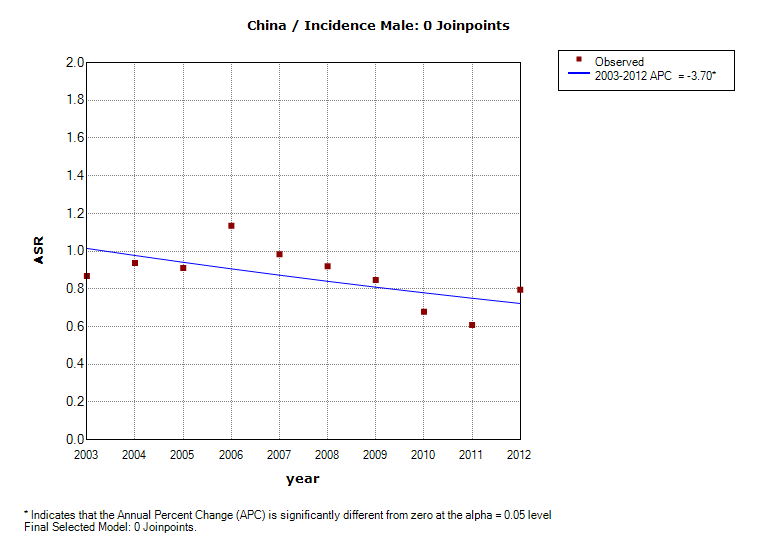 |
| 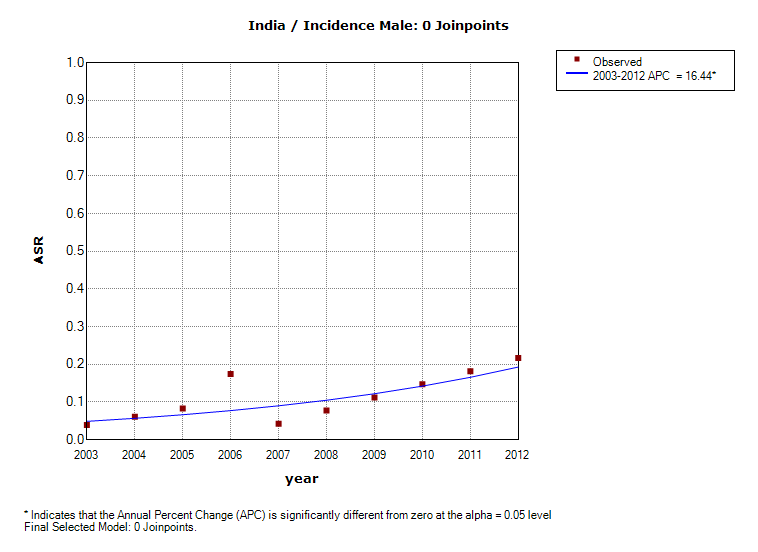 | 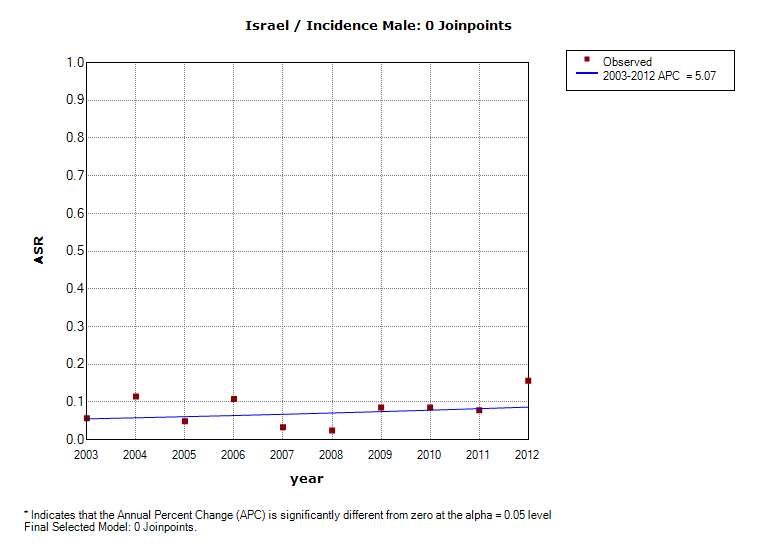 |
| 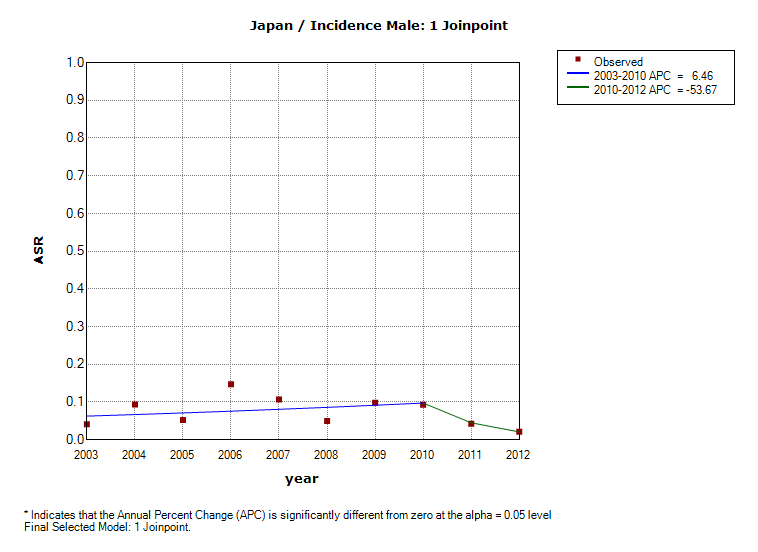 | 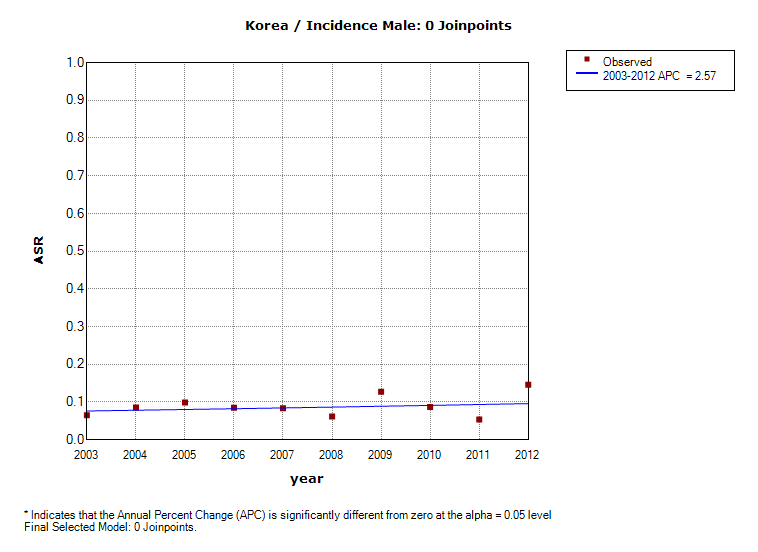 |
| 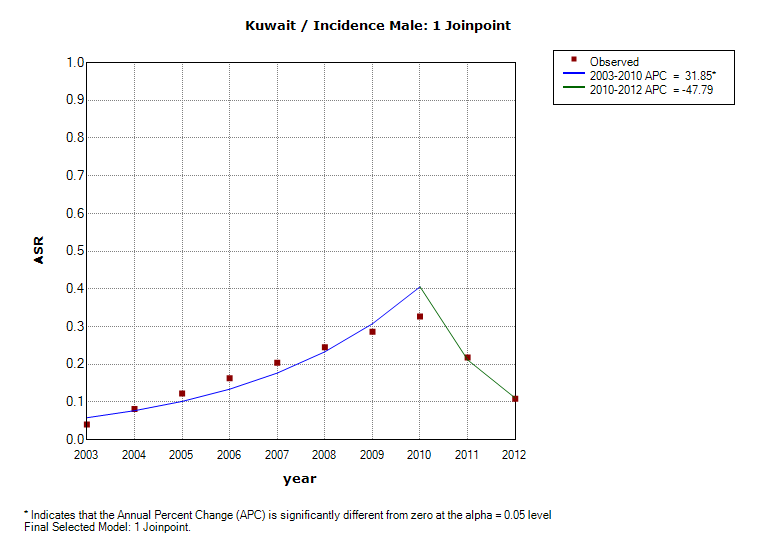 | 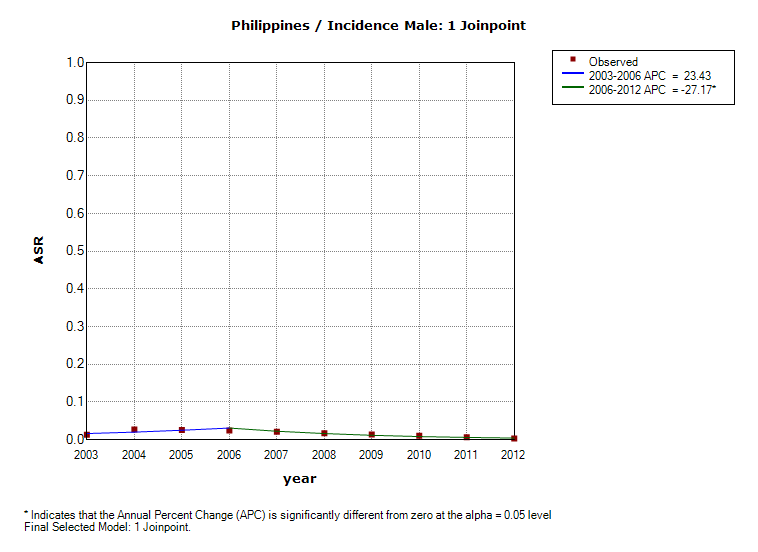 |
| 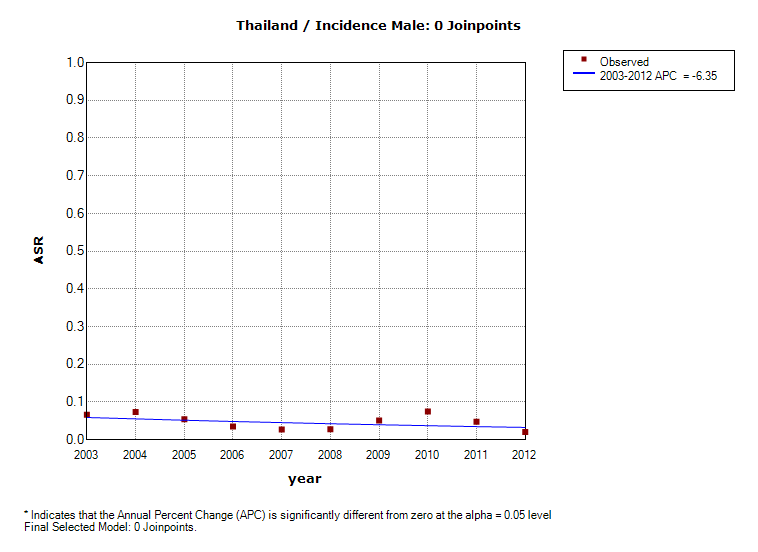 | 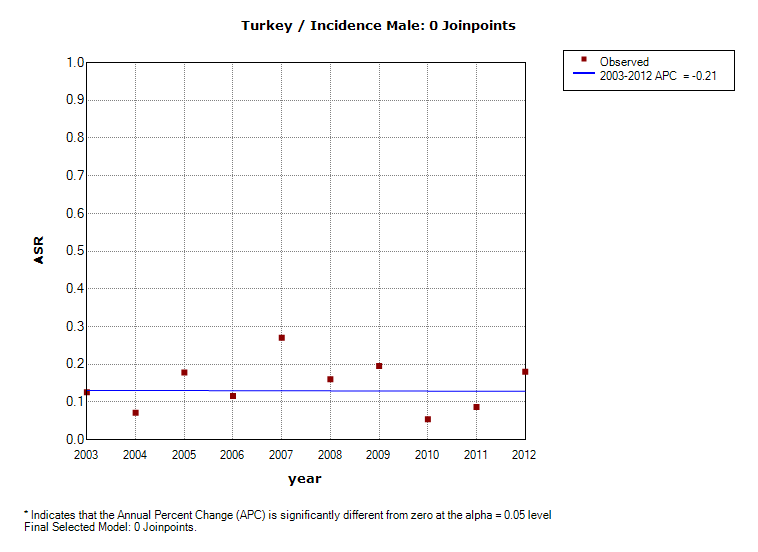 |
| **Oceania** | |
| 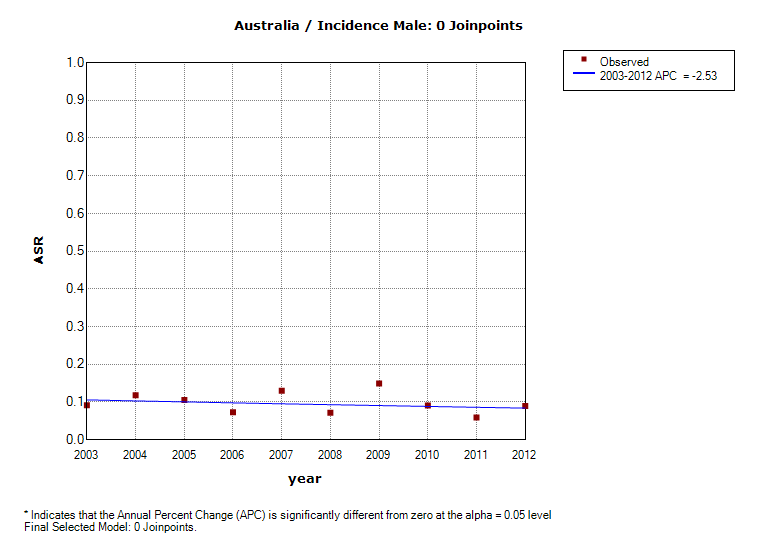 | 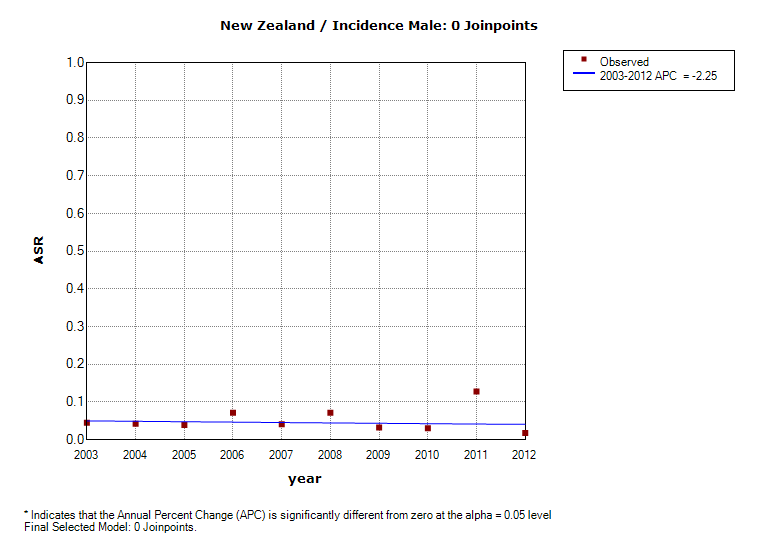 |

| **Northern America** | |
| --- | --- |
| 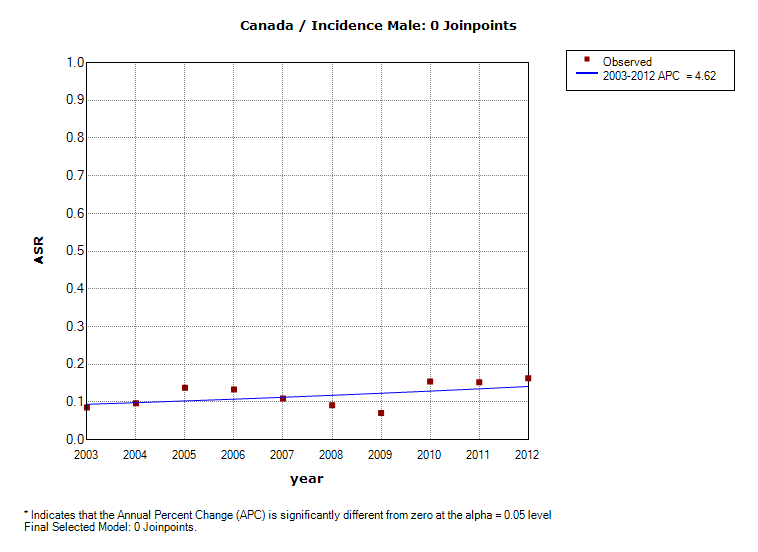 | 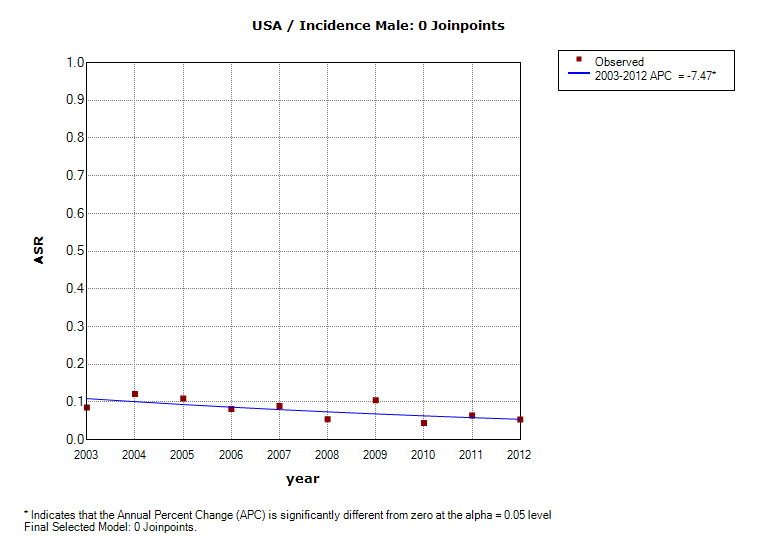 |
| **Southern America** | |
| 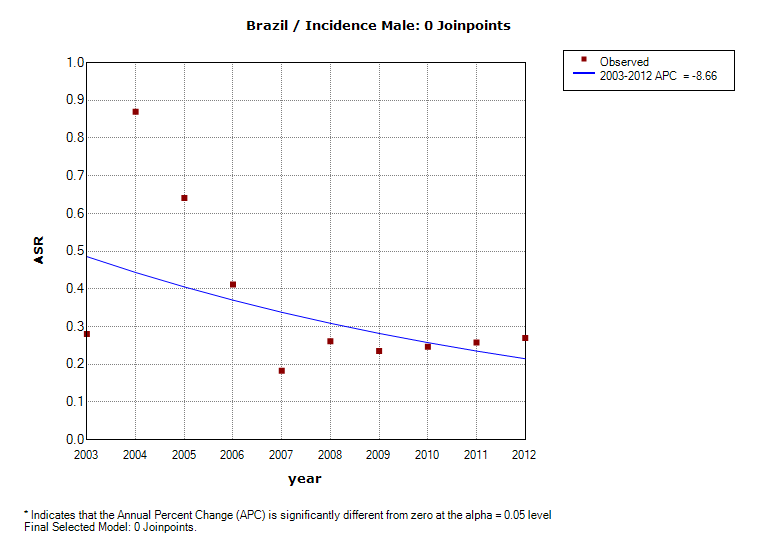 | 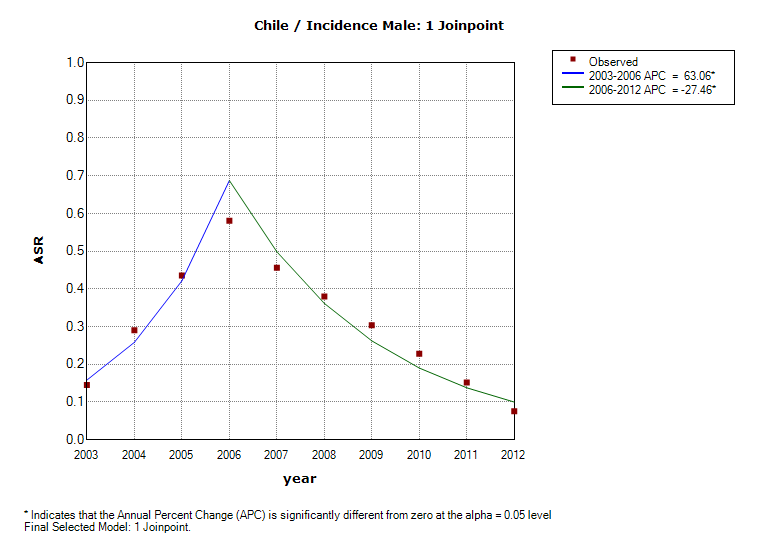 |
| 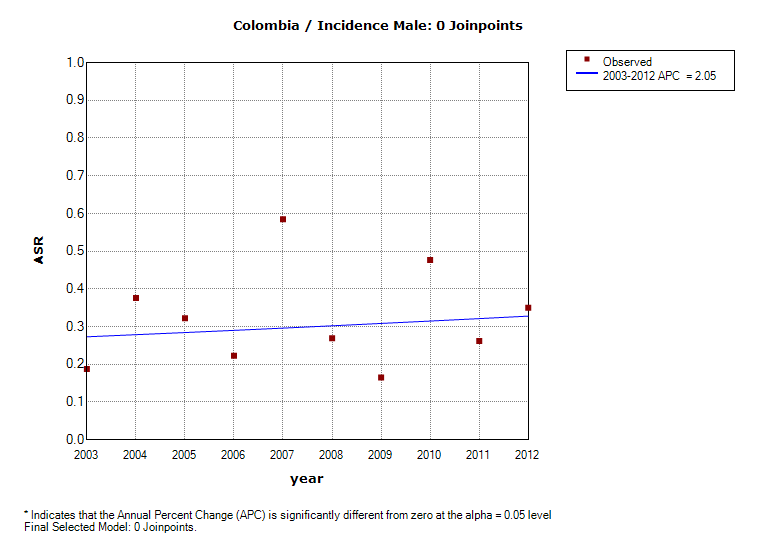 | 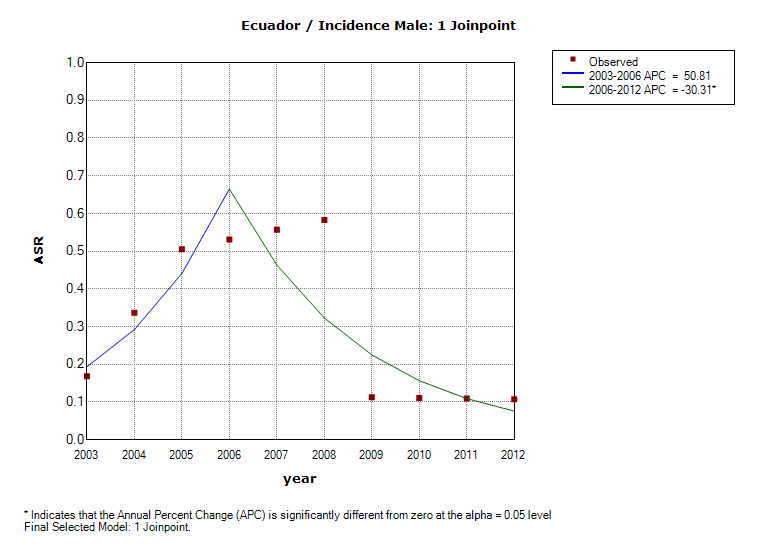 |
| 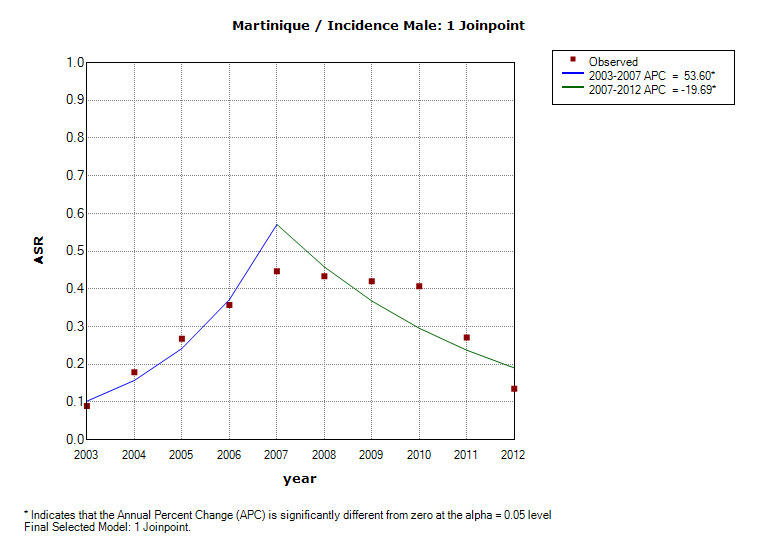 |  |

| **Northern Europe** | |
| --- | --- |
| 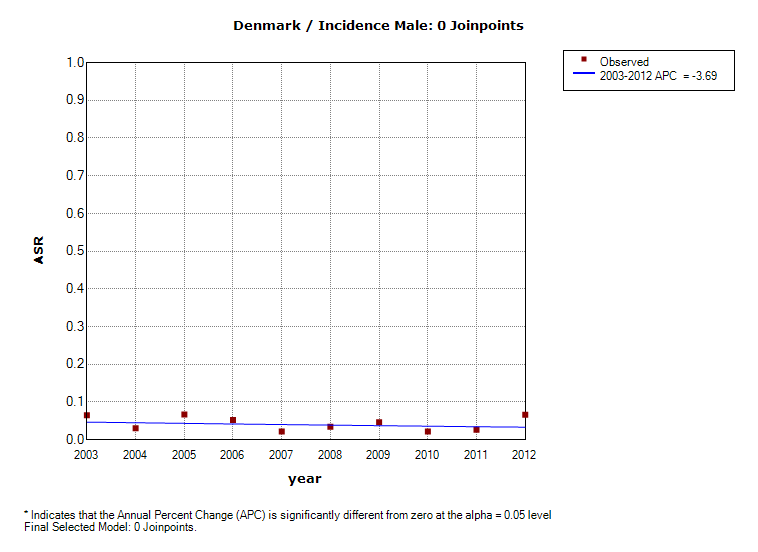 | 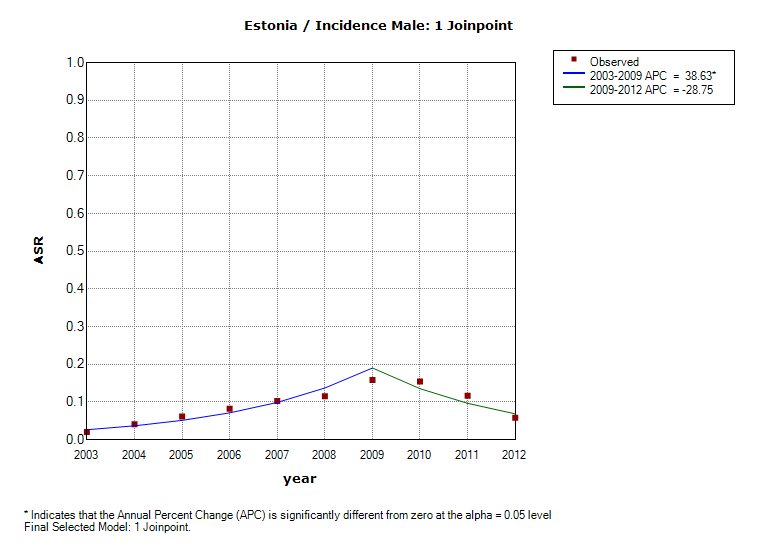 |
| 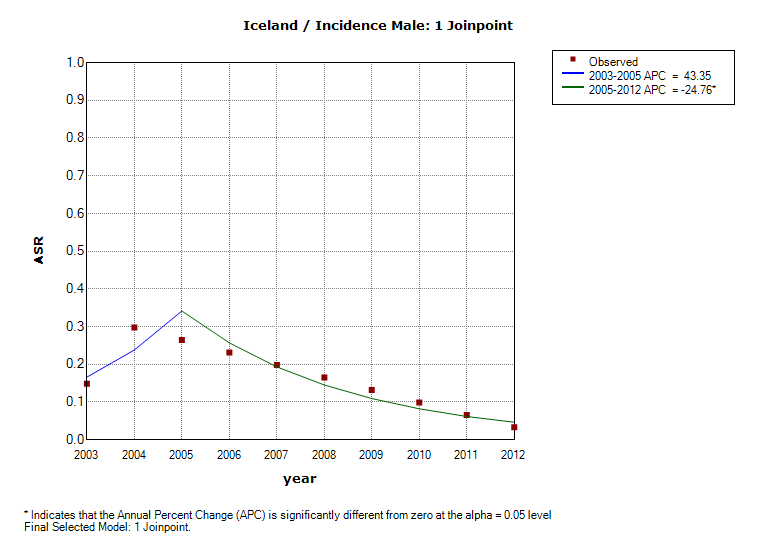 | 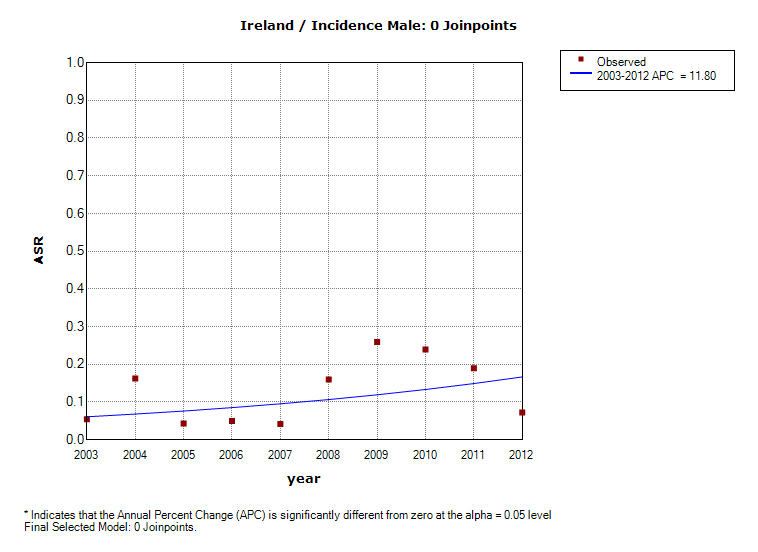 |
| 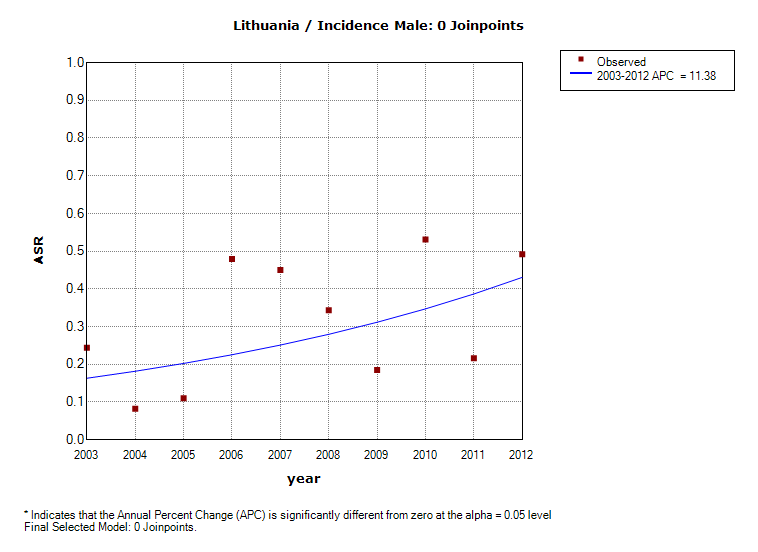 | 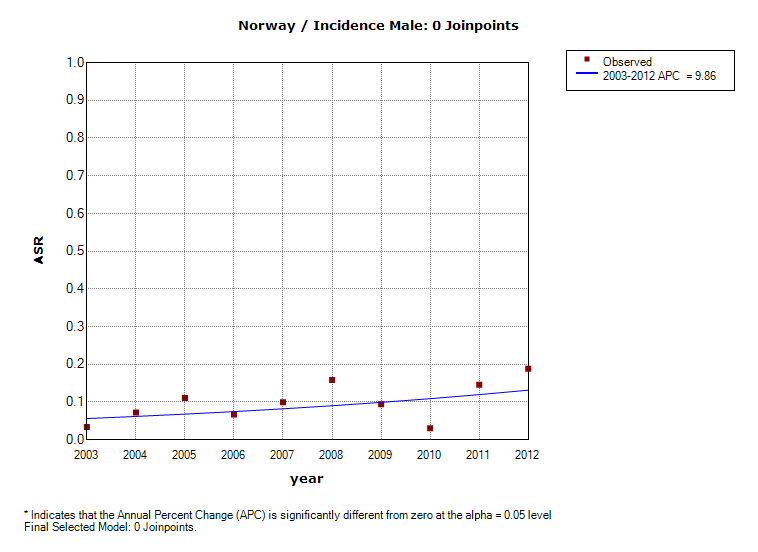 |
| 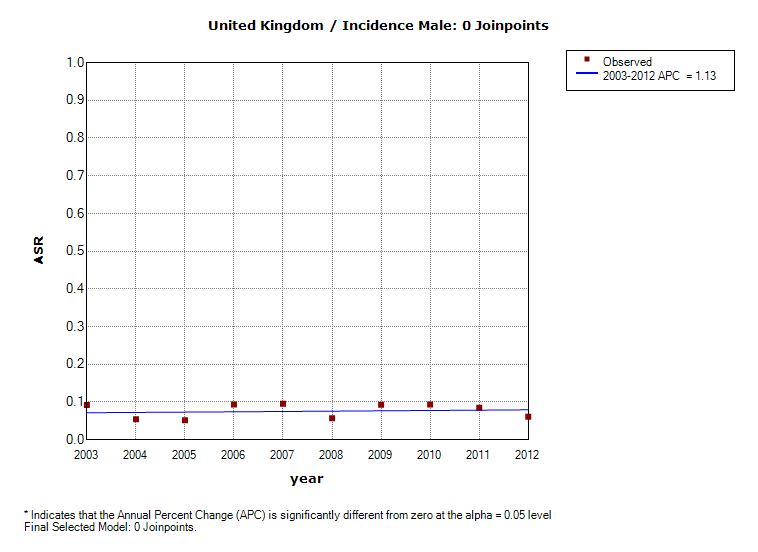 |  |

| **Western Europe** | |
| --- | --- |
| 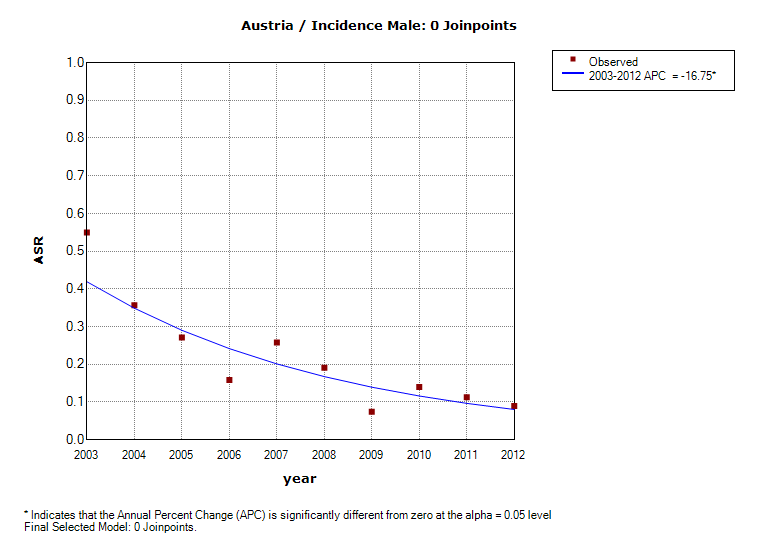 | 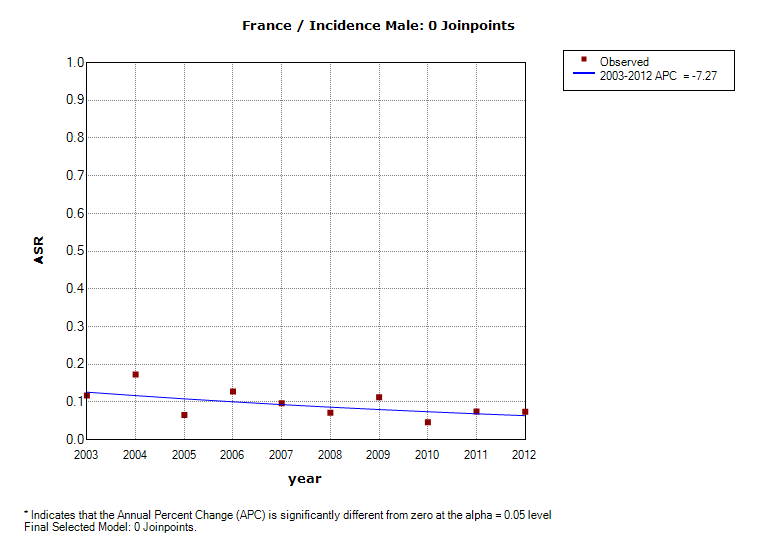 |
| 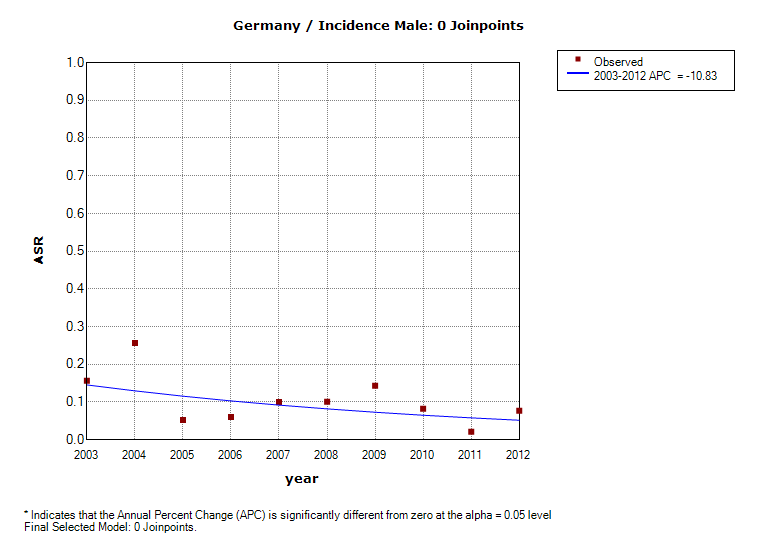 | 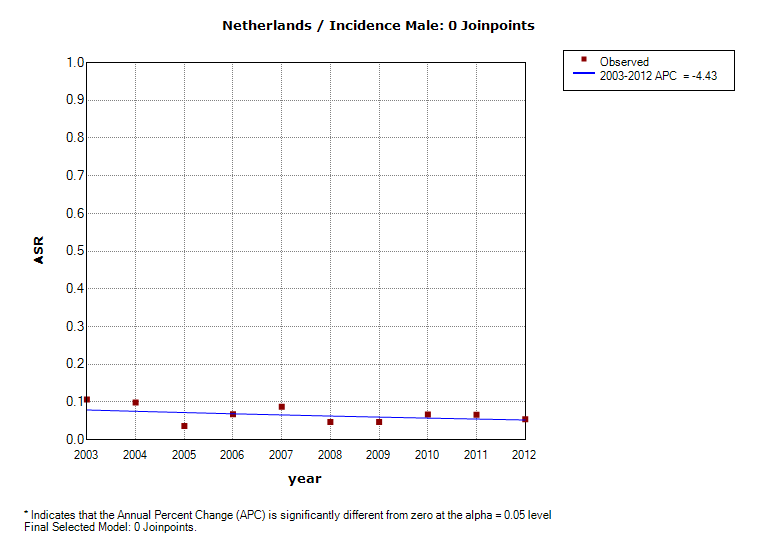 |
| 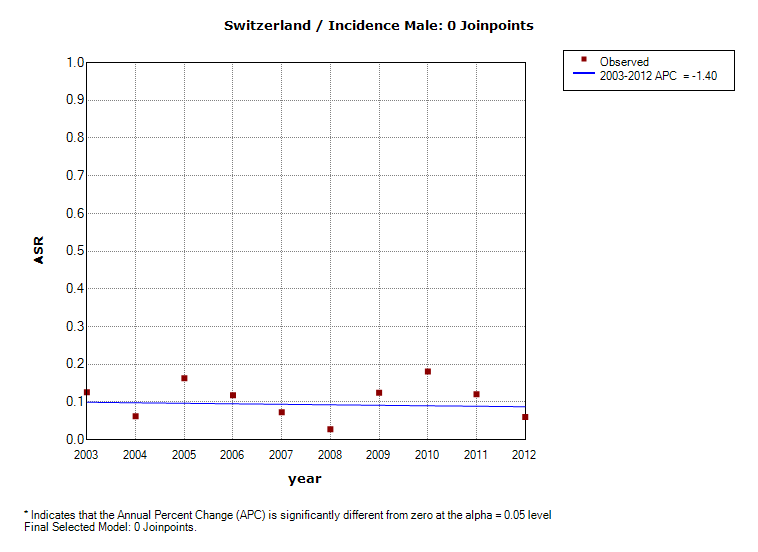 |  |

| **Southern Europe** | |
| --- | --- |
| 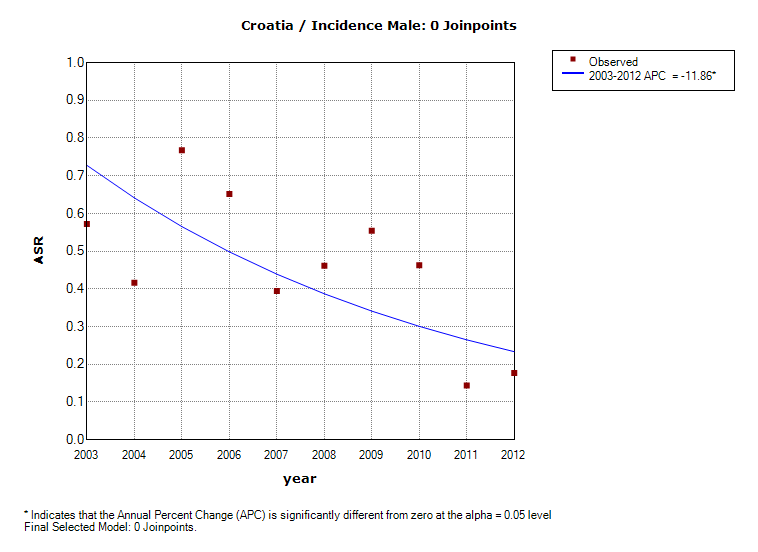 | 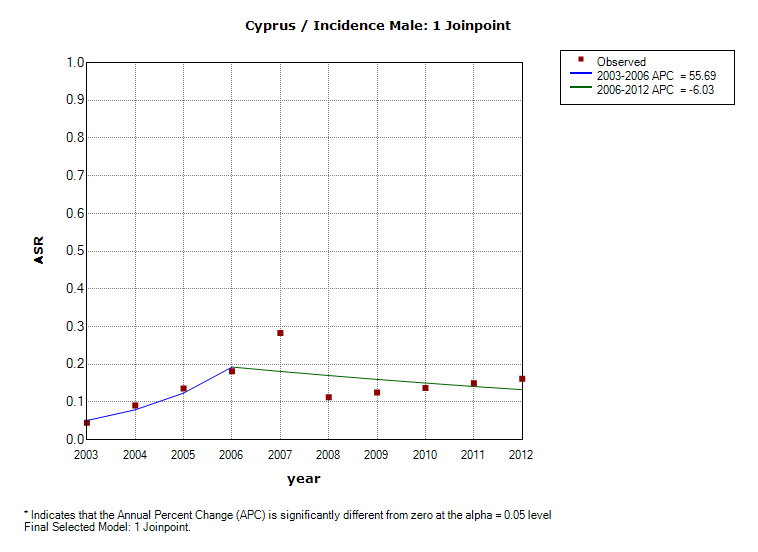 |
| 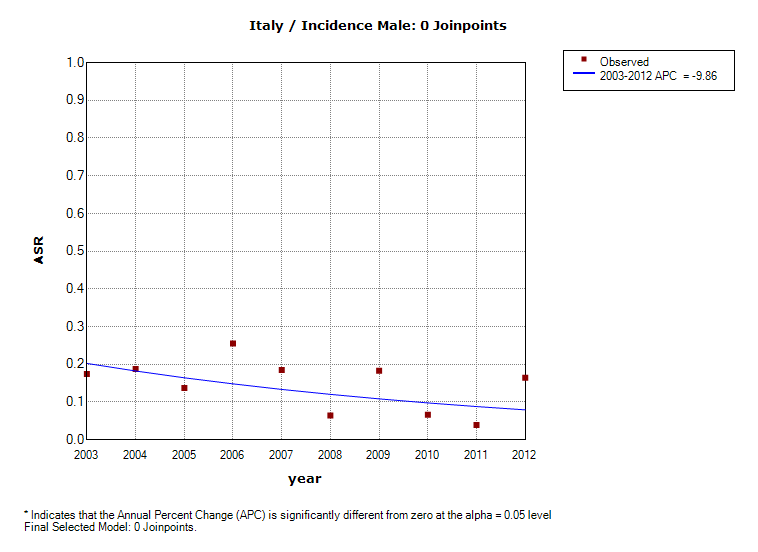 | 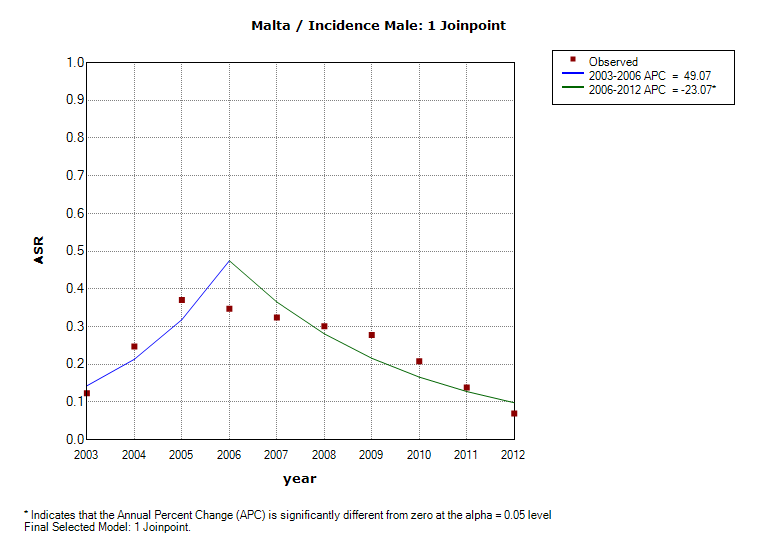 |
| 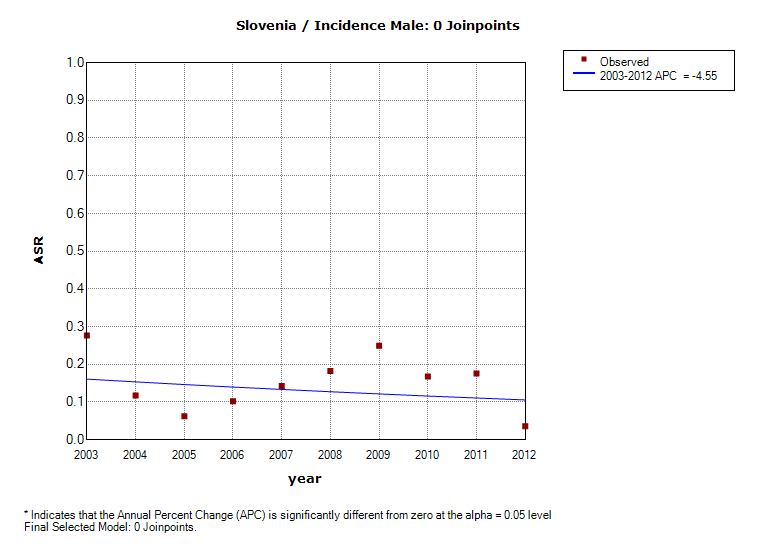 | 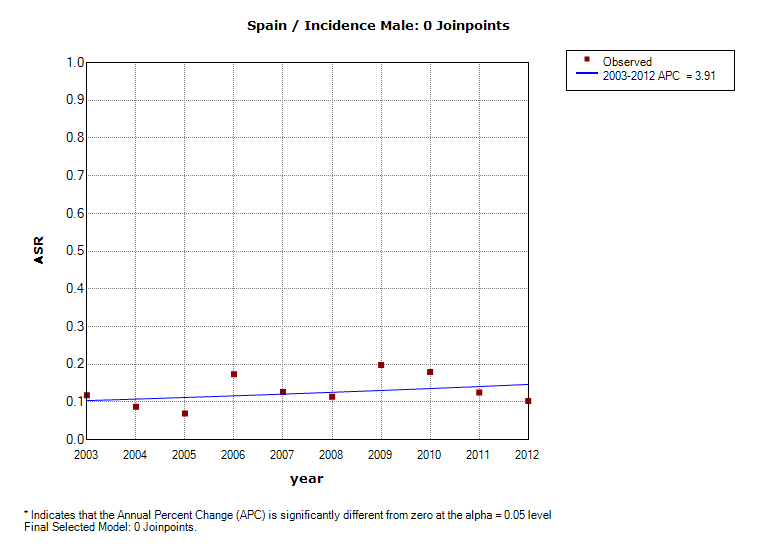 |

| **Eastern Europe** | |
| --- | --- |
| 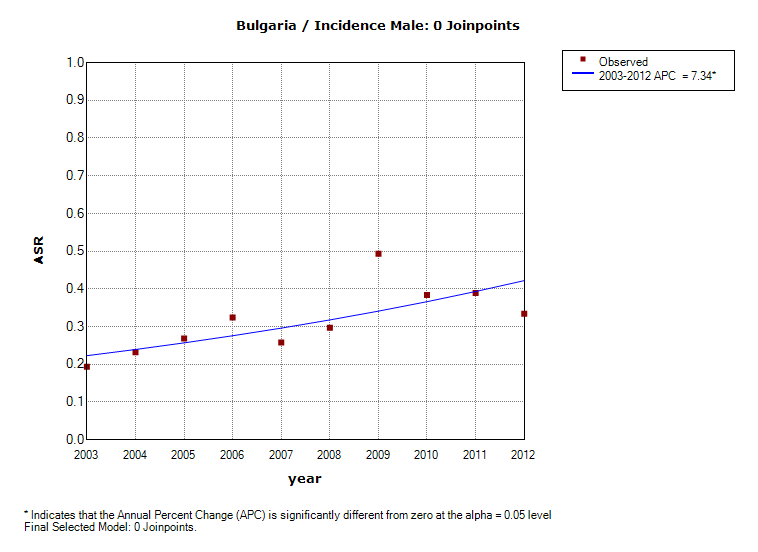 | 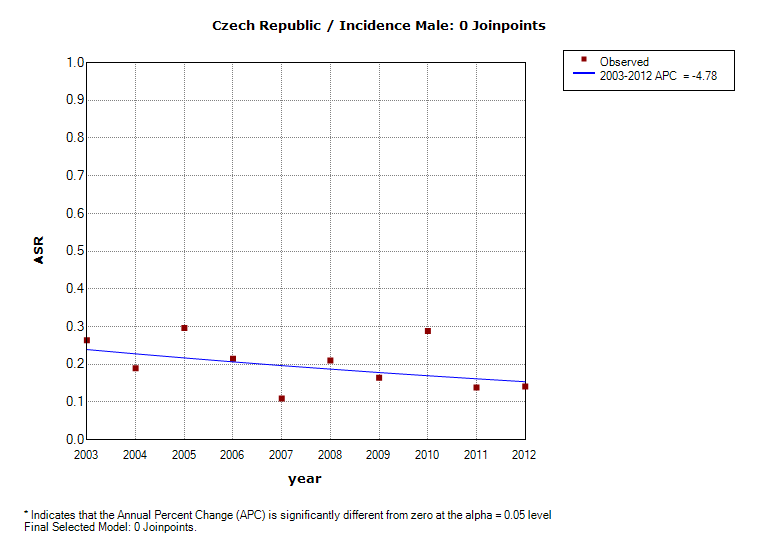 |
| 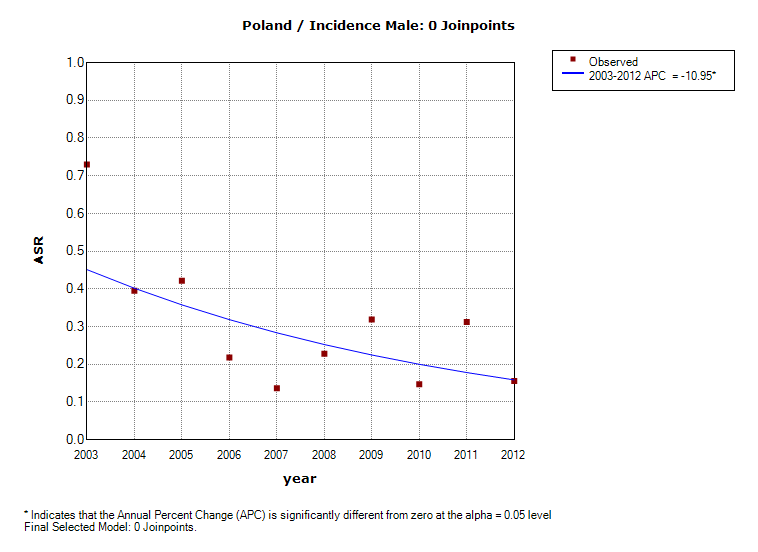 |  |
| **Africa** | |
| 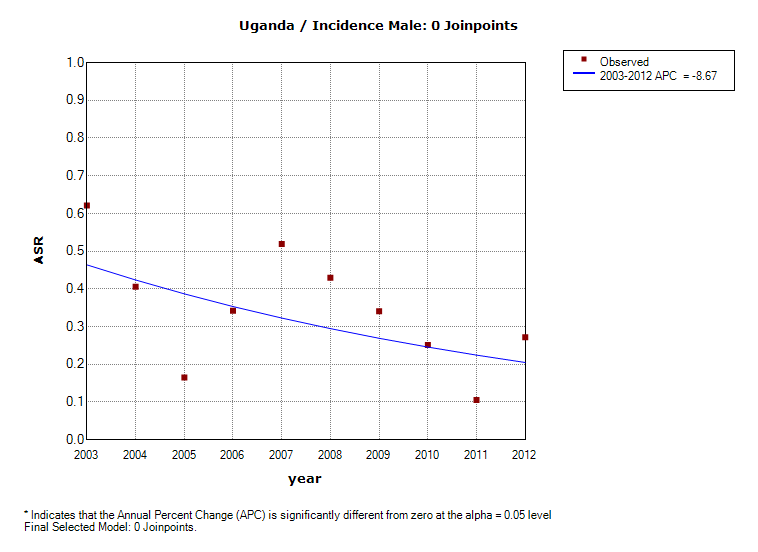 |  |

**b. Female**

| **Asia** | |
| --- | --- |
| 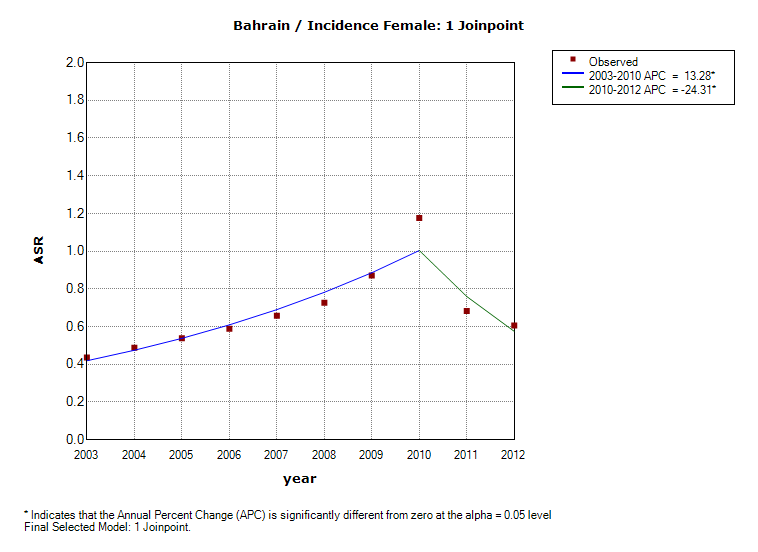 | 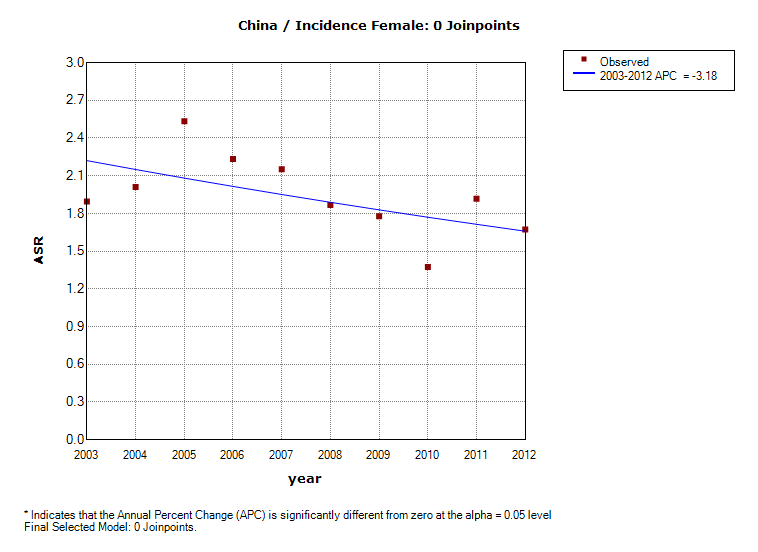 |
| 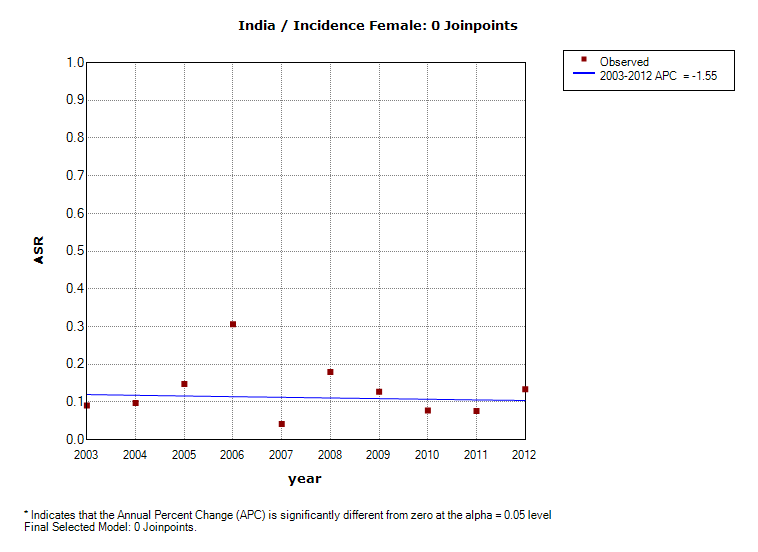 | 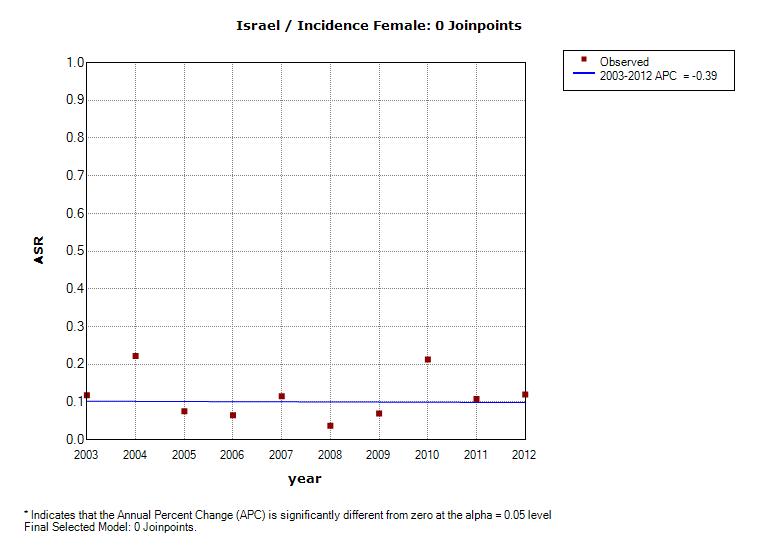 |
| 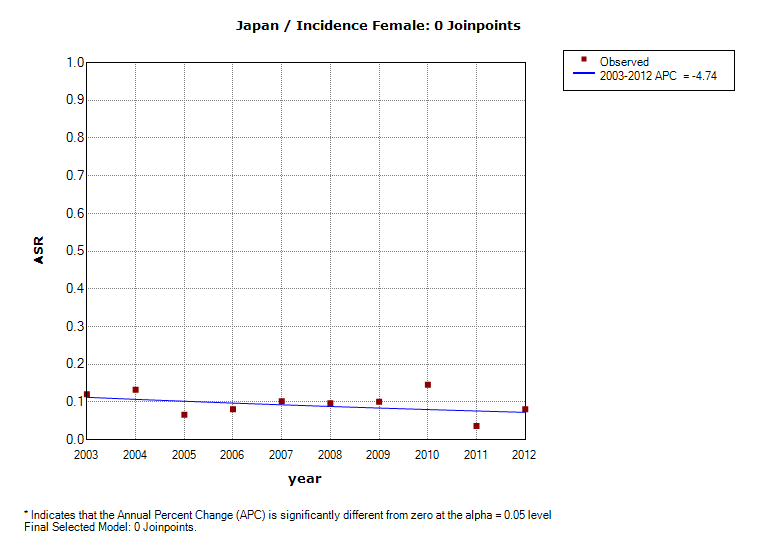 | 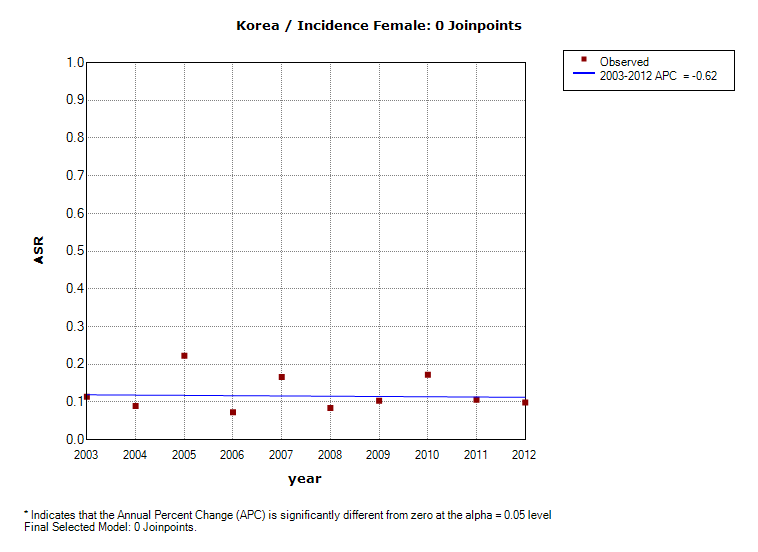 |
| 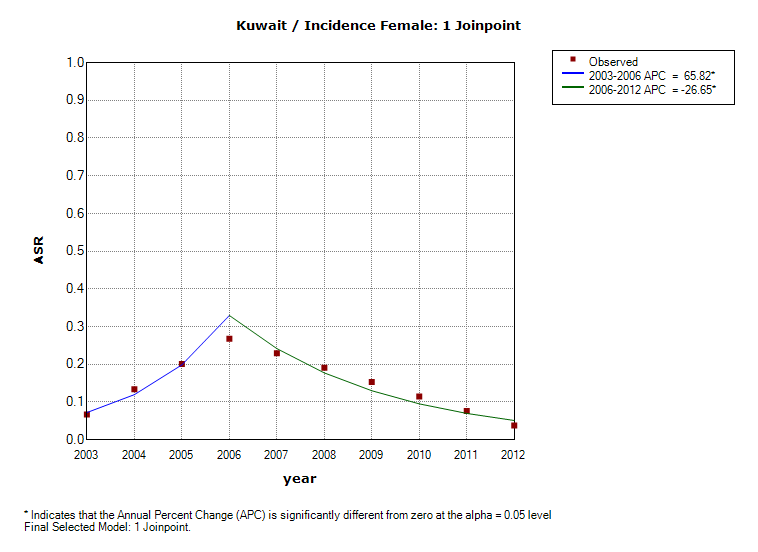 | 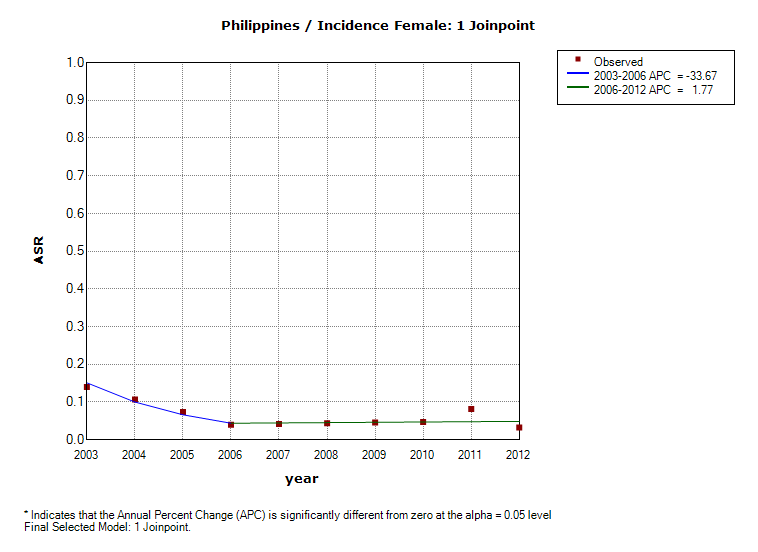 |
| 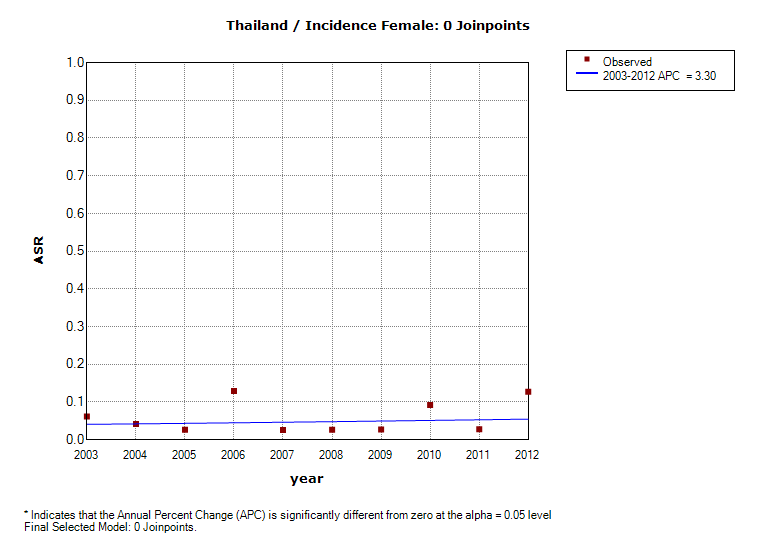 | 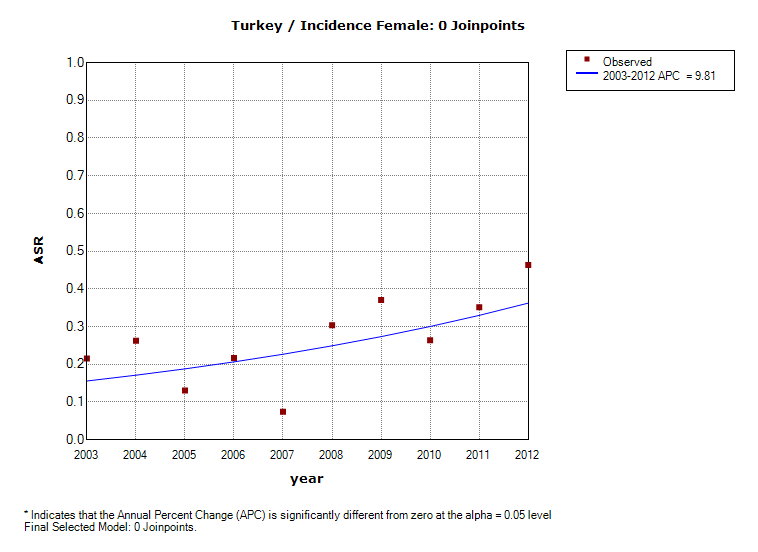 |
| **Oceania** | |
| 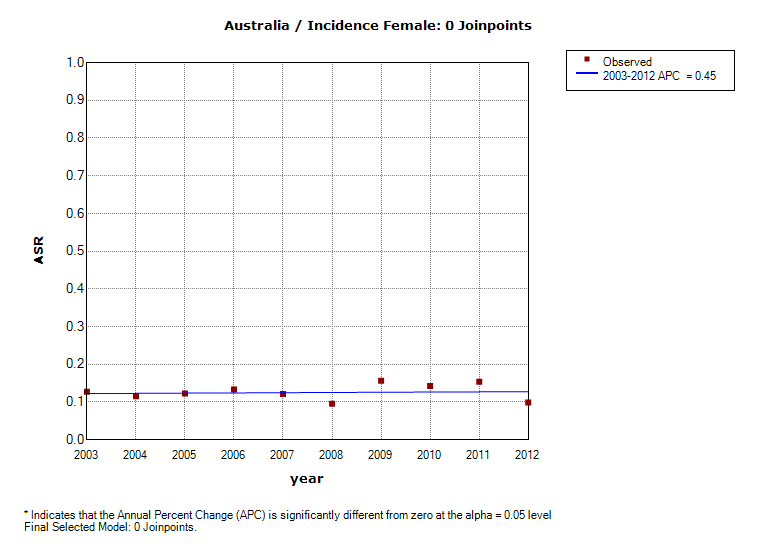 | 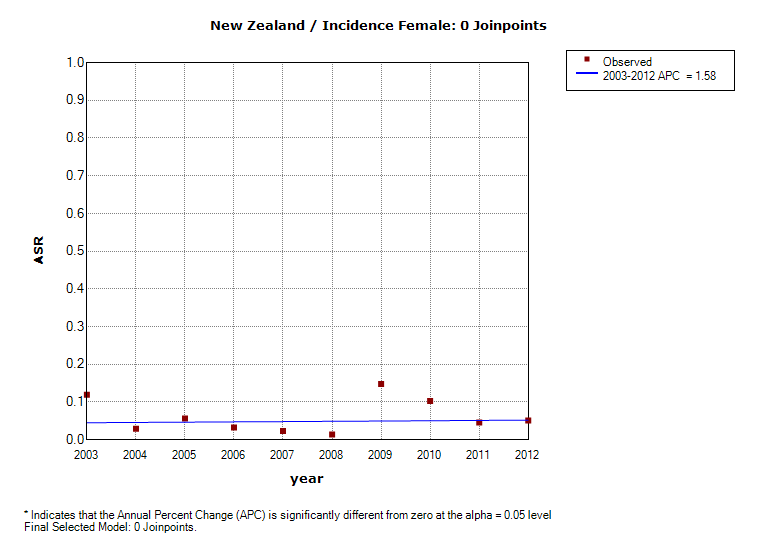 |

| **Northern America** | |
| --- | --- |
| 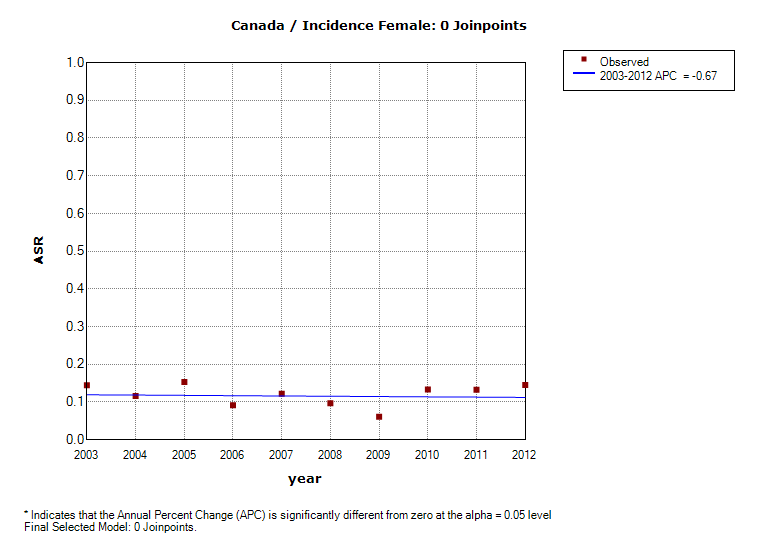 | 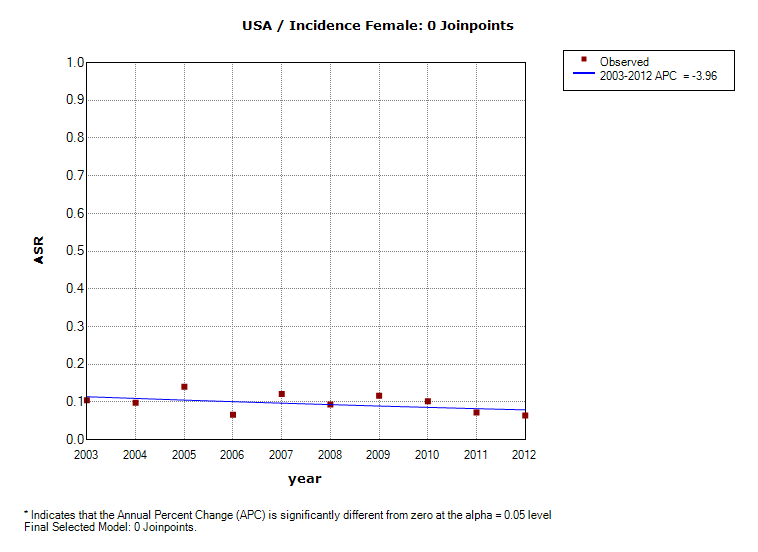 |
| **Southern America** | |
| 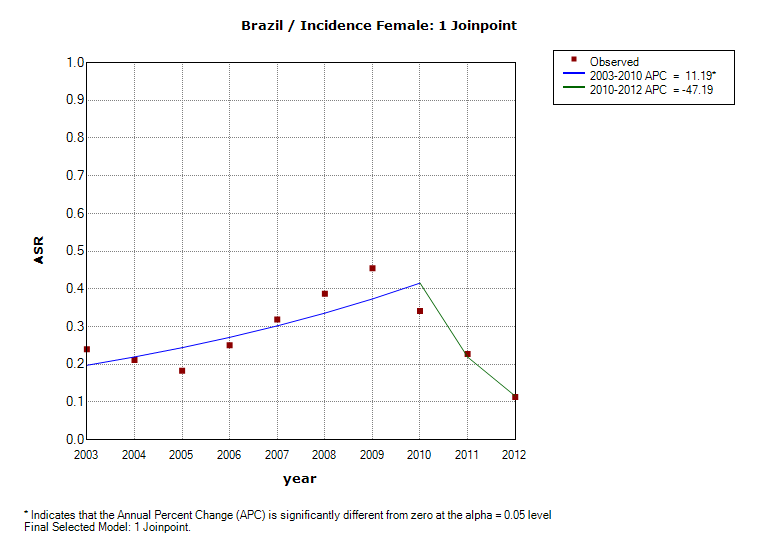 | 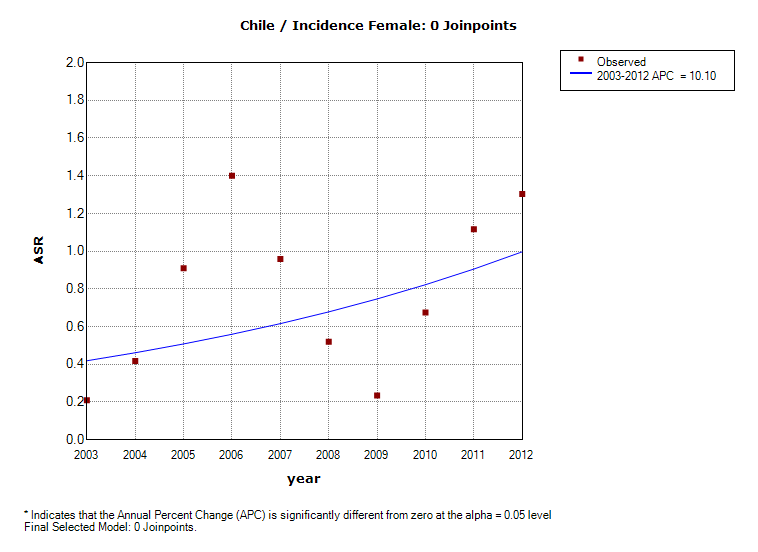 |
| 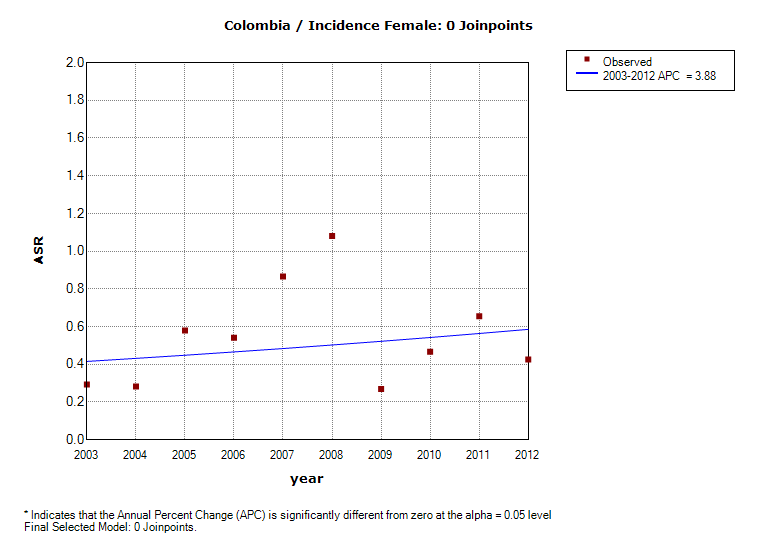 | 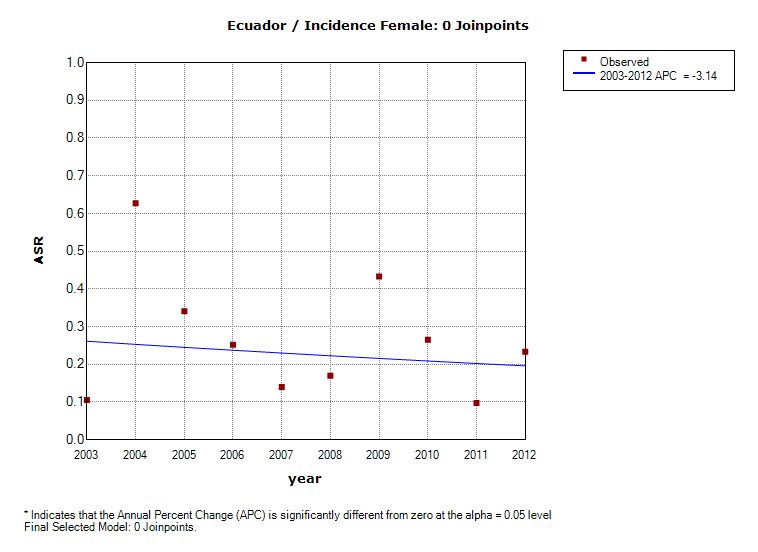 |
| 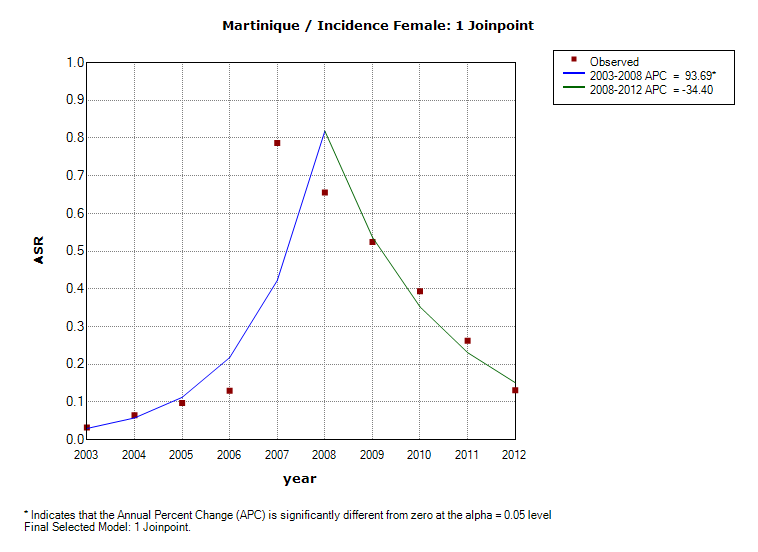 |  |

| **Northern Europe** | |
| --- | --- |
| 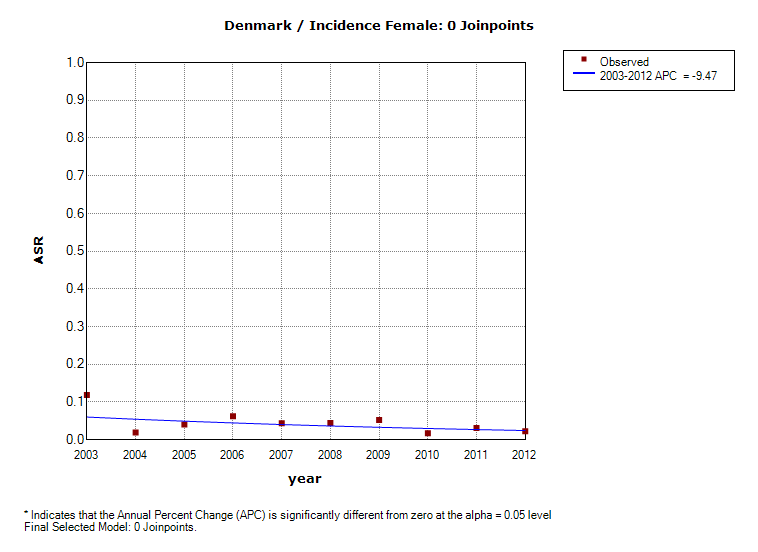 | 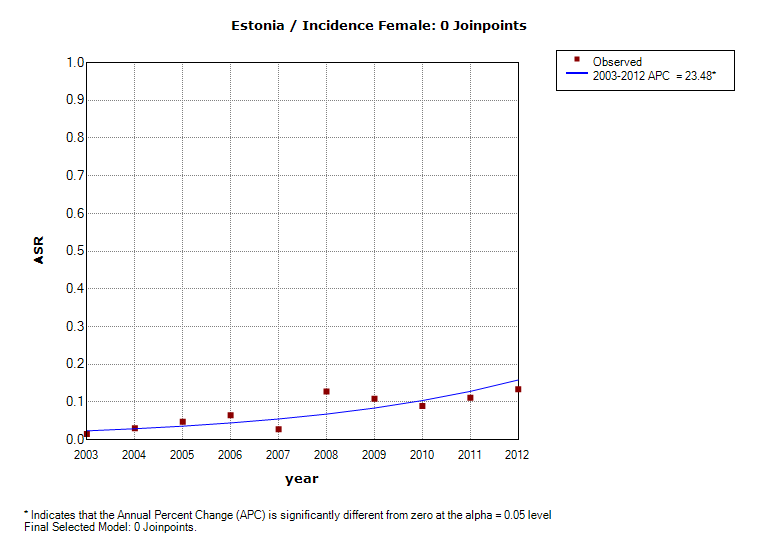 |
| 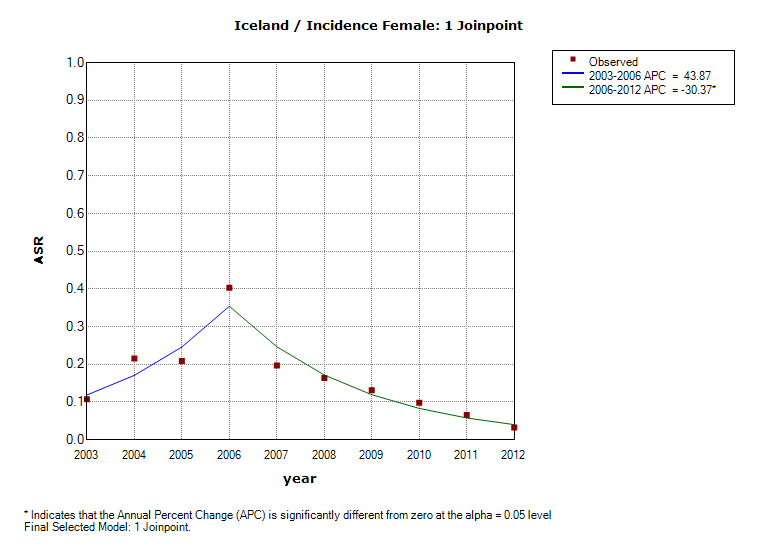 | 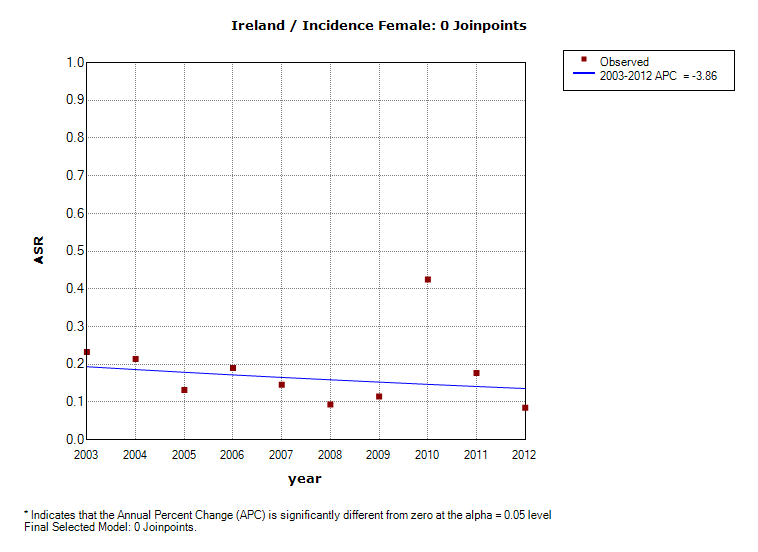 |
| 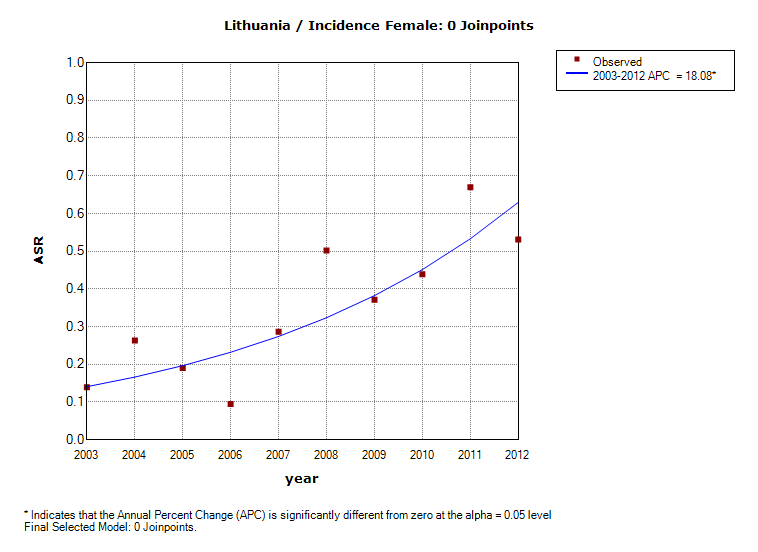 | 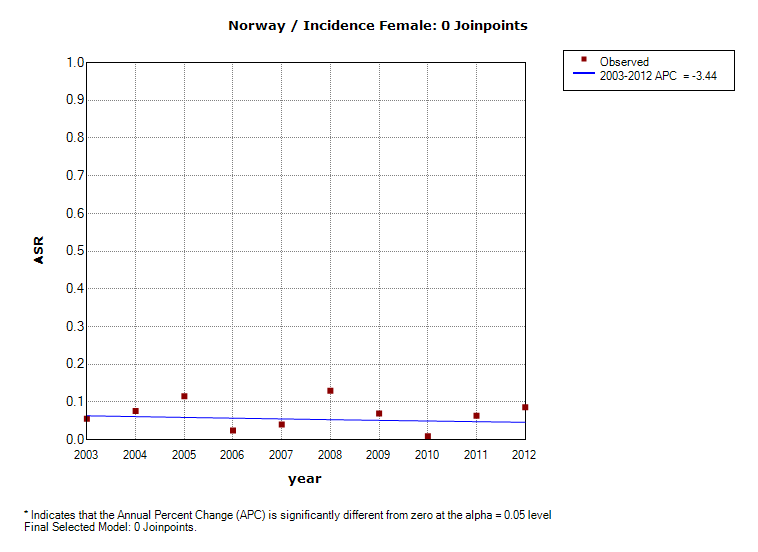 |
| 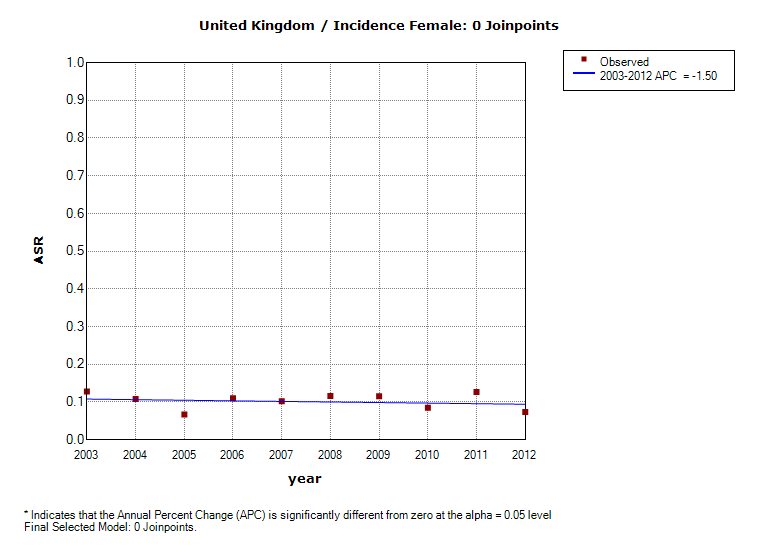 |  |
| **Western Europe** | |
| 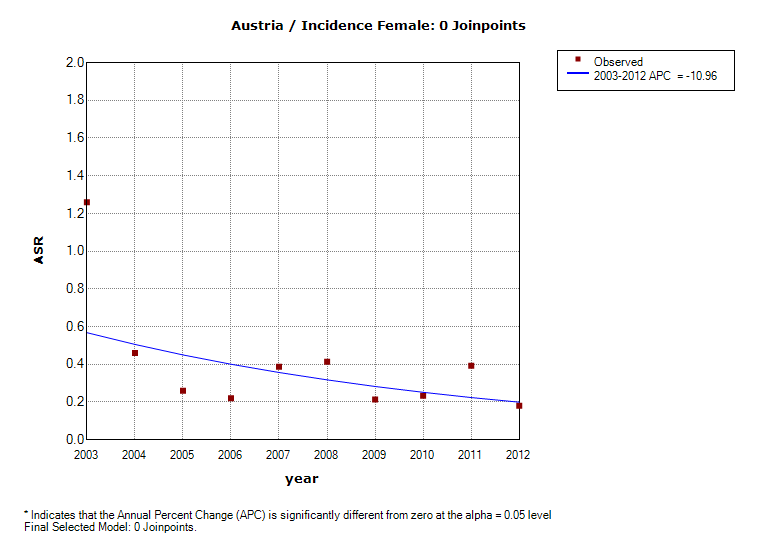 | 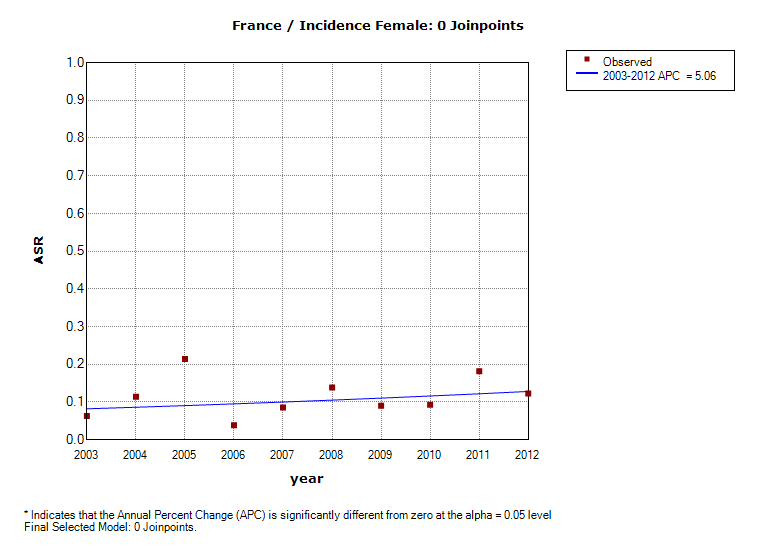 |
| 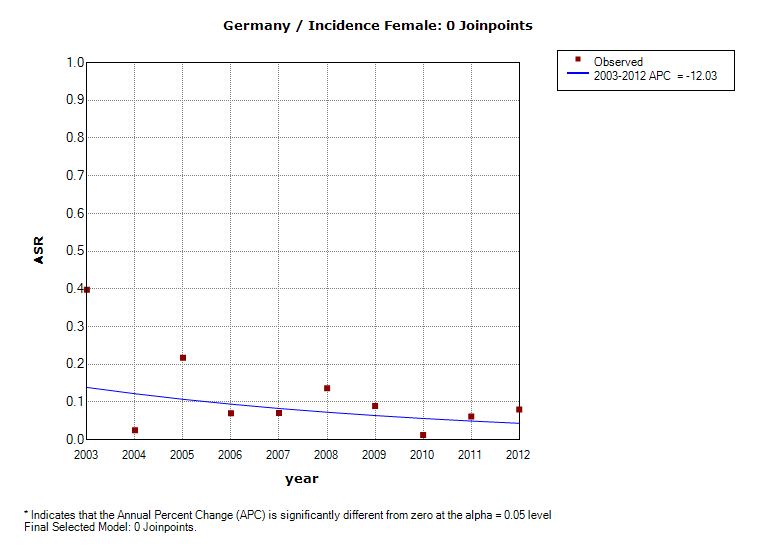 | 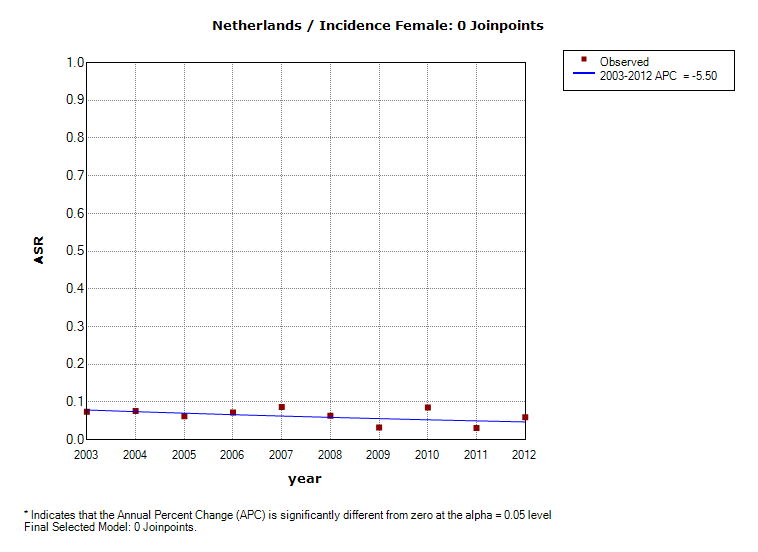 |
| 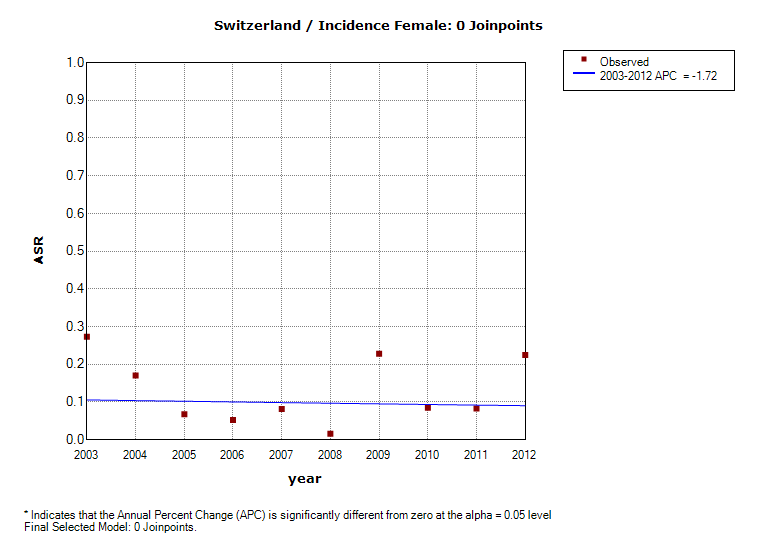 |  |

| **Southern Europe** | |
| --- | --- |
| 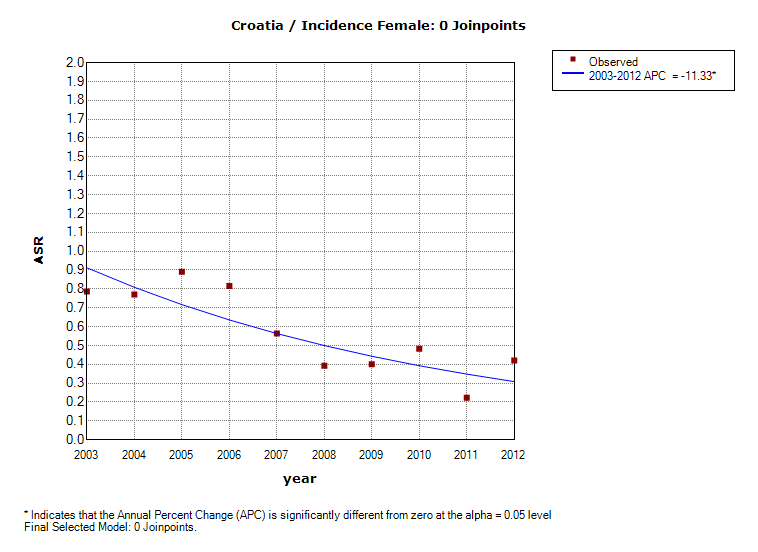 | 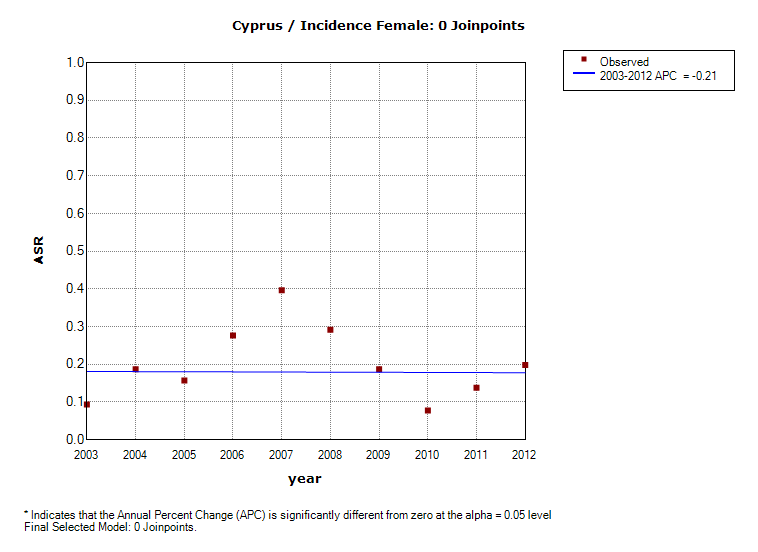 |
| 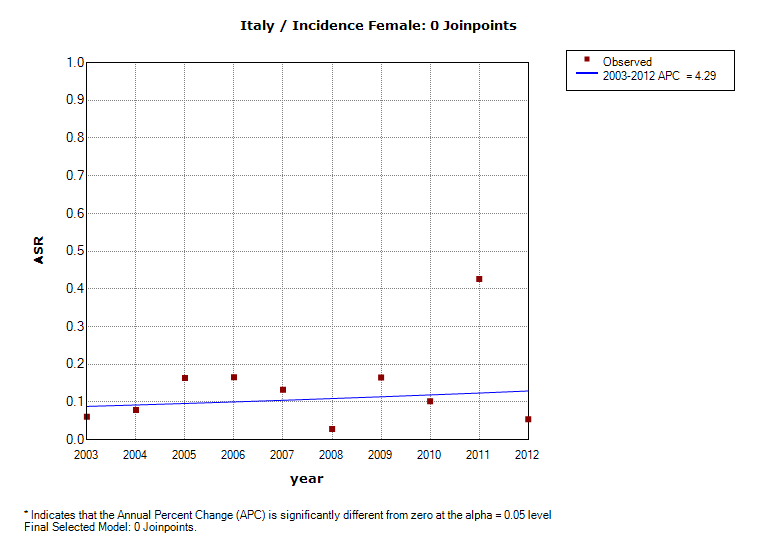 | 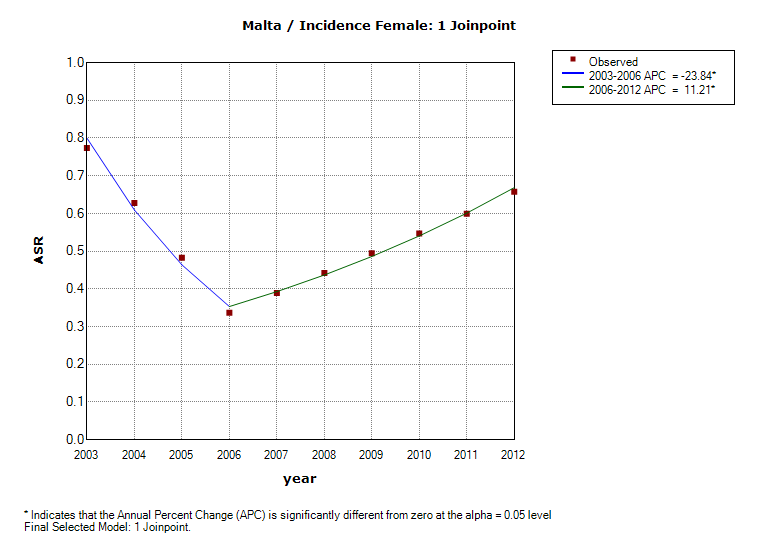 |
| 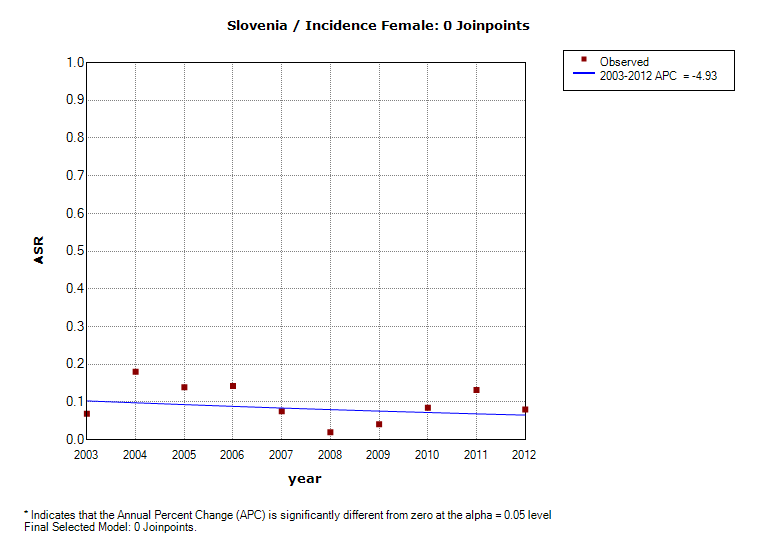 | 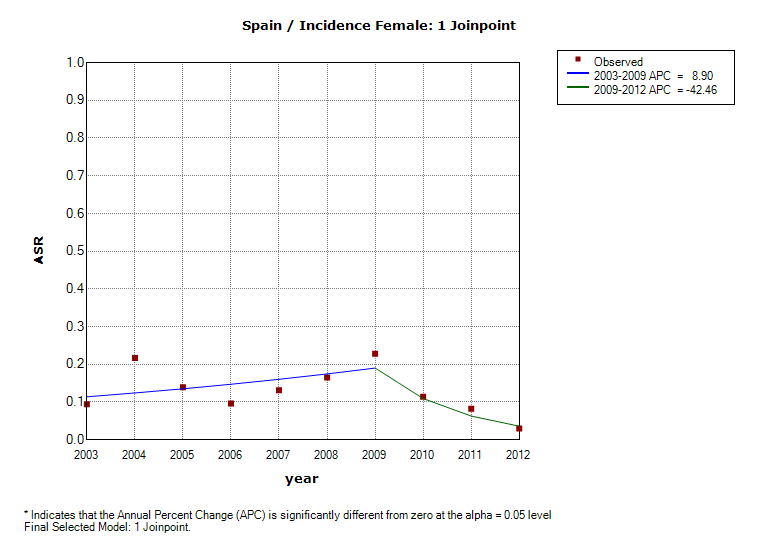 |

| **Eastern Europe** | |
| --- | --- |
| 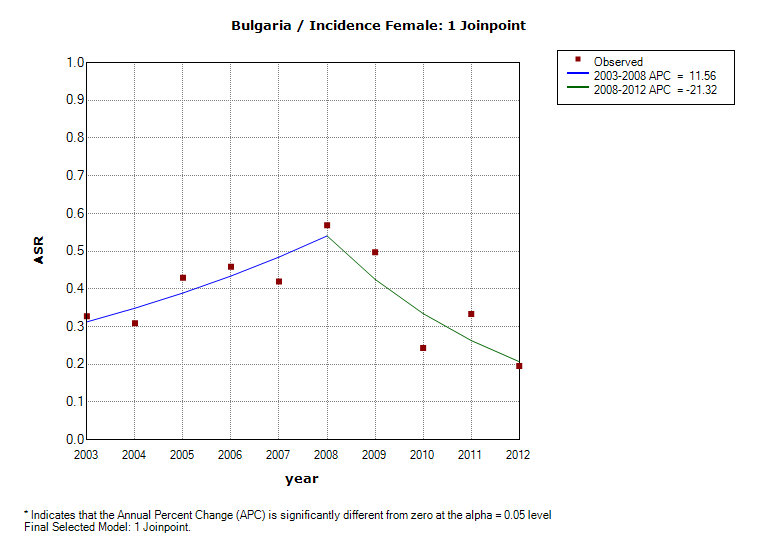 | 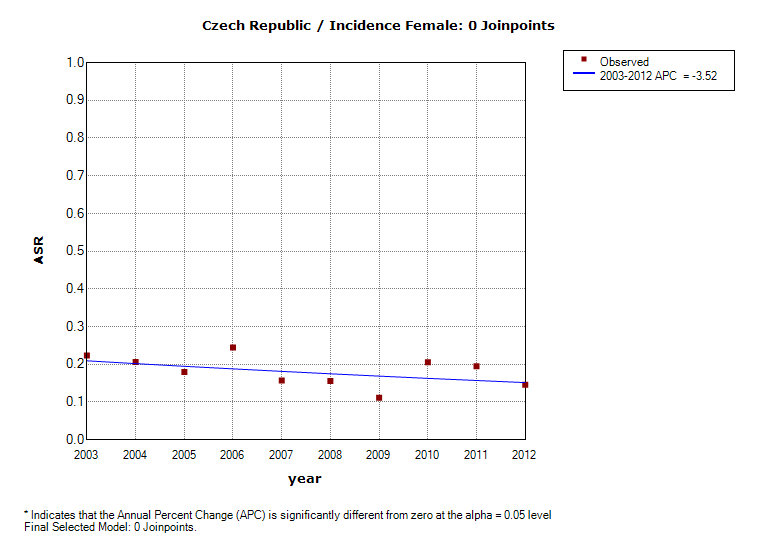 |
| 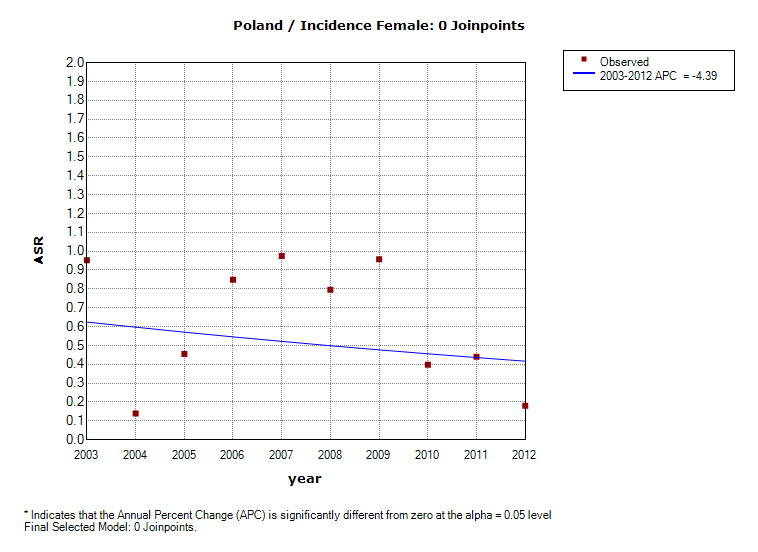 |  |
| **Africa** | |
| 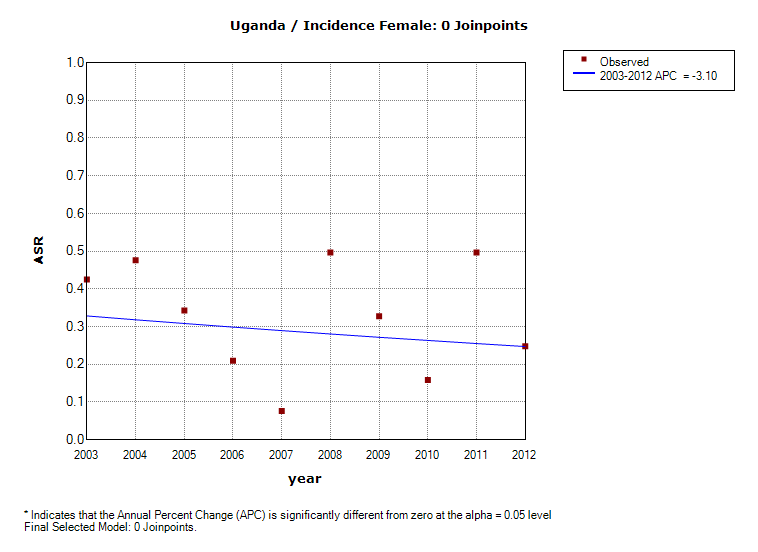 |  |

**c. Both**

| **Asia** | |
| --- | --- |
| 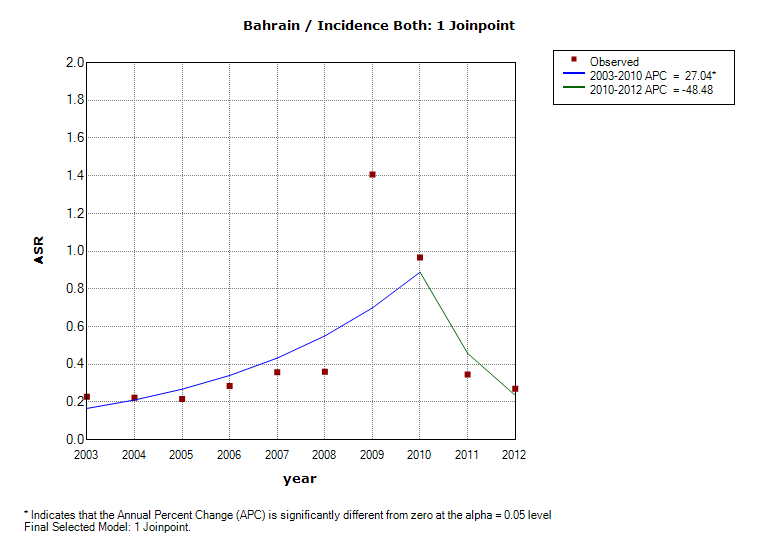 | 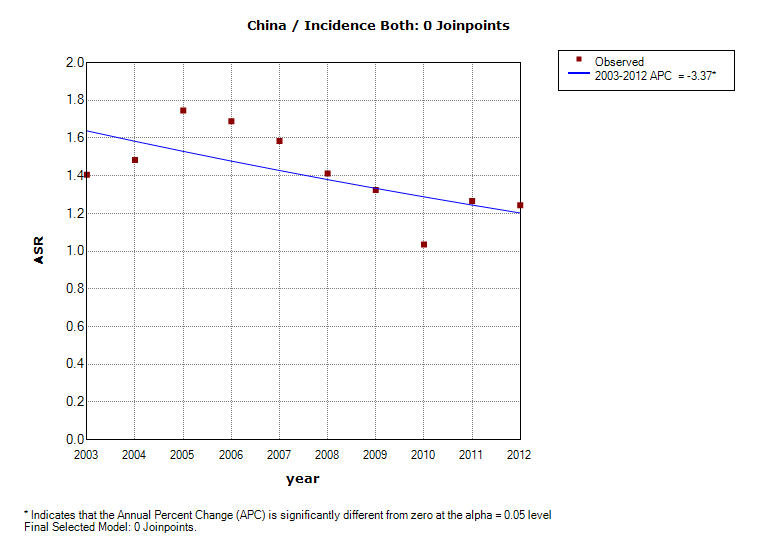 |
| 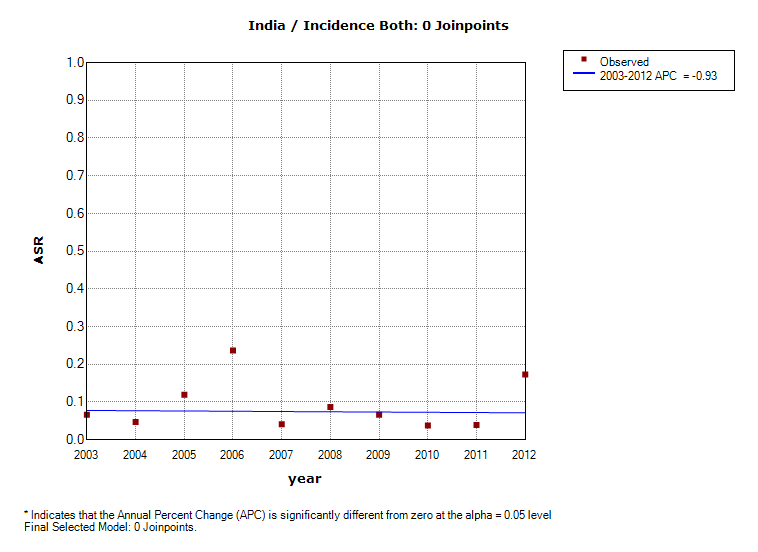 | 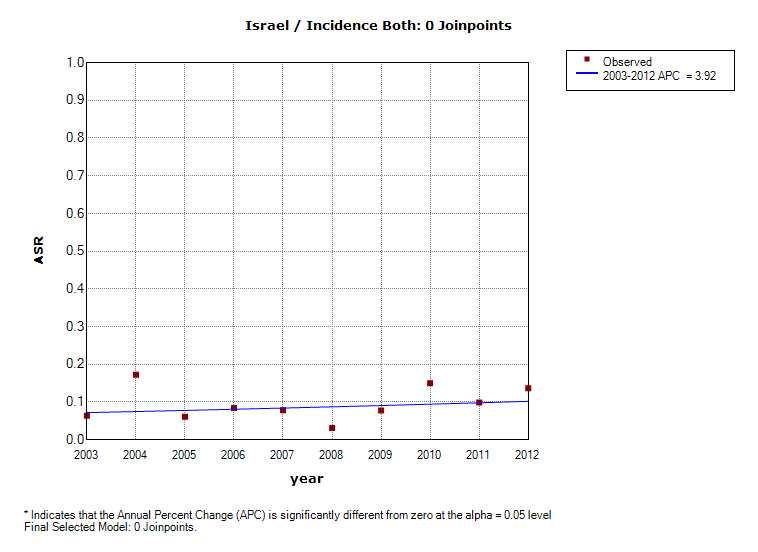 |
| 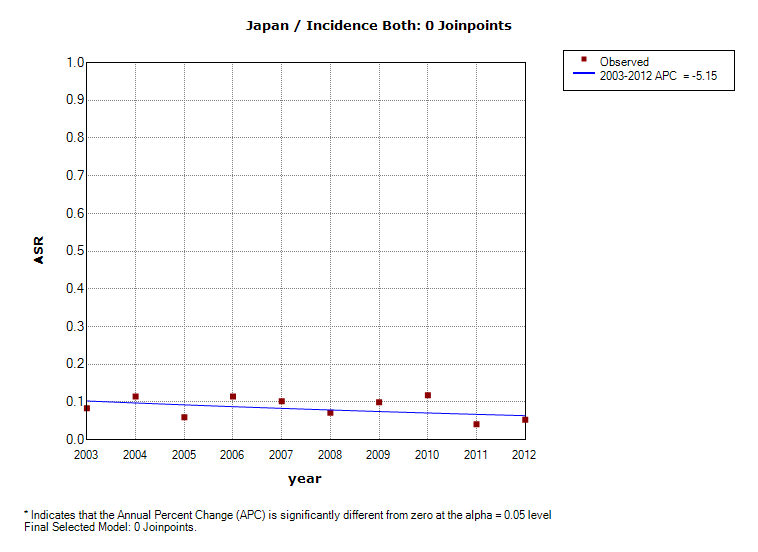 | 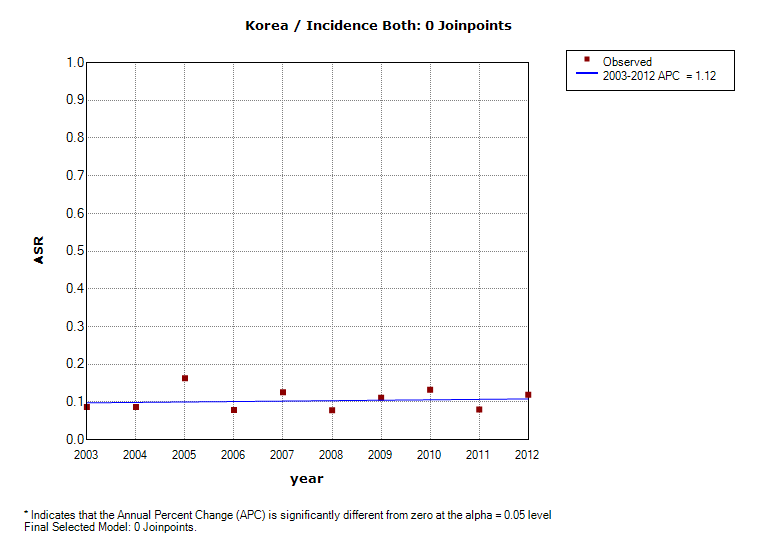 |
| 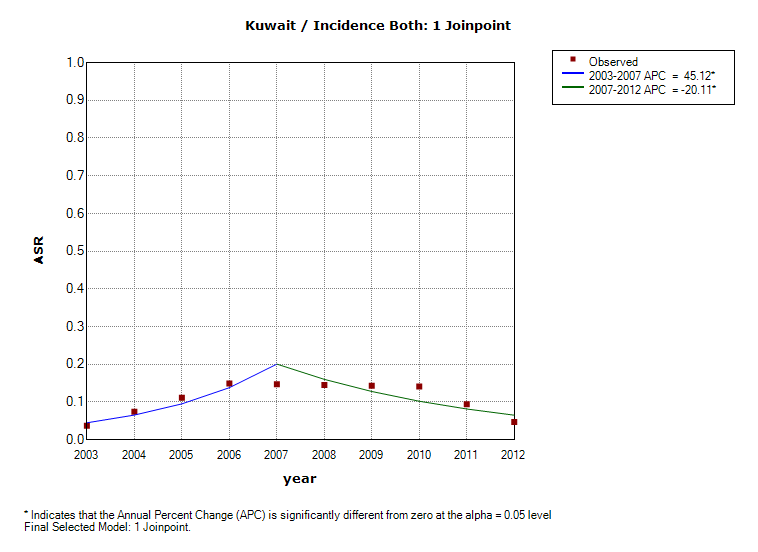 | 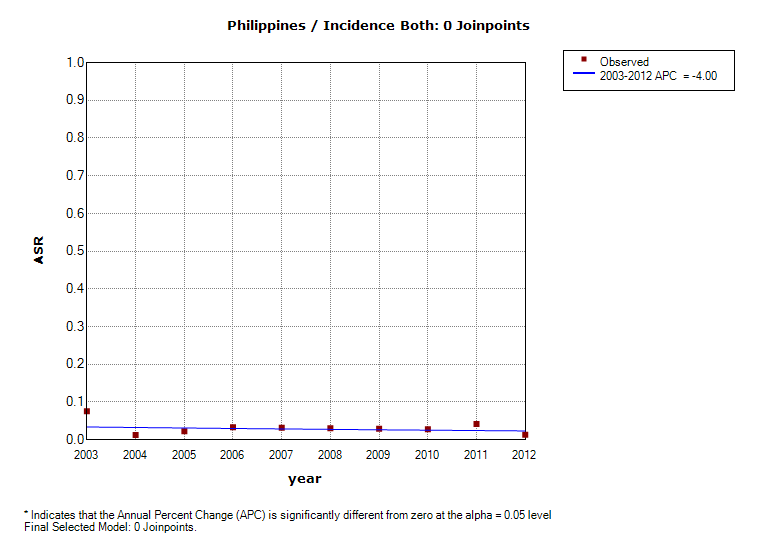 |
| 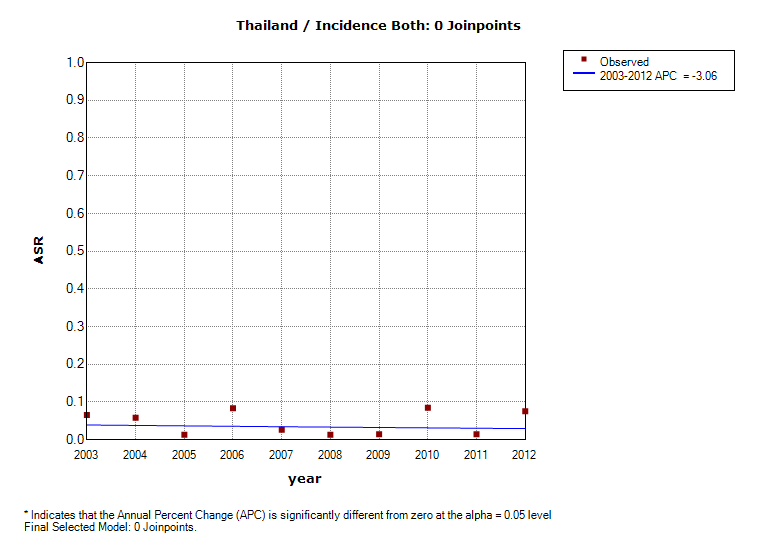 | 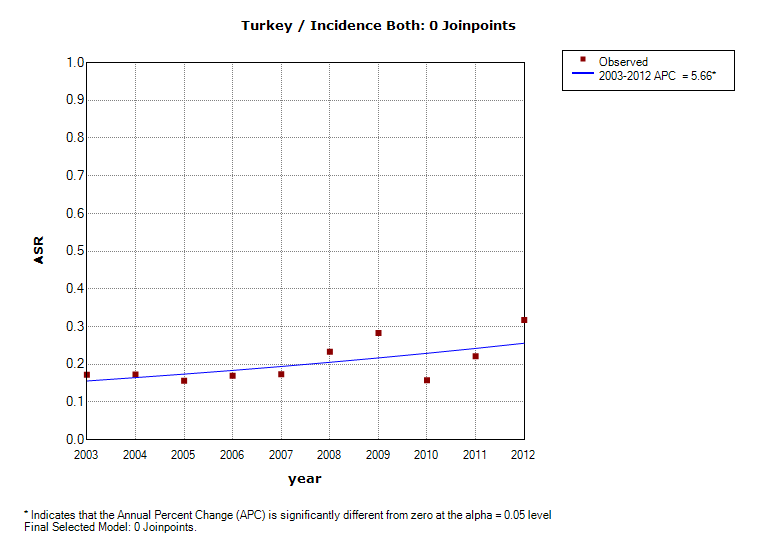 |
| **Oceania** | |
| 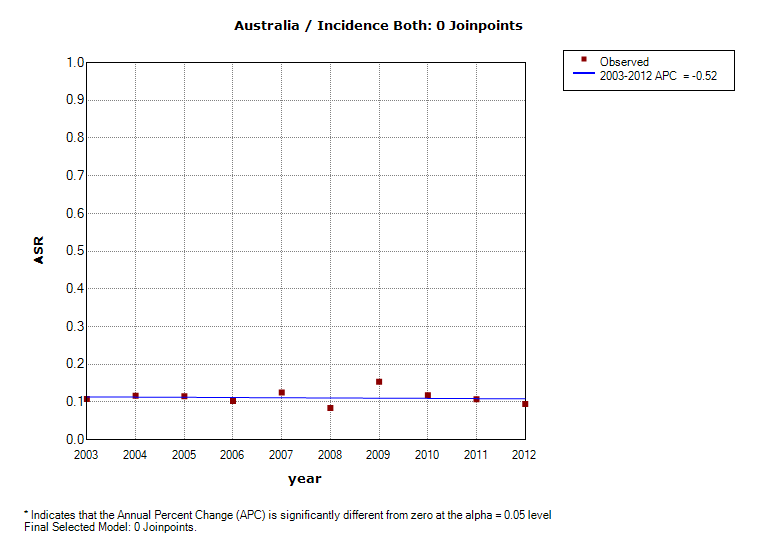 | 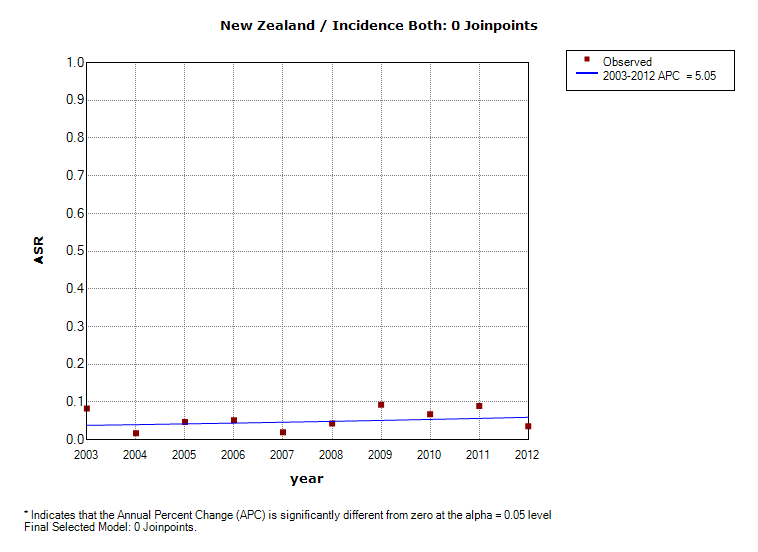 |

| **Northern America** | |
| --- | --- |
| 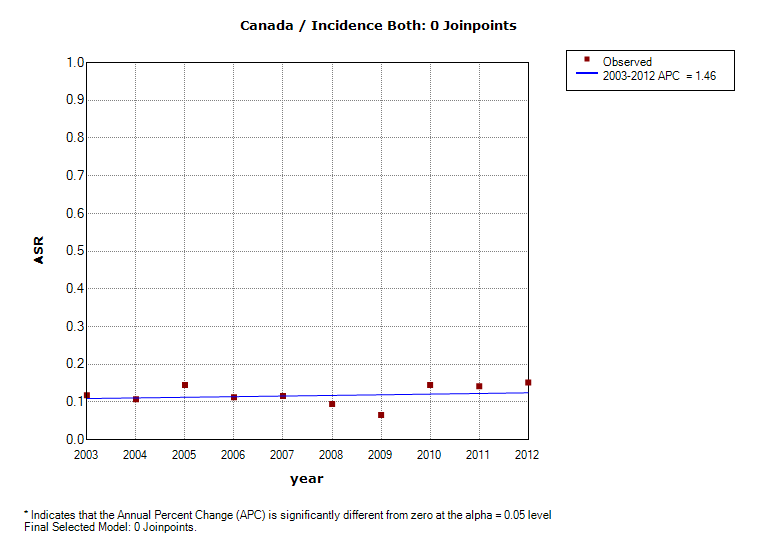 | 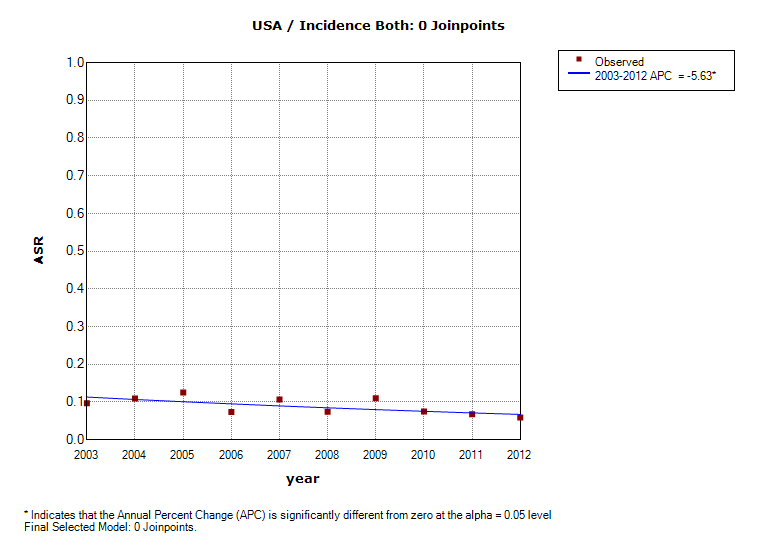 |
| **Southern America** | |
| 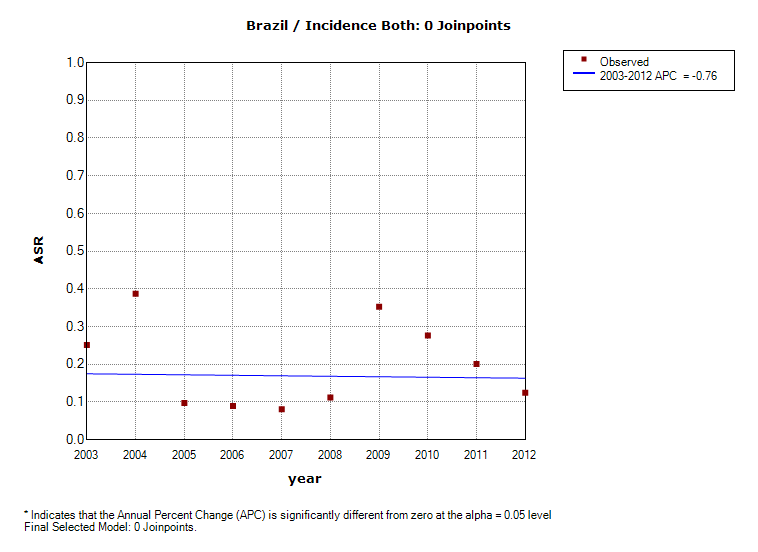 | 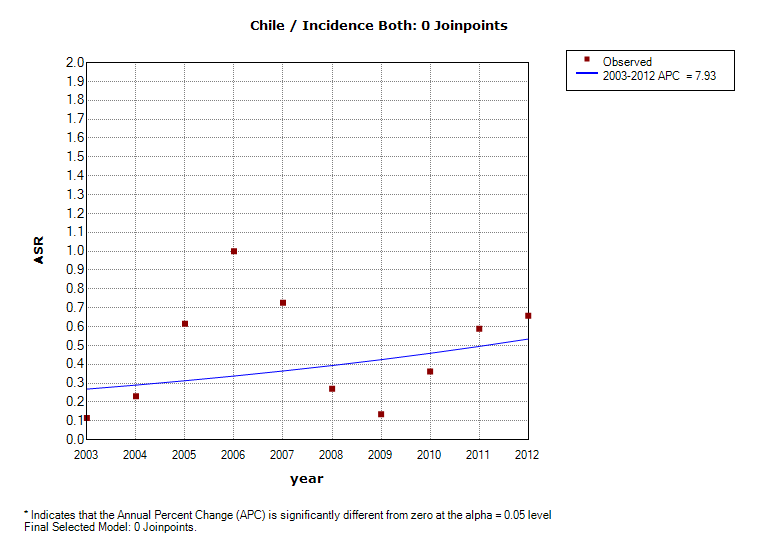 |
| 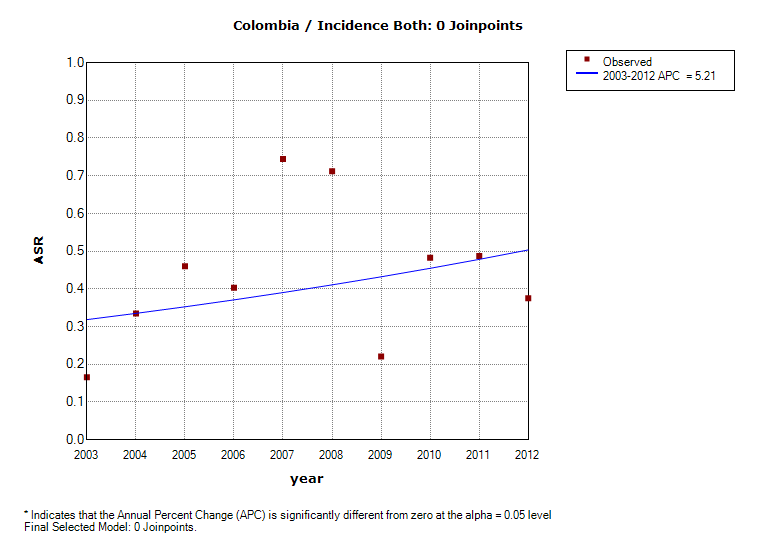 | 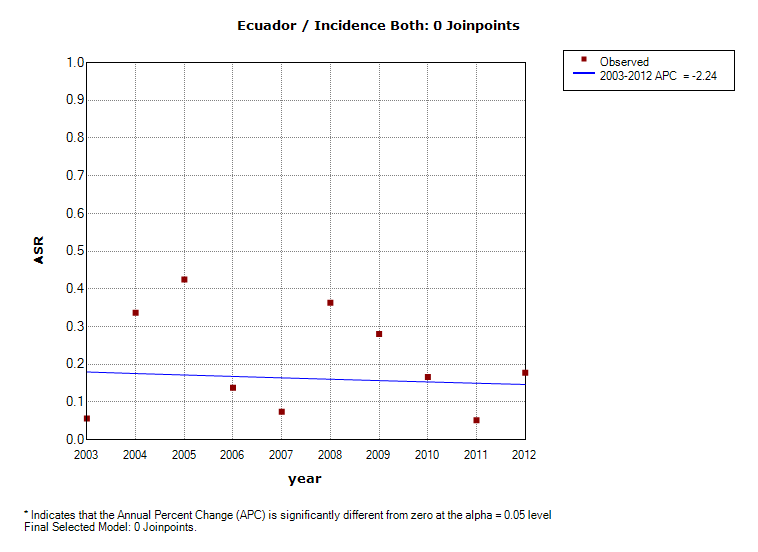 |
|  |  |

| **Northern Europe** | |
| --- | --- |
|  |  |
|  |  |
|  |  |
|  |  |

| **Western Europe** | |
| --- | --- |
|  |  |
|  |  |
|  |  |

| **Southern Europe** | |
| --- | --- |
|  |  |
|  |  |
|  |  |

| **Eastern Europe** | |
| --- | --- |
|  |  |
|  |  |
| **Africa** | |
|  |  |

**d. Young**

| **Asia** | |
| --- | --- |
|  |  |
|  |  |
|  |  |
|  |  |
|  |  |
| **Oceania** | |
|  |  |

| **Northern America** | |
| --- | --- |
|  |  |
| **Southern America** | |
|  |  |
|  |  |
|  |  |

| **Northern Europe** | |
| --- | --- |
|  |  |
|  |  |
|  |  |

| **Western Europe** | |
| --- | --- |
|  |  |
|  |  |
|  |  |

| **Southern Europe** | |
| --- | --- |
|  |  |
|  |  |
|  |  |

| **Eastern Europe** | |
| --- | --- |
|  |  |
|  |  |
| **Africa** | |
|  |  |

**e. Old**

| **Asia** | |
| --- | --- |
|  |  |
|  |  |
|  |  |
|  |  |
|  |  |
| **Oceania** | |
|  |  |

| **Northern America** | |
| --- | --- |
|  |  |
| **Southern America** | |
|  |  |
|  |  |
|  |  |

| **Northern Europe** | |
| --- | --- |
|  |  |
|  |  |
|  |  |

| **Western Europe** | |
| --- | --- |
|  |  |
|  |  |
|  |  |

| **Southern Europe** | |
| --- | --- |
|  |  |
|  |  |
|  |  |

| **Eastern Europe** | |
| --- | --- |
|  |  |
|  |  |
| **Africa** | |
|  |  |
